# Educational Intervention of Healthy Life Promotion for Children with a Migrant Background or at Socioeconomic Disadvantage in the North of Italy: Efficacy of Telematic Tools in Improving Nutritional and Physical Activity Knowledge

**DOI:** 10.3390/nu13103634

**Published:** 2021-10-17

**Authors:** Roberto Franceschi, Elena Fornari, Monica Ghezzi, Eleonora Buzzi, Margherita Toschi, Silvia Longhi, Rosa Maimone, Stefano Forti, Sara Carneri, Fateh Moghadam Pirous, Beatrice Agostini, Tommaso Iori, Marta Gibin, Stefania Porchia, Massimo Soffiati, Claudio Maffeis

**Affiliations:** 1 Division of Pediatrics, S. Chiara General Hospital, 38122 Trento, Italy; monica.ghezzi@apss.tn.it (M.G.); massimo.soffiati@apss.tn.it (M.S.); 2 Section of Pediatric Diabetes and Metabolism, Department of Surgery, Dentistry, Gynecology and Pediatrics, University and Azienda Ospedaliera Universitaria Integrata of Verona, 37126 Verona, Italy; elena.fornari@aovr.veneto.it (E.F.); dott.eleonorabuzzi@gmail.com (E.B.); margheritatoschinutrizione@gmail.com (M.T.); claudio.maffeis@univr.it (C.M.); 3 Division of Pediatrics, General Hospital Bolzano, 39100 Bolzano, Italy; silvia.longhi@sabes.it; 4 Center for Information and Communication Technology, eHealth Unit, Fondazione Bruno Kessler, 38123 Trento, Italy; rmaimone@fbk.eu (R.M.); forti@fbk.eu (S.F.); 5 Health Observatory, Autonomous Province of Trento, 38121 Trento, Italy; saracarneri@gmail.com (S.C.); pirous.fatehmoghadam@apss.tn.it (F.M.P.); 6 Unione Italiana Sport per Tutti, Comitato Territoriale Trentino APS, 38121 Trento, Italy; beatrice.agostini@gmail.com (B.A.); t.iori@uisp.it (T.I.); 7 Research Institute for Social Innovation, Ca’ Foscari University, 30123 Venice, Italy; marta_mga@hotmail.it (M.G.); stefania.porchia@unive.it (S.P.)

**Keywords:** healthy lifestyle, education, migrant background, socioeconomic disadvantage, overweight

## Abstract

The aim of the “Smuovi La Salute” (“Shake Your Health”) project was to implement an integrated and comprehensive model to prevent and treat overweight and obesity in low socioeconomic status (SES) and minority groups living in three different districts in the north of Italy. An app and a cookbook promoting transcultural nutrition and a healthy lifestyle were developed, and no-cost physical activities were organized. Healthy lifestyle teaching was implemented in 30 primary school classrooms. Learning was assessed through pre- and post-intervention questionnaires. At the Obesity Pediatric Clinic, overweight and obese children of migrant background or low SES were trained on transcultural nutrition and invited to participate in the project. Primary school students increased their knowledge about healthy nutrition and the importance of physical activity (*p*-value < 0.001). At the Obesity Pediatric Clinic, after 6 months, pre–post-intervention variation in their consumption of vegetables and fruit was +14% (*p* < 0.0001) and no variation in physical activity habits occurred (*p* = 0.34). In this group, the BMI *z*-score was not significantly decreased (−0.17 ± 0.63, *p*= 0.15). This study demonstrates the feasibility and efficacy of telematic tools and targeted community approaches in improving students’ knowledge with regard to healthy lifestyle, particularly in schools in suburbs with a high density of migrants and SES families. Comprehensive and integrated approaches provided to the obese patients remain mostly ineffective.

## 1. Introduction

The prevalence of childhood overweight and obesity [1,2] as well as the consequent chronic diseases during adulthood are growing worldwide [3,4,5,6,7]. In Italy 20.4% of children aged 8–9 years are overweight and 9.4% are obese [8]; among adolescents aged 11–15 years, 16.6% are overweight and 3.2% are obese [9]. 

Low socioeconomic status (SES) and minority ethnic groups are disproportionately affected by overweight and obesity [10,11,12,13,14]. SES is strictly correlated with people’s level of physical activity (PA), sedentary behavior, and body mass index [15]. Children from low-SES families spend more time engaging in sedentary behavior and show a trend of higher BMI and lower PA levels than children from higher SES families [15]. 

After migrating to Western societies, families frequently abandon their traditional food habits to adopt Westernized dietary patterns containing higher levels of fat, sugar and salt. Accordingly, they often reduce their consumption of fruits and vegetables [16,17]. Moreover, the lack of a health-conscious exercise culture and the difficulties they encounter in accessing sport facilities in their new country represent another major predisposing factor to the onset or worsening of overweight and obesity; the sport activities offered often do not meet their traditions [14], are highly expensive, or difficult to find or to reach.

Recently, integrated and comprehensive approaches have been proposed in adults at different levels (individual, community, society) to overcome these barriers in low-SES and minority ethnic groups [18]:

(i) Individual level—there is evidence of the effectiveness of primary care-delivered tailored weight loss programs among deprived groups and an example is the *mosaic clinic*, a clinic specialized in ethnic-cultural diversity that provides individualized or group consultation with operators of the same ethnicity [19]. 

(ii) Community level—community-based behavioral weight loss interventions also have evidence of efficacy, at least in the short term. As an example, comprehensive school health (CSH) is an internationally recognized school-based health promotion approach that integrates multiple aspects to support students’ development as learners and as healthy and productive members of society. This approach has been shown to be effective in increasing levels of physical activity, improving dietary habits, and decreasing rates of obesity among children [20,21]. These results have also been confirmed in primary schools with a high proportion of migrant children [22,23]; among children of Mexican origin, a reduction in BMI growth was seen among obese boys in the intervention community (ß-coefficient = −1.94, *p* = 0.05) [23].

(iii) Society level—the Italian Society of Pediatrics recently proposed the diffusion of the “transcultural” food pyramid, which allows families of migrant background to rediscover their tastes and flavors in their diet in the context of nutritional balance proposed by the Mediterranean diet (Supplementary Materials Figure S1). 

Based on available evidence, we developed the “*Smuovi La Salute*” project, which is an integrated and comprehensive model of prevention and treatment of childhood overweight and obesity. This action–research project involves the population living in three different districts in the north of Italy and had three main aims:

- To spread knowledge on healthy lifestyles through a universal and community-targeted approach, offering tools to reduce socio-cultural and economic barriers;
- To educate children on healthy lifestyles and to measure the variation in the level of knowledge achieved in schools in suburbs with a high density of migrants and/or low-SES families;
- To prevent and treat overweight/obesity in primary care settings and treat obesity at the second and third levels of care through individual approach, with tools targeting subjects of low-SES or with a migrant background. In these groups, we evaluated lifestyle changes and variation in BMI z-scores.

## 2. Materials and Methods

The project “*Smuovi la Salute*” was planned by a scientific panel of professionals: pediatricians, experts in the fields of health promotion, physical education and nutrition, and cultural mediators. The project was targeted at three different levels (society, community, and individuals) and was implemented in three economically developed districts in the north of Italy (provinces of Bolzano, Trento and Verona), in suburbs with a high density of migrants and/or low-SES families. 

This action–research project lasted 30 months and included the following three phases:

- **2.1. Phase 1:** recruitment and training of operators (November 2017–May 2019);
- **2.2. Phase 2:** development of tools for healthy lifestyle promotion (May 2018–April 2019);
- **2.3. Phase 3:** enrolment of the target population and implementation of the project (November 2018–May 2020).

This project was funded by the Italian National Institute for Health, Migration and Poverty (INMP), a public entity under the Italian Ministry of Health and a Collaborating Center of WHO/Europe (https://www.inmp.it/ita/Archivio-Pubblicita-Legale/Archivio-Delibere/Delibere-2017/Delibere-2017-101-208, accessed on 13 October 2017).

### 2.1. Phase 1: Recruitment and Training of Operators

All the operators involved in the project were recruited and trained by highly specialized professionals who were part of the scientific panel:

- Family pediatricians and general practitioners followed a one-day course on healthy multi-ethnic nutrition from weaning to adulthood, respecting the cultural and religious needs of the children and their families;
- Cultural mediators of the four most represented ethnic groups in the three districts (Arab, Chinese, Urdu and Russian), students in nursing sciences from different countries and volunteers from foreign associations were trained during a 3-day course to provide advice and dietary recommendations according to the transcultural food pyramid;
- Cultural facilitators and sport operators of various disciplines followed, respectively, a 14 and 12 h course run by members of the scientific panel and experts in interculturalism to better understand the possible barriers to access to sport and recreational activities for immigrants, and thus facilitate their participation. After the course, sport activities in urban spaces (parks, green areas, squares) in some contexts with a high density of migrant residents were organized. The Italian Union Sport for all (UISP), a sport promotion body recognized by the Italian Ministry of Labor and Social operating in many Italian cities, was in charge of organizing these activities.

### 2.2. Phase 2: Development of Tools for Healthy Lifestyle Promotion

#### 2.2.1. Development of a Transcultural Healthy Eating App

A research foundation, Fondazione Bruno Kessler (FBK), in collaboration with the scientific panel of the project, developed the “*Smuovi la Salute*” app. The aim of this app was to help teenagers and their parents make correct and healthy food choices, from shopping at the supermarket to meal preparation. It guided families towards choosing the right food quality and quantity, suggesting the frequency of weekly consumption, thus trying to raise the awareness of parents and children regarding healthy eating through a process of “virtual coaching”. The app, free to download, was prescribed and distributed by health care professionals to families and followed the principles of the transcultural pyramid. Table 1 reports the main features of the app. 

The app was only developed in Italian to encourage integration into Italian communities, as suggested by the cultural mediators. Even in migrants, the level of literacy was appropriate and children helped their parents with the app.

#### 2.2.2. Development of a Cookbook Containing Healthy Multicultural Recipes

Through the direct involvement and participation of 67 persons coming from various foreign communities present in the area, recipes for healthy and simple dishes from different culinary traditions were collected. The scientific panel, with the collaboration of a nutrition specialist, evaluated and revised the recipes before publishing them in a book. The book “Healthy Cooking within everyone’s reach”, was presented through public seminars and events and disseminated through the Internet, social media, and the delivery of printed copies. The book is available online and in PDF format (Supplementary Materials Figure S2). The cookbook was only developed in Italian to encourage integration into Italian communities, as suggested by the cultural mediators.

#### 2.2.3. No-Cost Physical Activities in Urban Spaces

UISP organized no-cost physical and sport activities in urban spaces (parks and urban green spaces) in districts with a high density of migrant residents of the three provinces. UISP sport operators, previously trained, welcomed both patients with overweight and obesity problems sent by the Obesity Pediatric Clinic, and normal-weight children coming from low-SES families or from a migrant background. Children participating in the activities were 7–13 years old. Due to the difficulties in enrolling and keeping them regularly committed to the activities, sport activities were organized as after school activities for students, in suburbs with a high density of migrants and/or low-SES families.

#### 2.2.4. Nutritional and Video Laboratories at School

Nutritional laboratories were prepared and integrated during school time. These laboratories were led by pediatricians with the support of trained mediators and at least one teacher for each class. Children were placed in a “didactic atelier” setting where they could follow a frontal lesson on the basic principles of healthy nutrition and physical activity. They were involved in the discussion, raising doubts or questions, and worked together on nutritional exercises. The first part focused on some basic nutritional aspects such as the different nutrients, the food pyramid and how to eat a healthy breakfast, and on the benefits of practicing regular physical activity. The second part, which took place approximately one month later, consisted of a follow-up lesson on healthy nutrition and correct lifestyles. Moreover, students were encouraged to share what they had learned with their family, to offer them an opportunity to increase their knowledge and to motivate them to help their children improve their lifestyle. 

Due to the spread of the SARS-CoV-2 pandemic, all teaching materials realized for the nutritional laboratories were collected in video-laboratories, with the aim of providing teachers with an easy instrument to continue the activity during online teaching. The video format consisted of a cartoon, set in a typical day of school with a group of primary school students. The video is comprised of 8 clips, one for each different topic, which are linked to each other, but at the same time independent. Attached to each clip there is an information sheet addressed to teachers, containing some details on healthy nutrition and lifestyle, a summary of the topic and exercises for the students. All videos were previously tested with a control group of teachers and students receiving positive comments. All school leaders involved in the laboratories expressed interest in pursuing with the educational nutritional program. 

#### 2.2.5. Policy Environment

Brochures with the project details were prepared. They contained advice on healthy eating and were distributed to students, families, religious communities, associations, pediatricians, general practitioners, and other health professionals (Supplementary Materials Figure S3). 

### 2.3. Phase 3: Enrolment of the Target Population and Outcomes 

#### 2.3.1. Universal Approach

Meetings and conferences for the local community and for the different religious communities of the districts (Muslim, Chinese and Orthodox) were organized to present the tools developed to promote healthy transcultural nutrition and healthy lifestyle. Trained operators (including cultural mediators) were invited to moderate these meetings. The reason for needing to change behavior was presented as the necessity of preventing obesity, considered a disease associated with reduced self-esteem, depression, nocturnal sleep apnea, physical exercise intolerance, short-term orthopedic problems, as well as metabolic and cardiovascular complications that can be manifest since adolescence.

#### 2.3.2. Community Targeted

School teaching and learning was implemented in primary schools in the three districts, in suburbs with a high density of migrants and/or low-SES families. Primary school in Italy includes children and adolescents aged 6–14. In order to participate, primary schools did not have to be involved in a major health education diet/nutrition-related project. School administrators, school leaders and local health officials worked together to develop and implement the program. Thirty-two school classes were contacted, and 30 accepted to participate in the program. Students as well as families were invited to participate. All the students of the 30 classes were enrolled because the activity was proposed as part of the school program on the decision of the class council. 

Participants in the nutritional laboratories were asked anonymously to complete a pre- and a post-intervention questionnaire (after one month) in order to evaluate the learning development. Questionnaires, which differed according to age (6–10 and 11–14 years old), were developed by the Cà Foscari University of Venice, with the collaboration of professionals and academics working in the field of nutrition. Questionnaires included: 3 questions to check the children’s knowledge; 2 questions related to their eating habits and the level of physical activity practiced; 3 questions regarding the satisfaction about the activities proposed during the workshop, the cookbook, and the app (Table 2). 

In the questionnaires, students self-reported the number of times they achieved each goal, which referred to a modified behavior. A value of 1 indicated a change of behavior which became equal or greater than the recommended level; a value of 0 indicated a behavior which was lower than the recommended level; missing responses were excluded from the analysis. The number of behaviors that met the recommended level was calculated by adding the values with a maximum score of 6. The primary outcome of this intervention was to increase patients’/students’ awareness of the importance of eating five portions of fruit and vegetables daily and of implementing daily physical activity. Parents of the children enrolled in the laboratory received a brochure on the project and on healthy lifestyles, as well as a copy of the cooking book, and were encouraged to download the app.

#### 2.3.3. Individual Approach

- Trained family pediatricians and general practitioners implemented transcultural counselling in their routine activities and during their scheduled periodical free-of-charge physical examinations in both normal weight and overweight children (of 6, 9, and 13 years of age). Moreover, they gave information regarding the possibility of participating in no-cost physical activity in urban spaces organized by UISP, and to download the app and the transcultural recipe book.

- At the secondary and tertiary level Pediatric Obesity Clinic, subjects with the following inclusion criteria were enrolled:

(i) Age from 6 to 16 years;
(ii) BMI-for-age above 1 (overweight) or 2 (obese) standard deviation of the WHO growth reference median, respectively [24];
(iii) Migrant background and/or socioeconomic disadvantage. The family’s socioeconomic status (SES) was evaluated by ascertaining the mother’s educational level. Educational level was previously identified as an important indicator for SES [25] and was dichotomized into low- (≤14 years of education) and high- (>14 years of education) SES, which differentiates between families with a mother who has completed medium or higher education, college or university training, from other families [26].

Informed and privacy consent were obtained before participation in the study. All subjects were trained on transcultural nutrition by an expert dietician, invited to participate in no-cost physical activity in urban spaces organized by UISP and download the app, as well as received a printed copy of the recipe book.

Weight and height were measured during the recruitment and pre- and post-intervention questionnaires (at 0, 6 and 12 months) were administered to measure any change in nutritional habits and level of physical activity. These questionnaires were developed on the basis of the validated ones used by *Okkio alla Salute* (for 6–10-year-old children and their parents) [8] and in HBSC surveys (for 11–14-year-old youths) [9]. The main result of the intervention at this level was to evaluate the improvement in the daily consumption of vegetables and fruit (at least 3–5 daily portions) and in the increase in physical activity (at least 1 h per day for 5 days a week), as well as to highlight a possible BMI z-score variation. 

Statistical analysis was performed using SPSS Software version 25.0 (SPSS Inc., Chicago, IL, USA) and data were expressed in descriptive statistics. The independent *t*-test was used to compare the means of two independent groups. Statistical significance was considered at *p* < 0.05.

## 3. Results

### 3.1. Phase 1 

Trained operators included:

- 125 family pediatricians and general practitioners;
- 24 cultural mediators, nursing sciences students from different countries and volunteers from foreign associations;
- 42 cultural facilitators;
- 59 sport operators.

### 3.2. Phase 2

Healthy lifestyle tools, developed during the project, were disseminated as subsequently described. 

#### 3.2.1. Transcultural Healthy Eating App

The app was downloaded by 392 families (66% Italians, 34% migrants), and the data from the “satisfaction questionnaire” taken by 67 participants (60% Italians, 40% migrants) reported good scores: 4.15/5 on average.

#### 3.2.2. Cookbook with Healthy Multicultural Recipes

Twenty live book presentations were organized, 2396 paper copies were distributed and 1.481 people visualized the book online as a unique view.

#### 3.2.3. UISP Activities

A total of 11 activities in the three districts were organized by UISP and they were offered once a week; each activity lasted approximately 3 months. A total of 172 different children and adolescents participated in the activity. Each child participated in only one activity.

#### 3.2.4. Policy Environment

The project’s brochures were distributed to 884 families/community people, 398 pediatricians, 79 general practitioners, 173 other health professionals, 331 students, 225 members of associations, and 75 teachers.

### 3.3. Phase 3 

The target population was enrolled as subsequently described.

#### 3.3.1. Meetings with Communities on Healthy Lifestyles

Twelve meetings were organized by the scientific panel in collaboration with the trained operators. Meetings were addressed to the general population and were organized after press release, and after dissemination through community chat and brochures. Seventy persons attended the meetings.

Other five meetings were organized together with the religious communities, after preliminary meetings with their leaders; 23 persons attended these meetings. 

#### 3.3.2. Outpatient Clinic 

From November 2018 to April 2020, in the three Obesity Clinics, 97 patients accepted to be enrolled in the project: 83.5% were Italian of low SES, 4.1% were from eastern Europe, 5.1% were Urdu (Pakistan, India, Bangladesh), 2.1% were from South America, 2.1% were from Morocco, and 3.1% from other countries. Migrants of low SES represented 14.4%, and those of high SES represented 2.1%. Twenty-nine children (30%) attended the physical activity in urban spaces organized by UISP. Among these, 55% were Italian of low SES and 34% were from migrant and low-SES families. Only 30 among all the patients enrolled attended the clinic for a second appointment (+6–12 months) and completed the post-intervention questionnaire. Six out of thirty children enrolled by the obesity clinic also attended the physical activity in urban spaces organized by UISP.

Pre–post-intervention variation in the “self-reported” consumption of vegetables and fruit was +14% (*p* < 0.0001) and no variation in physical activity occurred in the cohort. BMI changed from 29.78 ± 6.56 to 27.38 ± 4.23 kg/m^2^, and the BMI z-score from 2.46 ± 0.43 to 2.28 ± 0.69 (*p* = 0.15). Complete data are reported in Table 3 and Table 4.

#### 3.3.3. UISP Activities

One hundred and seventy-two subjects were enrolled in the UISP physical activities. Only 15 children attended the activities after 3–12 months and completed the post-intervention questionnaire. 

#### 3.3.4. School Program

Six-hundred and ninety-five primary school students from 30 classes were recruited and completed the pre-intervention questionnaire; only 402 completed the post-questionnaires due to the closure of schools caused by the SARS-COV2 pandemic. 

Among the participants, 52% were female and 48% were male. The mean age was 9 ± 1.2 years. There was a 35% increase (from 69.3 to 93.5%) (*p*-value < 0.001) in nutritional knowledge on the correct daily frequency of vegetables and fruit consumption, and physical activity knowledge improved by 63% after the project (from 54.5 to 88.9%) (*p*-value < 0.001); the reported behavioral change regarding the daily frequency of vegetables and/or fruit consumption and physical activity improved from 36.6 to 51.7% (*p*-value < 0.001) and from 47.2 to 62.6% (*p*-value < 0.001), respectively. 

## 4. Discussion

In this study, we investigated the effects of the integrated and comprehensive project “*Smuovi la Salute*”, aiming to prevent and manage overweight and obesity among migrant children and/or children of low-SES. According to our knowledge, this was the first systematic approach targeted at this population reported in the literature.

Phases 1 and 2 were preparatory to develop the tools that could help reduce cultural and socioeconomic inequalities in the target population. In Phase 3, we tried to promote transcultural nutrition and healthy lifestyle at different levels. 

(i) At the societal level, we adopted a universal approach with the aim of not discriminating against children living in families experiencing socioeconomic deprivation and/or of migrant background. Telematic tools such as apps, online recipe books, and video conferences have been widely used and appreciated, as demonstrated by the number of download and views and by the satisfaction questionnaires. We developed the tools only in Italian language, to encourage integration, and data from the app download (66% Italians, 34% migrant family) reflect the percentage of population in our suburbs with a high density of migrants, confirming that language was not a barrier. In the literature, a few universal approaches aiming to reduce inequalities were reported, and studies have produced poor results [18]. The use of technology-based interventions, although not effective sustaining a reduction in weight, has been shown to be beneficial in short-term weight loss interventions [27,28], and according to our knowledge, this is the first project that produced interactive digital material on transcultural healthy lifestyles.

(ii) Regarding targeted community approaches, working with schools has been a promising strategy. In primary schools, we achieved an increased knowledge and reported change in behavior regarding healthy eating and physical activity. An “ad hoc” study with longitudinal follow up is needed to determine whether this improved knowledge can lead to long-term behavioral improvement. Our finding was also confirmed by a study set in Germany, of third and fourth grade classes with a high proportion of migrant schoolchildren [22]. Three months after the three-day practical nutrition lessons, the group had improved their knowledge, but without changes in nutrition-related behavior and attitudes. Two additional exercise lessons weekly positively affected parameters of fitness and motor skills after 6 months [29]. In boys of Mexican origin, curriculum-based interventions in the community and schools were effective in preventing BMI growth after 1 year [23].

On the other hand, a targeted community approach, culturally tailored towards ethnic communities, has not led to good results in our project. When we understood that in our territory the foreign communities do not represent structured realities with a referent to contact as a moderator to promote the events and the tools developed, we turned to religious leaders. However, the importance of promoting healthy transcultural nutrition was not a priority for religious groups. The involvement of cultural mediators could be another promising strategy, as reported by studies conducted with community-based groups conducting health education and counselling intervention among low-income women participants or with female majority [30,31]. These interventions have been shown to be effective in promoting weight loss at 4 months post-intervention, although this was not consistent over time. In our study, the cultural mediators we trained reported feeling more like translators than peers with a mission to improve lifestyle. Therefore, their effect was lower than expected.

(iii). With regard to the individual approach, the tools and activities offered by the program were meant to be used in primary care settings for overweight prevention, and on second and third level, as a support for obesity treatment. The majority of pediatricians were not effective in promoting and introducing young patients to UISP physical activities or in encouraging them to download the book’s PDF. Having a self-employed role, they probably had to be motivated with some form of incentive to optimize their project collaboration. In the literature, there is evidence of the efficacy in the short-term period (3 to 6 months) of primary care-delivered tailored weight loss programs among deprived groups [32]. Jeffery R.W. et al. demonstrated that after 1 year, their intervention prevented weight gain with age among high-income women; even if after 3 years, there was no significant effect on weight [33,34]. In our second- and third-level centers, the drop out at 6 months of follow up was 62%, and was different from the one of the Caucasian or non-disadvantaged obese population (28–37% of patients attending the three obesity clinic). A greater proportion of migrant children (9 out of 14) did the follow up compared to Italian children (21 of 81), probably because they perceived the project as inclusive. Due to the low number of patients enrolled for each ethnic group, we were unable to build peer groups to be supported by trained cultural mediators as reported for chronic diseases in a previous study [19]. Patients enrolled by the clinics, for 6–12 months, were able to increase their vegetable and fruit consumption (+14%) but not to improve their level of physical activity. In fact, we observed a high rate of drop out during the UISP activities. We identified several possible explanations of this behavior: providing no-cost activities helped in removing barriers and to facilitate the participation of low-SES families; however, on the other hand, participants did not feel the commitment and were often deciding not to come without any advice. In addition, participants were living in different parts of the city or different villages, and it was difficult to organize an activity in a place which was easily accessible for everyone. However, a greater proportion of low-SES migrant children attended the activity sessions compared with low-SES Italian children, confirming that activities organized for free, by previously trained sport operators, facilitated the participation of immigrants. 

Overweight children and adolescents from our cohort decreased their BMI z-score by −0.17 ± 0.63; however, this reduction was not significant, probably due to the low sample size of subjects that had at least two visits to the Pediatric Obesity Care (30 subjects), and the consequent impossibility to analyze larger data due to the drop out. However, the BMI z-score change we observed in our group was of the same magnitude as that of structured and intensive multidisciplinary group interventions [35]. In addition, it is slightly better than the mean change of −0.10 kg/m^2^ (95% CI −0.14 to −0.05) and −1.53 kg/m^2^ (95% CI −2.67 to −0.39) reported, respectively, in overweight or obese children aged 6–12 and 13–18 years of the last Cochrane review [36].

In addition to the concrete results of this project, we understand that our study has some limits: i. low-SES families were identified based on their educational level and not based on family income, which we could not evaluate; ii. the lack of randomization, due to the number of patients that accepted to be enrolled, did not allow us to obtain an adequate sample size to demonstrate a significant variation in lifestyle habits; iii. the follow up for the participants enrolled in schools was relatively short, mainly because of the interruptions of all the activities due to the SARS-COV2 pandemic; iv. due to the action–research design of the study, different populations and outcomes were analyzed, without a priori power size calculation; v. lifestyle behaviors were self-reported without other means to verify. However, we also believe that our study has big potential, supported by strong preliminary results, and can inspire future interventions: i. according to our knowledge, this is the first study reported in the literature regarding obesity clinic which has offered specific tools for obese patients with a migrant background or at a socioeconomic disadvantage. Migrant families appeared more engaged in physical activities and clinical follow up proposed by this project, than Italians of low SES. As reported in the literature, the treatment of childhood obesity remains largely ineffective. Most obesity programs are only successful in highly motivated families that are aware and willing to change their lifestyle and without severe psychosocial problems [37]; ii. the multidimensional approach of the project is, with the involvement of professionals in the health, telematic, social and sport fields, in our experience, a winning approach; iii the large pediatric population involved in the study; iv. the transferability of the tools developed from the clinical groups to the non-clinical population.

## 5. Conclusions

In conclusion, for the first time in the literature to the best of our knowledge, we reported the results of a comprehensive and integrated nutritional education project (*Smuovi la Salute*) for overweight and obese children of migrant background or at socioeconomic disadvantage. This study supports the importance of the use of telematics tools (an app, online transcultural recipe book, video-lessons and video-laboratory were developed and implemented) for promoting healthy lifestyles among the general population. Targeted community approaches in schools in suburbs with a high density of migrants, using a brief, low-intensity, nutrition education course as a method to improve students’ knowledge, and offering students after-school sport or physical activities, is feasible and effective. The video-laboratory has the advantage of being easily transferable to other schools. It would be interesting to also evaluate, in addition to the students’ nutritional education, the potential changes in their lifestyle and family habits. At the individual level, the comprehensive and integrated management of obese patients remains mostly ineffective; therefore, preventive measures are crucial and by using all the tools we developed, designing new tailored strategies of primary care engagement might help enhance the intervention.

## Figures and Tables

**Table 1:** Main features of the app “Smuovi la Salute”.

Main Features of the App “Smuovi la Salute”
Meal tracking through dialogue with the chatbot or with a food diary comprehensive of international foods and recipes
Tracking the achievement of the weekly goal, through messages of encouragement and reminder by the chatbot
Feedback on adherence to the transcultural pyramid through graphic reports and informative messages and suggestions
Request for nutritional information through free text dialogues
Gamification: game interface that develops step by step with the achievement of weekly goals
Periodic suggestions of multicultural recipes taken from the healthy recipe book
Final satisfaction questionnaire. A scale of values from 1 (very little satisfied) to 5 (very satisfied) was given by the subjects.

**Table 2:** Lists of questions used to check students’ knowledge and habits.

**Students Aged 6–10 Years**
How often should we eat fruit and/or vegetables?
How much movement should we do?
Check the images of the foods that fall into the vegetable group
How often do you eat fruit and/or vegetables?
How much movement do you do?
**Students aged 11–14 years**
Which of these represents a complete and balanced meal?
How often should we exercise to stay healthy?
How many servings of fruit and vegetables should be consumed each day?
How many times a week do you usually eat fruit and/or vegetables?
In the last 7 days, how many days have you been exercising (sports, games or activities that make your heart beat faster and can leave you breathless) for a total of at least 60 min a day?

**Table 3:** Anthropometric and lifestyle data of the overweight and obese subjects enrolled in the outpatient clinic.

Patients Enrolled at the Obesity Clinic	*n* = 97
Age (years)	10.7 ± 2.27
M/F (*n*)	56/41
M/F (%)	58/42
Italian of low SES	81 (83.5%)
Migrants of low SES	14 (14.4%)
Migrants of high SES	2 (2.1%)
Pre-pubertal/pubertal (*n*)	47/11
Pre-pubertal/pubertal (%)	48/52
BMI at baseline (kg/m^2^)	30.86 ± 4.16
BMI z-score at baseline	2.35 ± 0.69
Follow-up data	*n* = 30
Italian of low SES	21 (70%)
Migrants of low SES	9 (30%)
Follow-up duration (months)	8.40 ± 1.73
BMI at baseline (kg/m^2^) (*n* = 30)	29.78 ± 6.56
BMI z-score at baseline (*n* = 30)	2.46 ± 0.43
BMI at follow up (kg/m^2^) (*n* = 30)	27.38 ± 4.23
BMI z-score at follow up (*n* = 30)	2.28 ± 0.69

Data shown as the mean and standard deviation in brackets. Abbreviations: M, male; F, female; BMI, body mass index.

**Table 4:** Anthropometric and lifestyle variation from baseline to follow up of overweight and obese subjects enrolled in the outpatient clinic (*n* = 30).

Variation in BMI z-Score	−0.17 ± 0.63	*p*= 0.15
Variation in % of patients with physical activity at least 1 h per day for 5 days a week	0% (from 12.5 to 12.9%)	*p* = 0.34
Variation in % of patients with consumption of 3–5 daily portions of vegetables and fruit	14% (from 25.7 to 29.4%)	*p* < 0.0001

Data shown as the mean and standard deviation in brackets or as percentages. Abbreviations: BMI, body mass index.

## Data Availability

The data that support the findings of this study are not publicly available because they contain information that could compromise the privacy of research participants, but are available from the corresponding author upon reasonable request.

## Supplementary Materials

The following are available online at www.mdpi.com/article/10.3390/nu13103634/s1, Figure S1: the “transcultural” food pyramid; Figure S2: The cookbook: healthy cooking within everyone’s reach; Figure S3: The “Smuovi la Salute” brochure with the project details.
