# Supplementary material for: Educational Intervention of Healthy Life Promotion for Children with a Migrant Background or at Socioeconomic Disadvantage in the North of Italy: Efficacy of Telematic Tools in Improving Nutritional and Physical Activity Knowledge
Source: Nutrients. 2021 Oct 17;13(10):3634. doi: 10.3390/nu13103634 (PMC8540523; doi:10.3390/nu13103634)
Supplement: Supplementary file 1 [file nutrients-13-03634-s001.zip › Supplementary material Figure S1.pdf]

PIRAMIDE ALIMENTARE TRANSCULTURALE

Uno strumento di educazione alimentare per tutti i bambini sin dall'età prescolare.

Società Italiana di **Pediatria**

Come orientarsi nella scelta degli alimenti

LATTE: parzialmente scremato. YOGURT: parzialmente scremato bianco o alla frutta. CEREALI: preferire cereali integrali e riso parboiled (quest'ultimo massimo 2 volte alla settimana). Si raccomanda la cottura al dente. PANE: 1 panino al giorno, preferire quello preparato con farine integrali o con farina tipo 1. CARNE: pollo, coniglio, tacchino, vitello, manzo magro, maiale magro. PESCE: fresco o surgelato, preferire il pesce azzurro (sarde, alici). Merluzzo, nasello, sgombro, spigola o pesce persico meglio non più di 1 volta alla settimana. Cefalopodi (calamari, polpo) non più di 1 volta a settimana. Crostacei e molluschi bivalvi saltuariamente. Da evitare pesce di grossa taglia (pesce spada e tonno). LEGUMI: freschi, secchi o surgelati vanno sempre associati nello stesso pasto ai cereali (pasta, riso, orzo). Non sono verdure, ma una alternativa a carne, pesce, uova e formaggi. FRUTTA: 2 - 3 volte al giorno. Da preferire quella di stagione. Non frullata o passata. Da assumere non più di 2 o 3 volte alla settimana: kiwi o uva o banana o ananas o papaya o jackfruit. Da limitare (<1 volta alla settimana): plantano o datteri o avocado o tamarindo. VERDURA: fresca o surgelata, non frullata o passata, 2 volte al giorno. Da preferire verdura di stagione. CONDIMENTI: olio extra vergine di oliva. SALE: da limitare. COTTURA: in umido, al vapore, al forno, al cartoccio.

DOLCI-SNACK (consumo al minimo)

patatine fritte, platano fritto, cioccolato nachos, patate dolci stufate, frittelle di mele, dulce de leche, budino di riso, banane verdi stufate

CONDIMENTI (consumo al minimo)

utilizzo giornaliero dell'olio di oliva

UOVA, TUBERI E RADICI

1 – 2 porzioni settimanali

FORMAGGI

2 porzioni settimanali

CARNE

Max 3 porzioni settimanali. Pollo, tacchino, vitello, manzo magro, maiale magro, agnello, coniglio, anatra

PESCE

3 – 4 porzioni settimanali. Alici, sarde, calamari, merluzzo, gamberi

LEGUMI

4 – 5 porzioni settimanali. Fagioli, lenticchie, piselli, ceci, soia, fave

NOCI-SEMI

1 porzione al giorno

LATTE-YOGURT

1 – 2 porzioni al giorno

CEREALI

3 – 5 porzioni al giorno. Grano, mais, farro, orzo, sorgo, miglio, grano saraceno, quinoa, (preferire cereali integrali), riso parboiled

FRUTTA-ORTAGGI

3 – 5 porzioni al giorno (varietà di colori e di consistenza). Pomodori, melanzane, okra, carote, germogli di bamboo, squash, mele, arance, albicocche, guava, ciliegie, litchis, frutto della passione, mango, papaya

ACQUA

Assunzione giornaliera adeguata

ATTIVITA' FISICA-ADEGUATO RIPOSO

Convivialità, biodiversità e stagionalità, prodotti locali ed ecologici

TIPICITÀ

REGIONI AFRICANE

CEREALI: teff, sorgo

VERDURA E FRUTTA: okra, foglie di cassava, foglie del baobab, frutto della passione, frutto del baobab, guava, mango

REGIONI ASIATICHE

CEREALI: miglio, grano saraceno

VERDURA E FRUTTA: okra, germogli di bamboo, alghe marine, frutto del drago, litchis

REGIONI SUD AMERICA

CEREALI: quinoa, amaranto

VERDURA E FRUTTA: squash, okra, mango, fichi d'india, guava, melagrana

REGIONI MEDITERRANEE

CEREALI: grano duro (pasta, burghul, cous cous), grano tenero, farro

VERDURA E FRUTTA: pomodori, insalata, zucchine, mele, arance, albicocche

PER MANTENERSI IN FORMA SI CONSIGLIA DI:

Svolgere attività fisica quotidiana per almeno 1 ora. Alimentarsi in modo variato ed equilibrato. Mantenere l'abitudine di una prima colazione adeguata: è il carburante per la mattinata, l'attività scolastica e per il gioco. Evitare periodi di digiuno prolungati e distribuire ogni giorno gli alimenti in 4-5 pasti:

COLAZIONE: 15% delle calorie giornaliere.

La prima colazione può essere molto semplice ma deve essere completa:

- Latte o yogurt parzialmente scremato.
- Fette biscottate o pane integrale con marmellata o muesli o biscotti secchi o cereali per la prima colazione integrali.
- Frutta fresca o eventualmente spremuta di arancia.

SPUNTINO AL MATTINO: 5% delle calorie.

MERENDA A METÀ POMERIGGIO: 10% delle calorie.

La merenda di metà mattina e quella del pomeriggio sono momenti importanti della giornata perché servono a non lasciare un lungo intervallo tra i pasti, evitando di arrivare a pranzo o a cena affamati, con il rischio di mangiare troppo.

PRANZO: 40% delle calorie - CENA: 30% delle calorie.

Per assicurare un corretto apporto di tutti i nutrienti devono essere presenti carboidrati, proteine, lipidi e fibre. Due possono essere gli schemi da seguire:

- Piatto unico: primo piatto condito con carne o pesce o legumi o formaggio o uova + verdura + frutta.

- Primo piatto condito con verdura + secondo piatto (carne o pesce o legumi o formaggio o uova) + verdura + frutta.

Importante poi un'assunzione giornaliera adeguata di acqua!

LA DIETA MEDITERRANEA: VERSO UNA STRATEGIA NUTRIZIONALE E TRANSCULTURALE

La Dieta Mediterranea è caratterizzata da un modello nutrizionale costituito principalmente da assunzione appropriata di alimenti e nutrienti e stili di vita corretti:

- 5 pasti quotidiani (ad ogni pasto quantità moderate di cibi, possibilmente freschi e stagionali, dando importanza alla convivialità).
- Elevata assunzione di verdura, legumi, frutta, noci e cereali integrali.
- Consumo di pesce medio-alto.
- Elevata assunzione di acidi grassi insaturi (in particolar modo provenienti dall'olio di oliva).
- Basso intake di acidi grassi saturi.
- Medio-basso intake di prodotti caseari.
- Ridotta assunzione di carne, soprattutto rossa.
- Apporto moderato di sale.
- Stili di vita corretti fra i quali irrinunciabili l'esercizio fisico quotidiano.

Nella società multi-etnica presente in Italia l'aderenza alla dieta mediterranea è un importante strumento di prevenzione delle malattie croniche nell'adulto promuovendo l'integrazione di tradizioni alimentari diverse.

I 14 PASTI SETTIMANALI POSSONO ESSERE COSÌ SUDDIVISI:

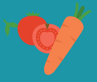
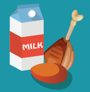
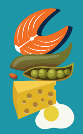

FRUTTA E VERDURA	3-5 porzioni al giorno
PASTA, RISO O ALTRI CEREALI	3-5 porzioni al giorno
TUBERI	1-2 volte la settimana (in sostituzione al primo piatto)
LATTE E YOGURT	1-2 porzioni al giorno
NOCI E SEMI	1 porzione al giorno (es 3 -4 noci)
CARNE	Max 3 volte la settimana (≤ 2 porzioni a settimana di carni bianche, < 2 porzioni di carni rosse, ≤ 1 porzione a settimana di carni lavorate es prosciutto cotto e bresaola)
PESCE	3-4 volte la settimana
LEGUMI	4-5 volte la settimana
FORMAGGI	2 volte la settimana
UOVA	1-2 volte la settimana

Società Italiana di Pediatria

Segreteria Società Italiana di Pediatria
Via L. Temolo 4 - 20126 - Milano
e-mail: segreteria@sip.it - www.sip.it

BioMedia La condivisione del sapere

PIRAMIDE ALIMENTARE TRANSCULTURALE

Uno strumento di educazione alimentare per tutti i bambini sin dall'età prescolare

L'alimentazione nel bambino è di fondamentale importanza per il suo sviluppo ed ha anche un grande valore in termini di prevenzione. La nutrizione, infatti, è uno dei principali fattori ambientali in grado di modulare il potenziale genetico e la crescita di ogni individuo.
L'organismo umano consuma continuamente energia, gli alimenti forniscono le sostanze necessarie per garantire il fabbisogno energetico e le necessità dei diversi organi.

COMITATO SCIENTIFICO:
Francesco Cataldo
Giovanni Corsello
Rosalia Maria Da Riol
Orazio Ranno
Maria Rosaria Sisto
Elvira Verduci

Società Italiana di Pediatria
