# Supplementary material for: Educational Intervention of Healthy Life Promotion for Children with a Migrant Background or at Socioeconomic Disadvantage in the North of Italy: Efficacy of Telematic Tools in Improving Nutritional and Physical Activity Knowledge
Source: Nutrients. 2021 Oct 17;13(10):3634. doi: 10.3390/nu13103634 (PMC8540523; doi:10.3390/nu13103634)

CUCINARE SANO alla PORTATA di TUTTI

RICETTE DA TUTTO IL MONDO

A cura di

Sara Carneri, Pirous Fateh-Moghadam (Dipartimento Salute e politiche sociali della Provincia autonoma di Trento).

Le ricette contenute in questo libro sono di Wassila Abassi, Patience Aiwerioba, Hanane Akarchaf, Redouane Belkouch, Fouzia Bendra, Alisha e Zainab Bhatti, Miriam Chamba, Shabnam Chhimpa, Veronica Ciubotaru, Emilia Csiki, Amal El Fathi, Pirous Fateh-Moghadam, Xueyan Fu, Michał Gołabek, Diana Gurgu, Mina Igli, Nadia Idri, Elsa Kadi, Meryem Koudiche, Viktoriia Kusturova, Yaquelin Labrade, Mack M'Baye, Safoora e Shoukat Mahmood, Eva Maigua, Jie Yang, Josiane Martin, Beenish Masood, Mariela Murillo, Imeri Nerminka, Magatte Niang, Aminata e Salamata Ouedraogo, Marija Petkovic, Mathurin Sikadi, Aligueton e Rasside Sogne, Fatoumata Togo, Aisha Traore, Halyna Vozna, Maddalena Wegher. Un ringraziamento a ciascuno di loro e a quanti hanno contribuito fornendo contatti, suggerimenti e qualche ricetta che si è fermata a metà.

Per gli approfondimenti si ringraziano: Beatrice Agostini (Oltre a mangiare sano è importante muoversi); Francesca Braito (Mentre il pane lievita c'è il tempo di costruire relazioni); Sara Filippi Plotegher (Tomatology, la strana storia del vegetale più famoso del mondo - testo e immagini); Monica Ghezzi (I bambini imparano a mangiare bene in famiglia); Rosa Maimone (Cambiare abitudini, a piccoli passi con Meddie e Cleo in una App); Paola Osti (L'olio extravergine di oliva e le sue innumerevoli proprietà; Ridurre sale e preparati pronti, scoprire spezie e aromi; I legumi sono gustosi alleati della nostra salute; Evitare i cibi spazzatura e le bevande zuccherate); Maddalena Wegher (Come usare la soia nella preparazione di alcuni piatti). Per il riesame delle ricette dal punto di vista nutrizionale e della salubrità si ringraziano Maria Grazia Zuccali, responsabile del Servizio igiene degli alimenti e della nutrizione (SIAN) dell'Azienda provinciale per i servizi sanitari di Trento, e Margherita Toschi, biologa nutrizionista dell'Azienda Ospedaliera Integrata di Verona.

Illustrazioni di Giorgia Molinari

Questo libro è stato realizzato nell'ambito del progetto "Percorso integrato di cura per la prevenzione e la gestione di sovrappeso e obesità in famiglie che appartengono a popolazioni migranti o in svantaggio socio-economico", denominato "Smuovi la salute". Il progetto è finanziato dall'INMP (Istituto Nazionale per la promozione della salute delle popolazioni Migranti e per il contrasto delle malattie della Povertà) e ha l'obiettivo di promuovere principi di sana alimentazione e sani stili di vita nella popolazione delle province di Trento, Bolzano e Verona. Gli enti partner del progetto sono: Azienda Provinciale per i Servizi Sanitari (APSS) Provincia Autonoma di Trento - S.C. Pediatria, Ospedale S. Chiara di Trento; Azienda Sanitaria dell'Alto Adige - U.O. di Pediatria, Ospedale di Bolzano; Azienda Ospedaliera Universitaria Integrata di Verona - U.O.C. di Pediatria; Dipartimento Salute e Politiche sociali della Provincia Autonoma di Trento; Fondazione Ca' Foscari di Venezia; Fondazione Bruno Kessler - High Impact Initiative (HII) Health & Well Being; UISP - Comitato del Trentino.

Per contatti e maggiori informazioni:
www.trentinosalute.net
mail: dip.salute@provincia.tn.it
facebook: @smuovilasalute
twitter: @Trentinosalute
Il libro è disponibile online su www.trentinosalute.net

cucinare sano
alla portata di tutti

ricette da tutto il mondo

Indice

Presentazione . . . 9
Ricette ispirate alla tradizione . . . 13
Come sono state raccolte le ricette . . . 15

Piatti unici . . . 17
Cous cous di verdure al vapore (Marocco) . . . 19
Cous cous di verdure al vapore con verdure (Algeria) . . . 21
Ceebujen (Senegal) . . . 23
Yassa di pesce (Senegal) . . . 24
Chicken pulao (Pakistan) . . . 26
Chicken rice (India - Rajasthan) . . . 27
Matar pulao (Pakistan) . . . 28
Ragù o goulash di soia (Italia) . . . 30
Riso e verdure al vapore (Marocco) . . . 32
Riso jollof con pesce (Nigeria) . . . 33
Trota al forno con grano saraceno (Polonia) . . . 35

Involtini e ripieni . . . 37
Byrek (Albania) . . . 39
Brik (Tunisia) . . . 41
Empanadas di formaggio (Ecuador) . . . 42
Peperoni ripieni (Albania) . . . 44
Golubtisi (Ucraina) . . . 45
Tamales con verdure e pollo (Colombia) . . . 47
Tortillas di patate (Ecuador) . . . 49
Vareniki con patate (Ucraina) . . . 50

Con le verdure . . . 51
Okra in umido (India - Rajasthan) . . . 53
Alcuni bambini mangiano verdure (Pakistan) . . . 55
Attiéké al vapore (Costa d'Avorio) . . . 56
Insalata di cipolle e pomodori (Ecuador) . . . 57
Insalata di rapanelli e uova sode (Moldavia) . . . 59
Gratin di patate (Martinica) . . . 60
Insalata di jarda (Marocco) . . . 61
Verdure saltate (Cina - Shandong) . . . 62
Tajine di verdure (Marocco) . . . 64
Verdure in umido (Macedonia) . . . 65

Con i legumi **67**
Crema di arachidi e erbette (Camerun). **69**
Crema di fagioli (Moldavia) **71**
Fagioli in umido (Nigeria) **72**
Frijoles (Colombia) **73**
Gnocchetti di fagioli dall'occhio (Burkina Faso) **75**

Con il pollo **77**
Picadillo di pollo (Cuba) **79**
Colombo de Poulet (Martinica). **81**
Ocra con pollo (Burkina Faso) **82**

Con il pesce **83**
Tajine di verdure e pesce (Marocco) **85**
Branzino al vapore (Cina - Gansu) **87**
Chevrette "Serge" (Martinica). **88**
Salmone al curry (Pakistan) **89**
Polpettine di sardine (Marocco) **91**

Zuppe e vellutate **93**
Borsh di fagioli (Ucraina). **95**
Harira (Marocco) **97**
Rosòł (Polonia) **98**
Zuppa di verdure (Transilvania) **99**
Scavleva zupa (Ucraina) **101**
Vellutata di ortiche (Moldavia) **102**
Zuppa di verdure e gamberetti (Mali). **103**
Zuppa di patate (Romania) **105**
Zuppa di pollo (Romania) **106**

Spuntini e merende **107**
Paté di melanzane (Moldavia) **109**
Aloo paratha (Pakistan) **111**
Arepas (Colombia). **112**
Mast-o-chiar (Iran). **113**
Marmellata di prugne (Moldavia) **115**
Baghrir (Algeria). **116**

Dolci e bevande **117**
Torta all'arancia (Italia) **119**
Gnocchi di patate e prugne (Transilvania) **121**

Budino di riso (Serbia) **123**
Succo di ananas, lime e zenzero (Martinica) **124**
Frullato di banana (Ecuador) **125**

Approfondimenti

Come cucinare il riso e altri piatti con la giusta quantità d’acqua **22**
Ridurre il consumo di carne fa bene alla salute e all’ambiente. **29**
Ridurre sale e preparati pronti, scoprire spezie e aromi **31**
Alcune regole per un’alimentazione sana **43**
Cambiare abitudini, a piccoli passi con Meddie e Cleo in una App . . **48**
L’olio extravergine di oliva e le sue innumerevoli proprietà. **54**
Modalità di cottura e preparazione dei piatti **63**
I legumi sono gustosi alleati della nostra salute **68**
Sprigionare le sostanze salutari contenute nell’aglio. **74**
Come usare la soia nella preparazione di alcuni piatti **80**
Conservare gli alimenti e ridurre gli sprechi **90**
I bambini imparano a mangiare bene in famiglia **104**
Mentre il pane lievita c’è il tempo di costruire relazioni. **110**
Oltre a mangiare sano è importante muoversi **114**
Scegliere farine integrali. **120**
Evitare i cibi spazzatura e le bevande zuccherate **122**
Tomatology, la strana storia del vegetale più famoso del mondo **126**

Ringraziamenti **133**

Presentazione

In ogni cultura l'arte culinaria occupa un posto centrale. Il modo in cui vengono trasformati gli alimenti in cibi elaborati ha molte similitudini e differenze nelle cucine di tutto il mondo e mangiare insieme, provare nuovi sapori, appropriarsi e modificare i piatti di altre culture, e della propria, fa parte dei più grandi piaceri della vita. È anche la prova che un'interazione positiva tra persone con abitudini diverse è possibile e piacevole, oltre ad essere fondamentale per il rinnovamento culturale necessario per non rimanere fermi nella tradizione. "Nella realtà nessuna cucina del mondo è immune da contatti e influenze con altre tradizioni", osserva Andrea Perin, "gli scambi fanno parte della storia e i sapori si sono sempre incrociati ed evoluti. Anzi, si può senza dubbio sostenere che maggiori sono gli scambi di un popolo e più ricca e piacevole risulta la sua cucina"[1]. Il premio Nobel Amartya Sen fa notare, ad esempio, che l'India non conosceva il peperoncino fino a quando i portoghesi non lo portarono dall'America. Oggi invece è presente, spesso in forma ustionante, nella cucina "tipica" indiana e fa parte di molte preparazioni di curry che, a sua volta, è un'invenzione inglese, sconosciuta in India prima di Lord Clive. Un discorso analogo si può fare a proposito di uno degli alimenti fondamentali della cucina italiana: il pomodoro, un immigrato che ha avuto davvero molto successo (come racconta l'estratto di Tomatology, a pagina 126). Il cibo non ha solo un valore nutrizionale: è legato alle memorie personali, alla storia universale, alla convivialità, evoca emozioni ed è uno degli aspetti che contribuisce, forse più di altri, a costituire la nostra identità.

Parlando di identità, spesso, la prima cosa a cui si pensa è la nazionalità o l'appartenenza religiosa ma ci sono aspetti altrettanto importanti che insieme contribuiscono a darci un volto: la lingua, il genere, l'orientamento sessuale e politico, la professione, lo stato civile, l'età e la classe sociale, le passioni musicali, letterarie e sportive, e le preferenze alimentari. Abbiamo tutti un'identità plurale che apre molte possibilità di incontro e rappresenta un antidoto contro la tendenza a creare linee di separazioni nette e impenetrabili tra un "noi" e un "loro". Secondo Amartya Sen[2] più si diffonderà questa consapevolezza, maggiore sarà anche la speranza di armonia nel mondo contemporaneo.

---

1. Andrea Perin, Ricette scorrette. Racconti e piatti di cucina meticcia, Elèuthera, 2009, p. 11
2. Amartya Sen, Identità e violenza, Laterza, 2008, p. 33

Un concetto legato a quello di identità è l'autenticità, che rappresenta spesso anche un criterio nella scelta di una ricetta o di un ristorante. In viaggio tutti cercano un locale "autentico" che proponga cibo genuino e nessuno vuole andare a mangiare in un posto "turistico". D'altra parte le tradizioni culinarie cambiano nel tempo, mentre il concetto di autenticità sembra implicare una prospettiva atemporale[3]. Come definire quindi l'autenticità? Non abbiamo una risposta a questa domanda, ma sappiamo che l'autenticità delle ricette che compongono questo libro non è dovuta alla fedeltà rispetto a una presunta ortodossia in termini di ingredienti o di preparazione. Le ricette di questo libro sono autentiche perché sono autentici i loro autori, persone che con molta generosità hanno condiviso la preparazione di alcuni piatti, così come vengono fatti normalmente a casa loro. Le ricette che vengono proposte si ispirano e traggono forza dalla tradizione del paese di origine, ma possono anche avere subito delle variazioni. Viceversa, ciascuno può scegliere di seguirle alla lettera, così come sono scritte, oppure sperimentare e apportare le proprie modifiche. Il risultato non sarà meno autentico. Con queste ricette potrete stupire i vostri ospiti con un'ottima tajine di verdure, anche se siete nati in Ucraina, e chi non è mai stato in Senegal riuscirà a preparare una gustosissima yassa di pesce. Le ricette contenute in questo libro hanno un importante valore aggiunto: il cibo che cucinerete non sarà solo buono ma farà anche bene alla salute.

La salute è infatti il filo rosso che lega le ricette del libro. È noto a tutti che l'alimentazione è legata strettamente alla salute: fin da piccoli, uno stile di vita sano può avere un impatto decisivo sulla salute e, nel corso della vita, chi mangia e cucina bene si mantiene in forma dal punto fisico, mentale e sociale. Un'alimentazione basata principalmente su alimenti di origine vegetale, con poche proteine animali, provenienti per lo più dal pesce (da pesca sostenibile), fa bene alla salute ed è essenziale per ridurre il riscaldamento globale e per reindirizzare il sistema alimentare affinché una popolazione mondiale di oltre 7 miliardi di persone possa nutrirsi senza oltrepassare i limiti della sostenibilità ambientale[4]. Per raggiungere questi traguardi di salute e di sostenibilità[5] occorre limitare il consumo

3. Arjun Appadurai, On Culinary Authencity, citato in: Marco d'Eramo, Il selfie del mondo, Feltrinelli 2017, p. 196
4. Come riferimento scientifico su questi temi si veda il rapporto della EAT-Lancet Commission "Healthy Diets From Sustainable Food Systems", pubblicato su The Lancet, vol. 393, 2 febbraio 2019, p. 447-492
5. Obiettivi importanti da raggiungere anche per la strategia per lo sviluppo sostenibile delle Nazioni Unite (www.unric.org/it/agenda-2030) recepite anche a livello italiano (http://asvis.it/agenda-2030)

di carne e incrementare il consumo di alimenti di origine vegetale, oltre a fare movimento e contrastare la sedentarietà. Questo vale in tutta Europa e anche in Italia, dove le percentuali di sovrappeso, obesità e malattie croniche prevenibili sono elevate. Evidenziare che questo cambiamento non è per forza collegato a una rinuncia, o alla necessità di aderire a qualche dieta avvilente e monotona, è uno degli obiettivi primari di questo libro. Nel tentativo di riuscirci abbiamo approfittato di una delle risorse più preziose che offre il nostro territorio: la presenza di persone provenienti da tutto il mondo.

In queste pagine si trovano ricette che prendono ispirazione dalla tradizione culinaria di 25 paesi diversi, elaborate con il coinvolgimento e la collaborazione di cittadini stranieri, o di italiani nati all'estero, residenti in provincia di Trento. L'auspicio è che il libro possa essere uno strumento utile per veicolare alcuni principi di sana alimentazione, favorire l'interazione tra persone di culture diverse e la scoperta di piatti da cucinare a casa, integrando il proprio menù grazie alla disponibilità di una grande varietà di cibi facili da preparare, basati su ingredienti freschi e salutari.

Sara Carneri e Pirous Fateh-Moghadam

Ricette
ispirate alla tradizione

Albania, Algeria, Burkina Faso, Camerun, Cina, Colombia, Costa d'Avorio, Cuba, Ecuador, India, Iran, Italia, Macedonia, Mali, Marocco, Martinica, Moldavia, Nigeria, Pakistan, Polonia, Romania, Senegal, Serbia, Tunisia e Ucraina sono i paesi di provenienza delle persone che vivono in provincia di Trento e hanno condiviso le loro ricette per farne uno strumento utile per la promozione della salute. Non è una food community ma tra loro c'è più di un cuoco e una cuoca, persone impegnate sul territorio in progetti culturali e sociali, genitori alle prese con bambini che scoprono sapori e gusti nuovi, persone che lavorano e quando tornano a casa si prendono il tempo di cucinare.

Sono 62 ricette, per lo più ispirate alla tradizione, con qualche variante legata alla fantasia e al gusto personale o alla necessità di sostituire alcuni ingredienti che non sempre sono reperibili. Ricette che mescolano memoria e creatività. "*Il procedimento è questo ma si può fare con le verdure che hai e puoi dosare le spezie in base al tuo gusto*". "*Questi involtini andrebbero fritti ma se li fai al forno sono buoni lo stesso*".

La maggior parte sono ricette vegetariane (41), di cui alcune vegane (26), con una varietà di tuberi, radici, legumi e verdure che hanno consistenza, colori e sapori diversi, da scegliere fresche e di stagione. Piatti con i cereali - riso, pasta, cous cous, farro, grano saraceno - e ancora uova, formaggi freschi e stagionati. Alcune ricette propongono preparazioni a base di pesce (12), un alimento salutare che andrebbe consumato almeno due volte a settimana. Poche le ricette con la carne (9), perlopiù pollo, ma è possibile sperimentare la stessa ricetta con tacchino e coniglio purché la carne non sia di allevamento intensivo, oppure provare a sostituire la carne con la soia disidratata (come viene suggerito a pagina 80). Dove possibile la carne è stata ridotta (andrebbe consumata non più di due volte a settimana) o viene proposta una variante della stessa ricetta altrettanto buona, mantenendo i riferimenti alla preparazione originale. In tutte le ricette c'è il sapore delle spezie - paprica, pepe, cumino, cannella, cardamomo, chiodi di garofano, coriandolo, curcuma, curry - e le erbe aromatiche: basilico, prezzemolo, origano, timo. Sono sapori che aiutano a ridurre sale, dadi e preparati pronti che è preferibile diminuire per stare in salute.

Sono ricette sane, non sempre facili e veloci: a volte la cottura è lenta ma "*mentre cuoce non devi mescolare di continuo, puoi fare qualcos'altro*". Sarebbe meglio avere la pentola giusta - vaporiera, couscoussiera, pentola a pressione, tajine, wok o una pentola larga con il fondo spesso - ma tutto o quasi si può fare con gli strumenti che abbiamo in cucina. In genere, come per tutte le ricette nuove, bisogna fare qualche prova prima di arrivare a un buon risultato. La cosa migliore è cucinare insieme a chi conosce la ricetta per imparare come si fa. Osservare, chiedere, farsi raccontare da chi ha esperienza di quel piatto. Questo piccolo libro è un invito a preparare cibi sani, aggiungere sedie, costruire relazioni. Un piccolo ricettario per integrare il menù con piatti delle diverse culture culinarie. È uno strumento utile anche a medici di base e pediatri per dare consigli di sana alimentazione tenendo conto delle diverse culture alimentari, dei gusti, dei sapori e delle abitudini delle famiglie.

Come sono state raccolte le ricette

Le ricette contenute in questo libro sono state raccolte tra maggio 2018 e gennaio 2019 in Trentino. A maggio è stato organizzato un incontro di presentazione del progetto “Smuovi la salute” insieme al Cinformi (Centro informativo per l’immigrazione della Provincia autonoma di Trento) a cui sono state invitate tutte le associazioni culturali delle comunità di immigrati in Trentino che promuovono la conoscenza della lingua, della cultura e delle tradizioni dei propri paesi di origine. In seguito, con i primi contatti sono stati organizzati numerosi incontri individuali per presentare il progetto, raccogliere le ricette di persona e, a volte, è stato possibile cucinarle insieme. A ciascuno abbiamo chiesto di indicarci qualcun altro da coinvolgere per ampliare i punti di vista, includere esperienze e ricette di diversi paesi. C’è chi vive in Trentino da molti anni, lavora, studia, sta cercando lavoro, ci sono famiglie con figli che frequentano la scuola, coppie giovani, persone che partecipano alle attività organizzate dalle associazioni sul territorio. Tutti hanno condiviso ricette dei loro paesi di origine accogliendo la proposta di un libro di cucina multiculturale con ricette sane per aiutarci a scegliere ingredienti freschi, verdure di stagione, ridurre i preparati pronti e il consumo di carne.

In questa ricerca e condivisione del progetto abbiamo incontrato alcune realtà che sul territorio portano avanti iniziative sociali e culturali e, attraverso il cibo, propongono occasioni di relazione, conoscenza e apprendimento reciproco. Contesti in cui le persone si conoscono, stanno insieme in modo informale, cucinano, fanno il pane, coltivano un orto e sperimentano. Sono esperienze radicate sul territorio che offrono contesti accoglienti, con cui fare rete per promuovere sani stili di vita e principi di sana alimentazione. All’interno del libro si trovano alcune testimonianze dei loro punti di vista. Non si tratta di una mappatura esaustiva di tutte le realtà e persone impegnate in questo senso poiché le associazioni e i progetti attivati sono molti. Un altro luogo in cui abbiamo incontrato persone e raccolto ricette è un ambulatorio pediatrico in una zona di Trento con alta presenza di immigrati. Un contesto che si è rivelato un osservatorio privilegiato per dialogare con le famiglie e scoprire nuove ricette.

In questo percorso abbiamo incontrato circa 50 persone provenienti da 25 paesi di diverse aree geografiche: America Latina, Asia, India, Europa

dell'Est, Africa settentrionale e subsahariana. Gli incontri si sono svolti concordando un luogo in cui parlare e confrontarsi in modo informale. Le ricette sono state poi riviste insieme per verificare ingredienti e quantità, procedimento e suggerimenti sulle modalità di cottura e possibili varianti. Le stesse ricette sono state riesaminate dal punto di vista nutrizionale e della salubrità da parte di esperti di nutrizione e sicurezza alimentare.

In genere quando raccontiamo una ricetta ne percepiamo il gusto, parlare di cibo riporta alla memoria le proprie radici, abitudini e tradizioni, mentre per le dosi ci regoliamo a occhio, facciamo come abbiamo sempre fatto. È difficile dare indicazioni precise poiché è nella pratica, mentre cuciniamo, che sappiamo dire come si fa. Molte ricette contenute in questo libro hanno come ingredienti principali le verdure, i cereali, i legumi, il pesce e attraverso l'esperienza è stata fornita indicazione delle quantità. Alcune riportano le dosi in grammi, altre fanno riferimento alla quantità contenuta in un bicchiere, una tazza o una ciotola. "I libri di cucina sono tutti un po' bugiardi" scrive Franco La Cecla, "perché cercano di sistematizzare una materia che è molto fluida, che si adatta a essere raccontata da persona a persona dinnanzi a un fornello e che nel momento in cui è scritta viene ridotta a una natura morta. [...] La differenza tra una cultura del cibo e un ricettario è la stessa differenza che c'è tra una lingua viva e una grammatica un po' noiosa"[1]. Un piccolo libro di ricette può essere uno strumento di scoperta, con alcune indicazioni su cosa mangiare per variare il proprio menù, oltre che un invito a cucinare insieme. In due o tre occasioni abbiamo cucinato e provato le ricette, per tutte le altre si tratta di trovare spazi di socialità in cui confrontarsi su come portare a tavola piatti sani e migliorare il proprio stile di vita.

Questo libro di cucina è uno degli strumenti realizzati nell'ambito del progetto "Smuovi la salute" insieme ad una App che, con un diario alimentare e quiz da risolvere, propone tutti i giorni delle sfide per migliorare il proprio stile di vita (realizzata da Fondazione Bruno Kessler), alle proposte formative sui principi di sana alimentazione per famiglie e bambini (promosse dalle unità di Pediatria dell'Ospedale S. Chiara di Trento, Ospedale di Bolzano, Azienda Ospedaliera Universitaria Integrata di Verona), ai corsi di attività fisica, gioco e movimento per bambini in palestra e all'aperto (organizzate dai comitati territoriali dell'Unione italiana sport per tutti di Trento, Bolzano e Verona).

Il progetto "Smuovi la salute" è stato realizzato grazie al finanziamento dell'INMP (Istituto Nazionale per la promozione della salute delle popolazioni Migranti e per il contrasto delle malattie della Povertà).

1. Franco La Cecla, La pasta e la pizza, Il Mulino, Bologna, citato in: Andrea Perin, Ricette scorrette. Racconti e piatti di cucina meticcia, Elèuthera, 2009, p. 19-20

Piatti unici

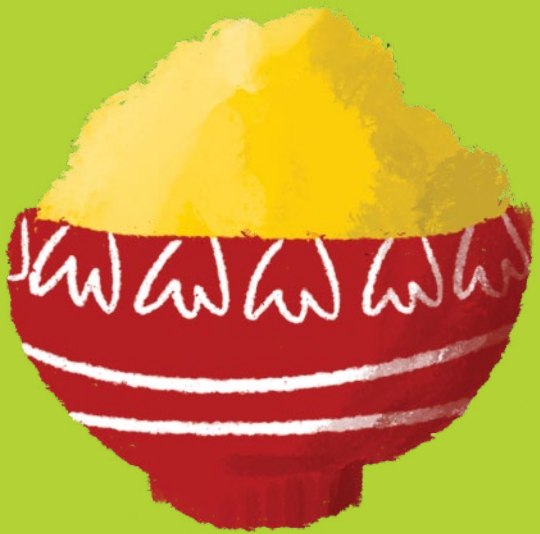

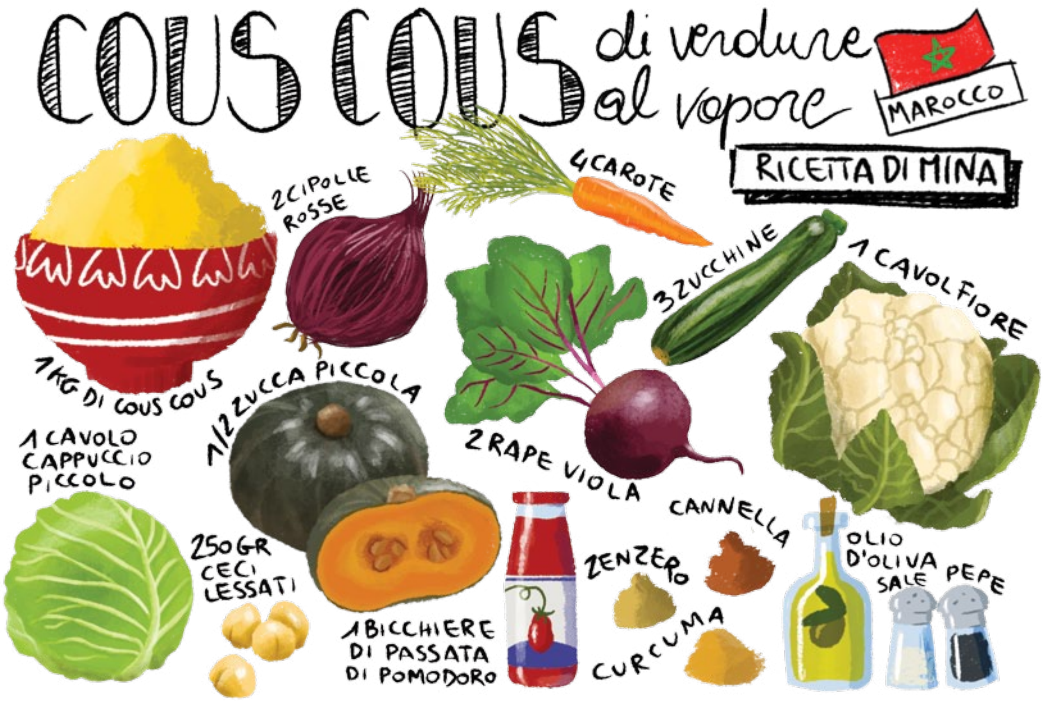

Ingredienti *(per 8 persone)*

1 kg di cous cous (medio)
2 cipolle rosse
4 carote
1 cavolfiore
1 cavolo cappuccio piccolo
½ zucca piccola (400 gr)
2 rape viola
3 zucchine
250 gr di ceci lessati
1 bicchiere di passata di pomodoro
curcuma in polvere
zenzero in polvere
cannella in polvere
olio d’oliva
pepe, sale

Preparazione

1. Lavate e pulite tutte le verdure. Tagliate le cipolle a fettine, le altre verdure a pezzi grossi: eliminate il torsolo del cavolfiore e staccate le cime; eliminate il gambo del cavolo cappuccio, tagliatelo a metà, poi in quarti; rape rosse, carote e zucchine a metà; pelate la zucca e tagliatela a pezzi grossi, lasciando un po’ di buccia.

2. Per la cottura a vapore si usa la couscoussiera (pentola a due piani per la cottura a vapore). Nella pentola sotto mettete 1 cucchiaio di olio d'oliva, cipolla, sale, pepe e le spezie. Fate rosolare, girando con un mestolo di legno, e unite la passata di pomodoro. Fate cuocere per qualche minuto a fiamma media, aggiungete le carote, 3-4 bicchieri di acqua bollente e proseguite la cottura.

3. A parte, in una ciotola larga e capiente, mettete il cous cous, un po' di olio d'oliva versato a filo e qualche goccia d'acqua. Lavorate il cous cous con le mani e sistematelo nella pentola con i buchi, da appoggiare sopra a quella delle verdure, senza mettere il coperchio. Lasciate cuocere per 15-20 minuti, assicurandovi che tra le due pentole non esca vapore (eventualmente sigillate con un canovaccio).

4. Aggiungete poi alle verdure: cavolo cappuccio, cavolfiore, rape, i ceci ben lavati e chiudete con il coperchio. Mentre le verdure cuociono, lavorare ancora il cous cous: mettetelo nella ciotola, aggiungete qualche goccia d'acqua e mescolate. Poi rimettetelo nella pentola con i buchi e fate cuocere ancora 15-20 minuti a vapore senza coperchio.

5. Infine aggiungete le ultime verdure (zucchine e zucca) e lavorate di nuovo il cous cous come prima, proseguendo la cottura a vapore per altri 15-20 minuti. Assaggiate, regolate di sale, e servite il cous cous in un piatto largo insieme al sugo e alle verdure.

Chi non è abituato a fare il cous cous può essere intimorito dalla cottura a vapore e dal ripetersi della lavorazione della semola. Il procedimento richiede un po' di tempo ma, a parte alcuni passaggi (preparare le verdure all'inizio, lavorare il cous cous tre volte), si tratta di aggiungere le verdure poco per volta in modo da rispettare i tempi di cottura. Non occorre sorvegliare di continuo la pentola mentre il cous cous cuoce. Le alternative (cuocendo le verdure in una pentola a fuoco medio e il cous cous, a parte, in una pentola antiaderente) vanno comunque bene.

COUS COUS al vapore con verdure

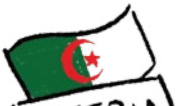

ALGERIA

RICETTA DI NADIA

Ingredienti *(per 4 persone)*

500 gr di cous cous
1 patata
2 zucchine
150 gr di piselli
1 cipolla ramata
2 carote
olio d'oliva
sale

Preparazione

1. Pulite e lavate tutte le verdure. Tagliate le cipolle sottili, le altre verdure a pezzetti piccoli tenendole separate.

2. Mettete il cous cous in una bacinella capiente, bagnatelo con un po' d'acqua, lavoratelo con le mani e fatelo riposare qualche minuto finché, assorbendo l'acqua, aumenta di volume. Nella couscoussiera (pentola a due piani per la cottura a vapore) mettete dell'acqua, nella parte sotto, e il cous cous sopra, nella pentola con i buchi. Fate cuocere a fiamma media per 15 minuti con il coperchio.

3. Togliete il cous cous dalla pentola, versatelo nella bacinella e lavoratelo di nuovo: aggiungete un po' di sale, qualche goccia d'acqua e olio d'oliva. Lavorate il cous cous con le mani e rimettetelo a cuocere nella pentola altri 15 minuti. Questo procedimento va ripetuto ancora una volta. Quando il cous cous è pronto tenetelo da parte, coperto con uno strofinaccio in modo che si mantenga caldo.

4. Controllate che nella pentola sia rimasta dell'acqua (se necessario aggiungetene un po') e portate a bollore. Fate cuocere le verdure a vapore nella pentola con i buchi a strati, in base ai tempi di cottura - carote, patate, zucchine, cipolla, piselli - con il coperchio.

5. Alla fine unite il cous cous alle verdure, aggiustate di sale e condite con olio d'oliva.

A differenza di altre ricette qui tutti gli ingredienti sono cotti a vapore e conditi con poco olio. Questo piatto può essere accompagnato con un bicchiere di kefir: un latte fermentato di consistenza simile allo yogurt, originario della regione del Caucaso che si trova nei negozi etnici o bio.

Come cucinare il riso e altri piatti con la giusta quantità d'acqua

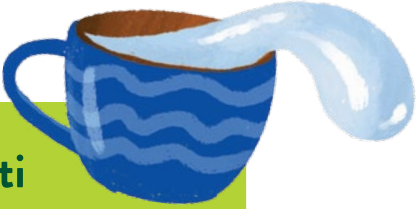

Le ricette che fanno parte della cultura culinaria di Pakistan, India, Senegal, Nigeria, Mali e altri paesi africani insegnano a dosare la quantità giusta d'acqua per cuocere il riso senza mescolare di continuo. Per il riso basmati, ad esempio, la proporzione suggerita è di 2 bicchieri di riso e 4 bicchieri e mezzo di acqua.

Per la preparazione potete fare così: sciacquate più volte il riso sotto l'acqua corrente finché l'acqua non rimane limpida, lasciatelo in ammollo per 30 minuti in acqua fredda e scolate.

Mettete il riso in una pentola, aggiungete 4 bicchieri d'acqua, il sale e portate a ebollizione. Fatelo cuocere finché il riso assorbe l'acqua, senza coperchio, a fiamma media. Aggiungete ancora mezzo bicchiere d'acqua e fatelo cuocere altri 5 minuti, coperto, a fiamma bassa. Quando i chicchi risultano sgranati il riso è pronto.

Usando riso integrale i tempi di cottura si allungano ma ci sono due vantaggi: è molto più salutare ed è praticamente impossibile scuocerlo.

In altre ricette l'acqua è una componente essenziale, come le minestre, le zuppe di verdura. Poca o molta dipende dai tempi di cottura e dalla consistenza che preferite.

Altre ricette prevedono la salsa di pomodoro in cottura o il latte, poca acqua e uno o due cucchiai d'olio d'oliva.

CEEBUJEN

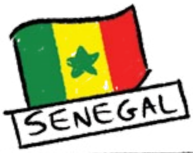

RICETTA DI MAGATTE

Ingredienti *(per 6/8 persone)*

500 gr di riso
1 kg di orata o pesce persico
(in tranci o intero da pulire)
1 cavolo
2 carote
2 patate medie
1 melanzana
1 cipolla
1 limone
1 peperoncino piccante
2 spicchi d'aglio
1 ciuffo di prezzemolo
passata di pomodoro q.b.
olio d'oliva
pepe, sale

Preparazione

1. Lavate e pulite tutte le verdure. Tagliate la cipolla sottile e le verdure a pezzetti non troppo piccoli.

2. In una pentola capiente mettete: olio, cipolla, aglio, peperoncino piccante e il pesce pulito e tagliato a pezzi grossi (in 4-5 parti, a seconda della grandezza). Fate rosolare qualche minuto il pesce su entrambi i lati, coprite con abbondante acqua e, poco alla volta, aggiungete tutte le verdure (cavolo, melanzane, carote, patate), la salsa di pomodoro, il sale.

3. Proseguite la cottura a fuoco medio, con il coperchio a trequarti. Quando verdure e pesce sono pronti, raccoglieteli con un mestolo forato e teneteli da parte, conservando il brodo di cottura che servirà per cuocere il riso. Se si è ristretto troppo aggiungete un po' d'acqua. Versate il riso nel brodo e fate cuocere per 20 minuti, a pentola coperta. Durante la cottura, finché c'è liquido, non mescolate. Si mescola soltanto alla fine, un paio di volte con un mestolo di legno, qualche minuto ancora e il riso è pronto. Servite il ceebujen come piatto unico con un po' di prezzemolo alla fine.

È un piatto tipico del Senegal che riunisce tutta la famiglia. Le dosi sono per 6/8 persone, se dovesse avanzarne è buono a cena, riscaldato, o il giorno dopo, conservandolo in frigorifero. In Africa il ceebujen si mangia tutti insieme, con il piatto di portata al centro, poiché il pranzo è un momento di incontro e relazione.

YASSA di PESCE

SENEGAL

RICETTA DI MACK

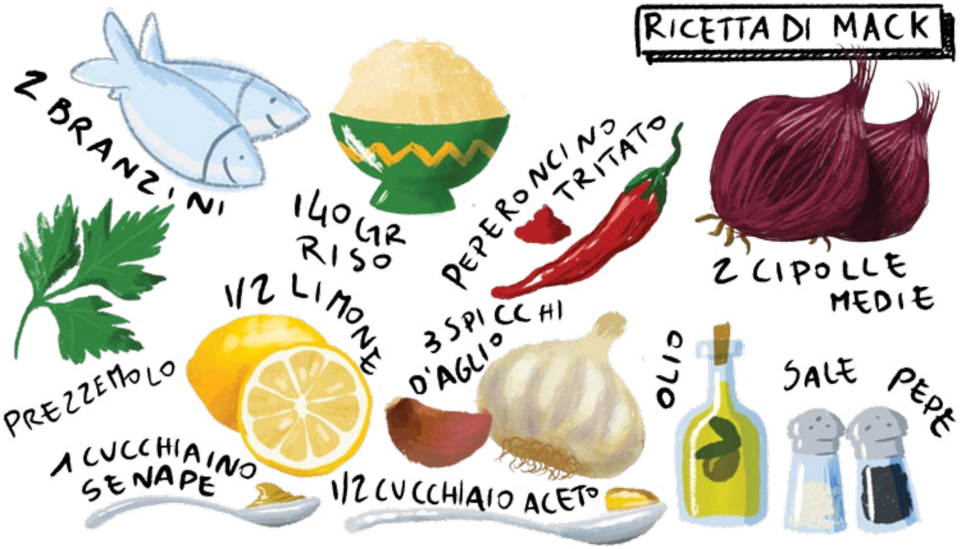

Ingredienti *(per 2 persone)*

2 branzini (orate o cernie) di medie dimensioni
140 gr di riso (basmati, jasmine o thai)
2 cipolle medie
1 ciuffo di prezzemolo
½ limone
3 spicchi d'aglio
1 cucchiaino di senape
½ cucchiaio di aceto bianco
peperoncino tritato
olio extravergine d'oliva
pepe, sale

Preparazione

1. Squamate perfettamente il pesce con un coltello. Tritate il prezzemolo e l'aglio e mettete tutto in una ciotola. Aggiungete sale, pepe e olio. Incidete trasversalmente il pesce (con 2 incisioni per lato) e, per ogni incisione, fate un buco con il dito. Inserite il trito di prezzemolo e aglio nella pancia del pesce e nelle incisioni laterali. Accendete il forno a 180°, prendete una teglia, foderatela con carta forno, appoggiate il pesce, aggiungete un cucchiaio d'olio e infornate per circa 20 minuti. Il tempo di cottura dipende dalle dimensioni del pesce.

2. Mentre il pesce cuoce, risciacquate il riso in acqua fredda per eliminare l'amido. Il riso sarà pronto per essere cotto quando l'acqua di risciacquo

sarà perfettamente pulita. In una pentola portate a bollore circa 300 gr d’acqua. Aggiungete il riso, mescolate bene, coprite con un coperchio e fate cuocere per 10-15 minuti a fiamma bassa. Al termine della cottura il riso avrà assorbito tutta l’acqua. Spegnete il fuoco e sgranate il riso con una forchetta.

3. A parte preparate la salsa: tagliate le cipolle a rondelle sottili e fatele saltare a fuco vivo in una padella per un paio di minuti con olio, sale, pepe, peperoncino e l’aglio tritato. Aggiungete senape e aceto bianco e mescolate in modo che la senape si sciolga e l’aceto evapori. Aggiungete il succo di limone, un goccio d’acqua e fate cuocere a fuoco basso finché la cipolla è appassita. Se serve, potete aggiungere un po’ d’acqua. A fine cottura le cipolle diventano una salsa morbida.

4. Servite il pesce con il riso bianco e la salsa di cipolle.

In Senegal, nelle case, la Yassa di pesce viene fatta con l’orata, mentre nei ristoranti si trova spesso con la cernia. In alternativa potete usare il branzino. La ricetta originale senegalese prevede che il pesce venga cotto alla griglia oppure fritto. Per praticità (rispetto alla griglia) e per rendere la ricetta più salutare (rispetto alla frittura) si può cucinare anche al forno. Per la salsa di cipolle, la ricetta originale prevede di aggiungere dado in abbondanza; senza è più salutare e il risultato è ugualmente buono. Se ne avanza, si può conservare in frigorifero per un giorno.

CHICKEN PULAO

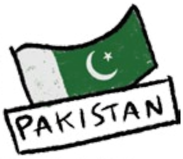

RICETTA DI BEENISH

Ingredienti *(per 4 persone)*

500 gr di pollo
500 gr di riso basmati
3 pomodori freschi
1 cipolla bianca
2 spicchi d'aglio
150 gr di yogurt bianco
mix di spezie *garam masala*
zenzero fresco
olio d'oliva
sale

Preparazione

1. Sciacquate il riso sotto l'acqua corrente. Lasciatelo in ammollo per 30 minuti in acqua fredda e scolate.

2. Pulite, lavate e tagliate a pezzi grossi il pollo (potete scegliere petto o cosce).

3. In una padella fate soffriggere la cipolla tagliata sottile con un cucchiaio d'olio. Quando è pronta, lasciatene metà da parte in una ciotolina. Unite alla cipolla rimasta nella pentola l'aglio e lo zenzero schiacciati, il pollo, il mix di spezie garam masala, lo yogurt e il pomodoro tagliato a pezzetti. Mescolate, fate cuocere per 10 minuti a fuoco medio con il coperchio, e aggiustate di sale.

4. Aggiungete il riso e 4 bicchieri di acqua. Fate andare a fiamma alta per 5 minuti poi continuate la cottura a fiamma bassa per 10 minuti con il coperchio. Controllate che il riso abbia asciugato l'acqua e assaggiate. Alla fine aggiungete il soffritto di cipolla che avete tenuto da parte e servite.

Questo piatto si trova nella cucina pakistana e indiana con alcune varianti. Le spezie che vengono usate fanno parte della tradizione di famiglia e fanno la differenza nei profumi e sapori che sprigionano. In genere la cucina pakistana è piuttosto piccante e speziata. Le spezie possono essere aggiunte direttamente nella pentola, con gli altri ingredienti, oppure si fa un sacchettino di stoffa in modo da tenerle unite e insaporire il brodo di cottura. Alcuni prodotti si trovano nei negozi etnici e nei reparti specializzati dei negozi o nei mercati contadini.

CHICKEN RICE

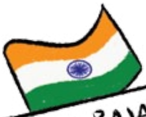

INDIA, RAJASTHAN

RICETTA DI SHABNAM

Ingredienti *(per 4 persone)*

500 gr di pollo
1 pomodoro maturo
2 cipolle
120 ml di yogurt bianco
3 tazze di riso basmati
4 tazze e ½ di acqua
cumino, semi e in polvere
coriandolo in polvere
curcuma in polvere
zenzero fresco
chiodi di garofano
pepe nero in grani
peperoncino piccante
1 spicchio d'aglio
olio d'oliva
sale

Preparazione

1. Pelate le cipolle e tagliatele sottili. Tagliate il pollo a pezzi di media grandezza. Lavate il riso basmati sotto l'acqua corrente un paio di volte, scolatelo e tenetelo da parte.

2. In una padella con il fondo spesso mettete un cucchiaio d'olio, la cipolla, semi di cumino, qualche chiodo di garofano e pepe nero. Fate rosolare per qualche minuto e aggiungete il pollo, un pizzico di sale e le spezie: curcuma, peperoncino piccante, coriandolo e cumino in polvere, uno spicchio d'aglio schiacciato e qualche pezzetto di zenzero. Fate cuocere per 5-10 minuti a fuoco medio.

3. Aggiungete il pomodoro fresco tagliato a pezzetti e, quando il pollo è morbido, yogurt bianco. Mescolate, continuate la cottura e aggiungete un po' di acqua calda. Quando riprende a bollire, unite il riso basmati. Fate cuocere per 10 minuti a fuoco basso, coprendo con un coperchio.

Questo piatto profumato si accompagna con la raita: una salsa di yogurt bianco fatta con una cipolla tritata, semi di cumino, una carota grattugiata, un pomodoro piccolo. È un piatto della cultura culinaria dell'India settentrionale, del Rajasthan, al confine con il Pakistan.

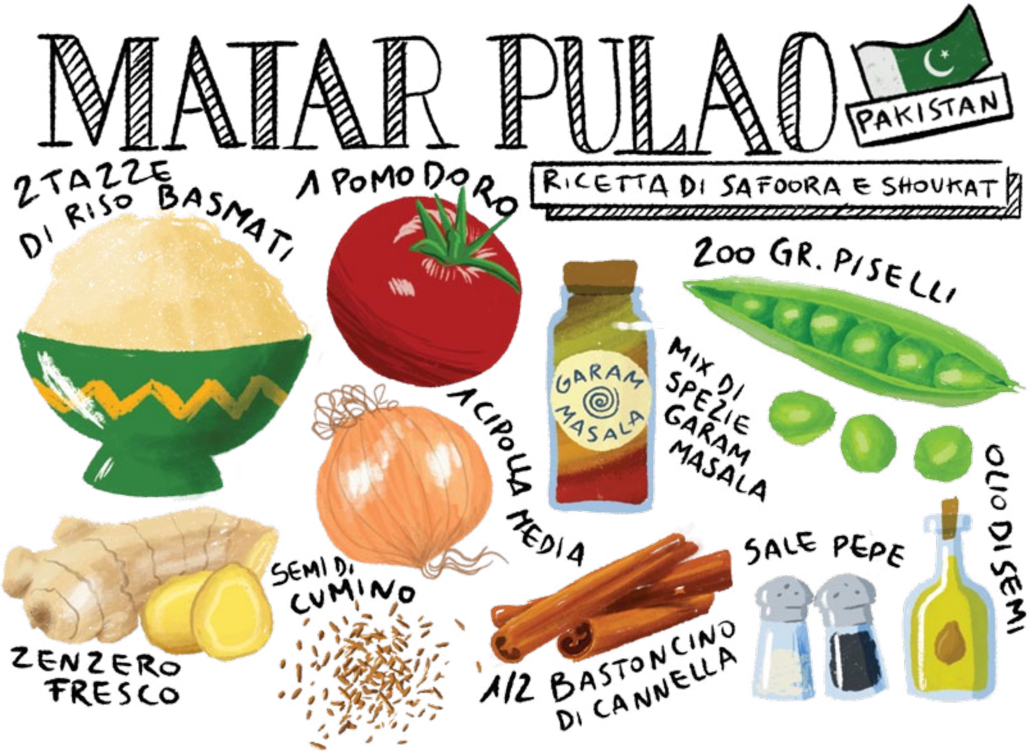

Ingredienti *(per 2 persone)*

2 tazze di riso basmati
1 pomodoro maturo
200 gr di piselli
1 cipolla media
zenzero fresco
semi di cumino
½ bastoncino di cannella
mix di spezie *garam masala*
olio di semi
pepe, sale

Preparazione

1. Sciacquate il riso basmati finché l’acqua risulta pulita, lasciandolo in ammollo 30 minuti. Scolate il riso e tenetelo da parte.

2. Pelate la cipolla e tagliatela sottile. In una padella, con un cucchiaio d’olio, fate rosolare la cipolla con un pizzico di cumino, un pezzetto di zenzero fresco tritato, un pezzettino di cannella e le spezie *garam masala*. Aggiungete i piselli, il pomodoro tagliato a pezzetti piccoli, un pizzico di sale, qualche cucchiaio d’acqua, mescolate e fate cuocere 10 minuti.

3. Aggiungete il riso basmati alle verdure e 4 tazze d’acqua calda. Fate cuocere per 5 minuti senza coperchio, a fiamma media, in modo che il

riso assorba l'acqua. Aggiungete ancora ½ tazza d'acqua e fate cuocere altri 10 minuti con il coperchio a fiamma bassa. Il piatto è pronto quando i chicchi di riso risultano ben sgranati.

Questo piatto della cultura culinaria pakistana è semplice e si prepara di frequente, con poche verdure, spezie e riso. La pentola in cui viene preparato ha un fondo spesso e gli ingredienti non si attaccano. Se scegliete riso basmati integrale i tempi di cottura si allungano di poco e beneficiate di tutti i principi nutritivi.

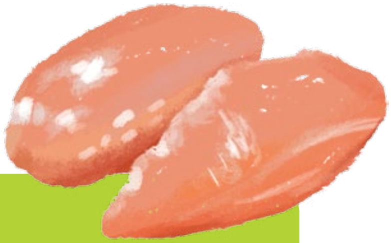

Ridurre il consumo di carne fa bene alla salute e all'ambiente

Secondo la EAT-Lancet Commission (2019), una dieta basata quasi esclusivamente su alimenti vegetali (verdura, frutta, legumi, semi oleosi, cereali integrali, oli vegetali), con pochi cibi di origine animale, senza merendine, snack o bibite, avrebbe notevoli vantaggi per la salute.
Questo stile alimentare riduce la probabilità di contrarre malattie croniche (malattie cardiovascolari, diabete e alcuni tumori) e, se tutti la seguissero, si eviterebbero circa 11 milioni di morti all'anno nel mondo (tra il 19% e 24% delle morti annue totali tra gli adulti). Nel 2015 la IARC (Agenzia internazionale per la ricerca sul cancro), dopo aver esaminato oltre 800 studi scientifici, ha stabilito che il consumo di carne lavorata (salumi e insaccati) e carne rossa può provocare il cancro (soprattutto quello al colon retto).
Inoltre, la produzione industriale di carne è uno dei maggiori responsabili del riscaldamento globale, al pari del traffico delle auto, oltre a consumare una quantità elevata di acqua (1 kg di carne bovina consuma 15 mila litri di acqua, mentre per 1 kg di ortaggi il consumo è di 100 litri).
La questione della produzione industriale di carne riguarda, oltre agli animali, anche le persone che lavorano nei macelli: un lavoro spesso pagato male, alienante e pericoloso.

RAGÙ o goulash di soia

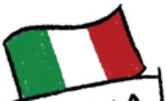

ITALIA

RICETTA DI MADDALENA

Ingredienti *(per 2 persone)*

100 gr granulato di soia disidratata
1 bicchiere di passata di pomodoro
½ l di brodo vegetale
1 gambo di sedano
1 carota
1 zucchina
1 cipolla
1 spicchio d'aglio
peperoncino piccante
rosmarino
olio d'oliva

Preparazione

1. Versate il brodo vegetale, leggermente salato, in una pentola e unite, a freddo, la soia disidrata. Portate a bollore, fate cuocere per 5 minuti, spegnete il fuoco e lasciate riposare finché non si raffredda, con il coperchio. Se seguite questo procedimento la sera prima, potete lasciare la soia nel brodo tutta la notte, così si insaporisce meglio. Per regolarvi sulle quantità, considerate che la soia dev'essere immersa nel brodo, in modo da assorbire il liquido.

2. Pulite e lavate tutte le verdure, tagliatele a pezzettini piccoli e regolari. In una pentola capiente mettete 2 cucchiai d'olio, aggiungete sedano, carota, zucchina, cipolla, aglio (precedentemente tagliati) e fate rosolare a fiamma vivace per qualche minuto.

3. Scolate la soia dal brodo, senza strizzarla, e unitela al soffritto di verdure. Aggiungete le erbe aromatiche che preferite (rosmarino, alloro o prezzemolo), peperoncino e altre spezie, in base ai vostri gusti. Aggiungete la passata di pomodoro e mescolate in modo da amalgamare gli ingredienti.

4. Fate cuocere a fuoco lento finché le verdure sono cotte e il ragù raggiunge la consistenza che desiderate. Se lasciate riposare il ragù fino al giorno dopo, in frigorifero, è più buono e saporito.

In modo analogo potete preparare il goulash piccante all'ungherese, scegliendo bocconcini di soia anziché granulato, con qualche variante per le verdure. Per il goulash potete abbondare con la cipolla, aggiungere patate e peperoni a pezzetti non troppo piccoli, erbe aromatiche, paprika dolce e piccante. Tra le varietà di erbe aromatiche, per dare sapore a queste preparazioni potreste aggiungere alloro, finocchietto selvatico, timo e salvia. Sono quattro delle undici erbe aromatiche che Maddalena raccoglie e usa nelle sue ricette. Per le spezie provate con qualche bacca di ginepro, chiodi di garofano, un pezzetto di cannella, radice di curcuma, macis e noce moscata in polvere.

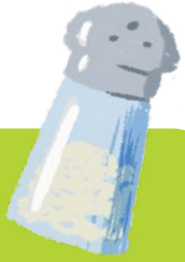

Ridurre sale e preparati pronti, scoprire spezie e aromi

L'OMS (Organizzazione mondiale della sanità) raccomanda di non consumare più di 5 g al giorno di sale (circa un cucchiaino raso di sale fino), ma in Italia gli adulti ne consumano mediamente il doppio.
Il sale viene usato per dare sapore e consistenza alle preparazioni oltre che per conservare gli alimenti, ed è contenuto in moltissimi prodotti industriali (compresi i prodotti da forno e i dolciumi).
Il consumo eccessivo di sale è responsabile dello sviluppo di ipertensione e malattie cardiovascolari, tumori, osteoporosi e malattie renali (Centro nazionale per la prevenzione e il controllo delle malattie).
Come fare per ridurre il consumo di sale e guadagnare salute? È consigliabile leggere le etichette e scegliere prodotti confezionati con minor contenuto di sale; limitare l'uso di condimenti (dadi, salsa di soia, senape) e salumi; non mettere in tavola il sale per evitare la tentazione di aggiungerne; scegliere verdure e legumi freschi anziché conservati.
Dalle culture culinarie di altri paesi possiamo imparare a usare le spezie (cannella, coriandolo, cumino, curcuma, curry, noce moscata, paprika, pepe, peperoncino, zafferano), che siano semi, polvere, radici, foglie fresche.
In cucina, oltre alle erbe aromatiche (basilico, prezzemolo, rosmarino, salvia, menta, origano, maggiorana, timo, semi di finocchio) anche limone, aceto, aglio, cipolla, sedano, porro, olive aiutano a ridurre il sale poiché durante la cottura danno sapore ai piatti.

RISO e VERDURE al vapore

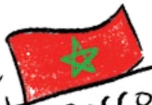

MAROCCO

RICETTA DI MERYEM

Ingredienti *(per 4 persone)*

3 bicchieri di riso
1 bicchiere di ceci lessati
400 gr di coste o bietole
1 cipolla media
1 cucchiaio di concentrato di pomodoro
1 ciuffo di prezzemolo
1 cucchiaio di olio d'oliva
pepe, sale

Preparazione

1. Lavate e pulite tutte le verdure. Tagliate la cipolla e le coste a pezzetti, tritate il prezzemolo. Sciacquate il riso sotto l'acqua corrente.

2. Per la preparazione di questo piatto si usa la pentola a vapore. Mettete l'acqua nella pentola (sotto), le coste e le cipolle nella pentola con i buchi (sopra) insieme al riso, un cucchiaio di concentrato di pomodoro, sale, pepe e olio d'oliva. Mescolate e fate cuocere a vapore, con il coperchio, per 15-20 minuti. Verso fine cottura aggiungete i ceci lessati e mescolate in modo che gli ingredienti si insaporiscano. A fine cottura aggiungete il prezzemolo e regolate di sale.

Nella cucina marocchina ci sono molti piatti di verdure e riso a cui in genere si aggiunge carne di vitello o agnello. Non sempre si usa la tajine, spesso la cottura è a vapore, nella couscoussiera. Per questa ricetta, oltre ai ceci e alle coste potete aggiungere piselli, patate, carote e altre verdure di stagione, facendo attenzione ai tempi di cottura. La combinazione di ceci e coste è molto gustosa.

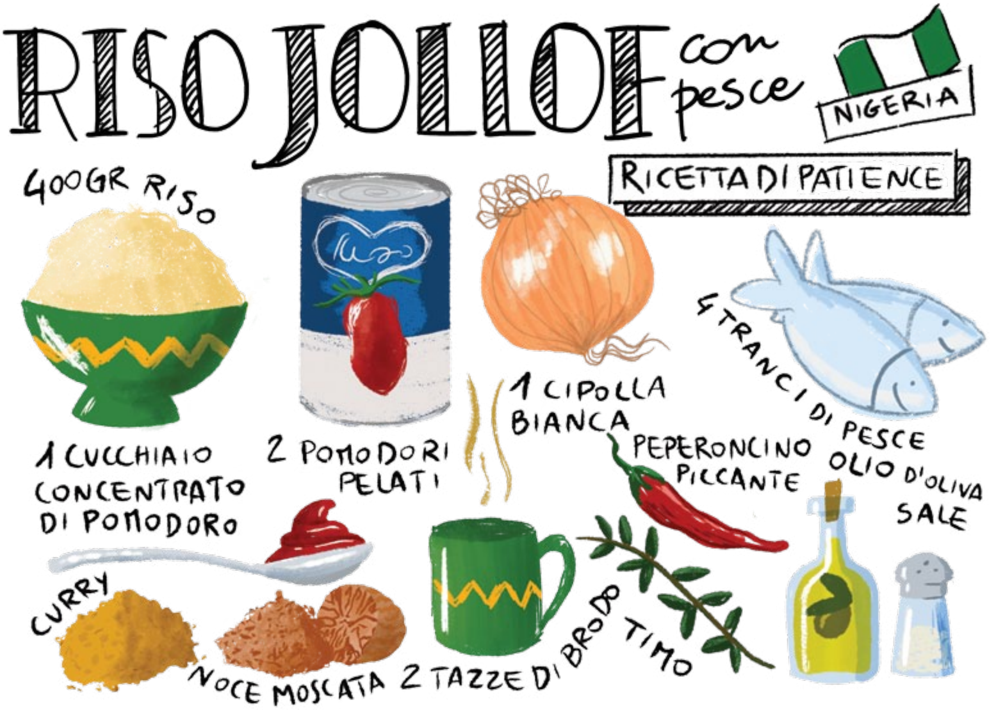

Ingredienti *(per 4 persone)*

400 gr di riso
2 pomodori pelati
1 cipolla bianca
4 tranci di pesce
(orata, sgombro o salmone)
1 cucchiaio di concentrato
di pomodoro
2 tazze di brodo vegetale
peperoncino piccante
noce moscata
timo
curry
olio d'oliva
sale

Preparazione

1. Lavate il riso sotto l'acqua corrente, fatelo bollire in abbondante acqua per 5 minuti e scolatelo. A parte, in un pentolino, preparate un brodo di verdura leggero: con ½ cipolla, un gambo di sedano, una carota, acqua (oppure potete usare dado vegetale).

2. In una padella capiente fate soffriggere la cipolla tagliata sottile con un cucchiaio d'olio. Aggiungete peperoncino, concentrato di pomodoro, pomodori pelati e cuocete a fiamma bassa. Insaporite con timo, curry,

noce moscata, sale e continuate la cottura. Quando la salsa è quasi pronta unite il riso, il brodo vegetale e mescolate. Fate cuocere ancora 10-15 minuti a fiamma bassa.

3. Nel frattempo lavate bene i tranci di pesce, asciugateli con un foglio di carta assorbente, e fateli cuocere a fuoco vivace in una padella antiaderente con due cucchiai d'olio e poca acqua, girandoli su entrambi i lati. Coprite la pentola e fate cuocere 10 minuti.

4. Quando il riso è pronto, unite il pesce e portate a tavola.

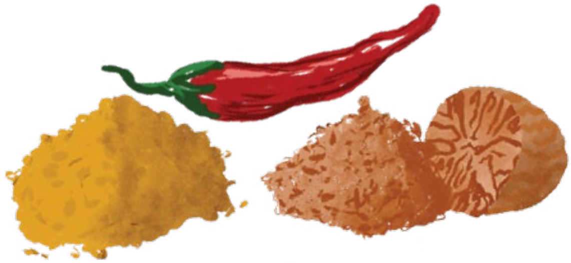

Il riso al pomodoro è uno dei piatti tipici della cucina nigeriana. Si fa con il pesce oppure con carne di manzo o pecora o pollo. Il riso viene aggiunto alla salsa con la quantità d'acqua (o brodo) giusta, non va mescolato spesso ma una o due volte all'inizio, poi cuoce lentamente. In alternativa si può cuocere il riso a parte e unirlo alla salsa alla fine.

TROTA al FORNO con grano saraceno

POLONIA

RICETTA DI MICHAŁ

Ingredienti *(per 2 persone)*

140 gr di grano saraceno
2 trote
2 manciate di spinaci crudi
1 spicchio d'aglio
olio d'oliva
limone
pepe, sale

Preparazione

1. Sciacquate le trote sotto l'acqua corrente e asciugatele con un foglio di carta assorbente. Farcite la pancia di ciascuna trota con ½ spicchio d'aglio, un pugno di spinaci, un cucchiaino d'olio, pepe e sale. Prendete una teglia e foderatela con carta da forno. Appoggiate le trote nella teglia e disponete qualche fetta di limone sul dorso. Infornate a 200° per 30 minuti circa.

2. Nel frattempo, in una pentola capiente fate bollire dell'acqua, con poco sale, aggiungete il grano saraceno e fatelo cuocere per 20 minuti. Quando è pronto, scolatelo e tenetelo da parte.

3. Servite le trote insieme al grano saraceno condito con un cucchiaio d'olio.

Questo piatto combina alimenti e cotture sane e veloci. Fa parte della tradizione culinaria polacca e propone il grano saraceno al posto del riso o di altri cereali. Nella cucina polacca ci sono molte ricette con il pesce, specialmente carpa e trota, mentre il grano saraceno viene usato anche come ripieno nella preparazione di alcuni piatti.

Involtini e ripieni

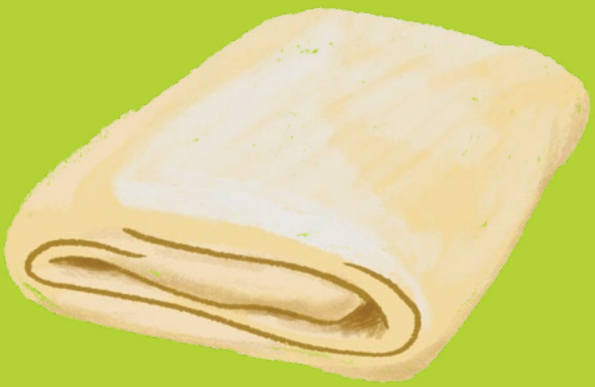

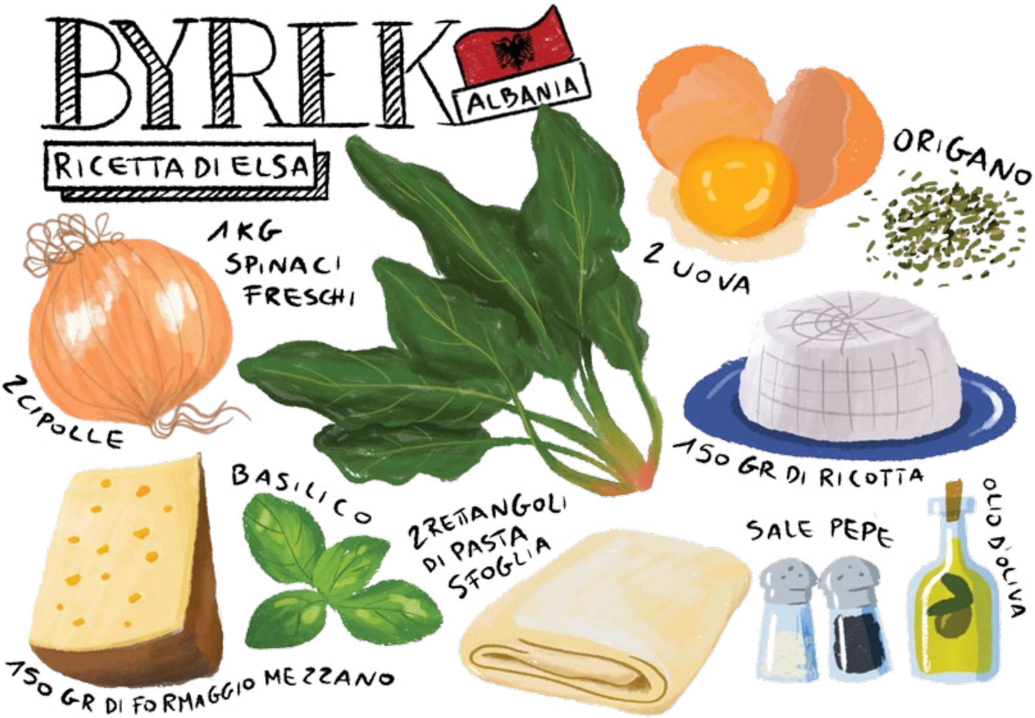

Ingredienti *(per 5 persone)*

1 kg di spinaci freschi
2 cipolle medie
150 gr di formaggio mezzano
150 gr di ricotta
2 uova
2 rettangoli di pasta sfoglia
olio d'oliva
origano
basilico
pepe, sale

Preparazione

1. Lavate e pulite le verdure. Tagliate le cipolle sottili, gli spinaci a pezzetti grossi.

2. In una padella capiente fate soffriggere la cipolla con un cucchiaio d'olio d'oliva e mescolate. Quando la cipolla è imbiondita aggiungete gli spinaci e fate cuocere a fiamma media, con un po' d'acqua per non farli attaccare. Quando sono pronti fateli raffreddare.

3. A parte, in una ciotola, unite le uova, la ricotta, il formaggio a pezzetti. Mescolate e aggiungete origano, basilico, sale, pepe. Unite gli spinaci amalgamando bene.

4. Nel frattempo, preparate una teglia con un cucchiaio d'olio, ungete il fondo e infarinatela. Appoggiate la pasta sfoglia nella teglia, fatela aderire ai bordi e punzecchiatela con una forchetta. Versate l'impasto nella teglia distribuendolo bene. Coprite con un'altra sfoglia e chiudete i bordi ripiegandoli all'interno. Infornate a 180° per 30 minuti.

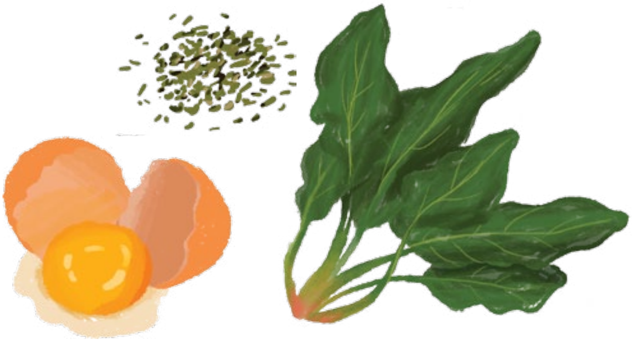

Il byrek è un piatto tipico della cucina albanese e turca. L'impasto può essere di carne o di verdure. In Albania si trova con la stessa facilità della pizza. Se viene preparato in casa anche la sfoglia è fatta a mano e somiglia alla pasta fillo. La ricetta tradizionale prevede più strati di sfoglia tirata molto sottile.

BRIK

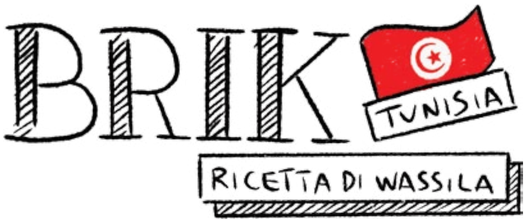

TUNISIA

RICETTA DI WASSILA

Ingredienti *(per 6 persone)*

100 gr di tonno in scatola
1 cipolla media
4 uova
100 gr di formaggio grana
1 ciuffo di prezzemolo
10 sfoglie di pasta brik

Preparazione

1. Pulite e tritate la cipolla a pezzetti piccoli, il prezzemolo tagliato fine.

2. In una ciotola unite il tonno, fatto sgocciolare e sminuzzato, il formaggio grattugiato, le uova, la cipolla, il prezzemolo, sale, pepe e mescolate bene tutti gli ingredienti.

3. Prendete le sfoglie di pasta brik e tagliatele a metà. Una alla volta, prendete una sfoglia e farcitela con un cucchiaio o due di impasto al centro. Arrotolate la sfoglia come un involtino e chiudete bene i lati in modo che il ripieno non esca durante la cottura. Procedete allo stesso modo con le altre sfoglie.

4. In una teglia, con un cucchiaio d'olio sul fondo, appoggiate gli involtini e infornate a 180° per 20 minuti.

Nella ricetta tradizionale i brik vengono fritti, ma la cottura al forno è un'alternativa più sana. A questo impasto potete aggiungere altre spezie, in base ai vostri gusti, e qualche patata lessa schiacciata. La pasta brik si trova nei negozi etnici, è sottilissima e somiglia alla pasta fillo. I brik vanno serviti caldi e potete accompagnarli con un'insalata fresca.

EMPANADAS di formaggio

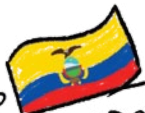

ECUADOR

RICETTA DI EVA

Ingredienti *(per 10/12 empanadas)*

Per l'impasto
½ kg di farina
1 uovo
50 gr di margarina
1 bicchiere di acqua tiepida (o latte)
1 cucchiaio di lievito secco per dolci
sale

Per il ripieno:
150 gr di formaggio (tipo mozzarella o formaggio dolce)
olio di semi di girasole
1 cipolla media

Preparazione

1. Per fare l'impasto (tapas) delle empanadas potete usare un asse di legno o il tavolo di cucina. Versate la farina, fate uno spazio al centro in cui aggiungere l'uovo, la margarina e un pizzico di sale. Con una forchetta mescolate gli ingredienti incorporando la farina poco per volta e versate a filo l'acqua tiepida. Lavorate l'impasto con le mani finché risulta morbido e fatelo riposare per 20 minuti, coperto con uno strofinaccio.

2. Nel frattempo preparate il ripieno. In una padella fate soffriggere la cipolla con un cucchiaino di olio d'oliva e lasciate raffreddare. A parte, tagliate il formaggio a pezzetti piccoli.

3. Passati 20 minuti, potete riprendere l'impasto, dividerlo in parti uguali e stendere ciascuna parte con il mattarello per farne dei dischi sottili, di 15 cm circa di diametro.

4. Su ciascun disco mettete un po' di cipolla e formaggio al centro. Piegate il disco a metà e chiudete perfettamente i bordi. Per chiudere le empanadas ognuno ha il suo metodo: un modo semplice è ripiegare piccole parti del bordo con pollice e indice verso l'interno e premere bene i bordi in modo che il ripieno durante la cottura non esca. In una teglia disponete le empanadas e infornate a 180° per 10-15 minuti.

Le empanadas sono tipiche dell'America Latina. Per il ripieno dipende dai gusti: formaggio e cipolla, oppure solo formaggio; cipolla, carota, piselli fatti cuocere in padella con un filo d'olio; pollo al vapore e olive; carne macinata con o senza pomodoro. Oltre al ripieno e alle spezie può variare l'impasto e la cottura: le empanadas possono essere fritte o al forno. Fanno parte della cucina dell'Ecuador, Uruguay, Argentina, Perù, Colombia e si trovano anche in Spagna.

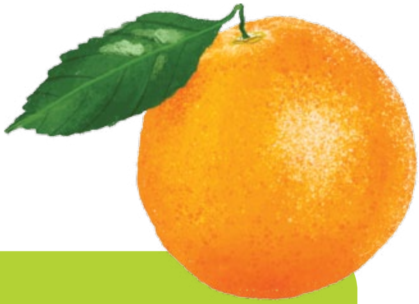

Alcune regole per un'alimentazione sana

Per avere un'alimentazione sana non è necessario stravolgere il proprio regime alimentare, basta seguire alcune semplici regole: non mangiare troppo (anche digiunare ogni tanto può fare bene oppure fare una cena leggera, con sole verdure cotte e crude); mangiare soprattutto alimenti vegetali come frutta e verdura (almeno 5 porzioni al giorno); pasta, riso, pane (meglio se integrali) e altri cereali (orzo, avena, farro, cous cous, miglio), tuberi e legumi. Mangiare carne non più di 2 volte alla settimana, evitando o riducendo al minimo le carni rosse o conservate (salumi e insaccati).
Il pesce è un alimento salutare e si consiglia di consumarlo almeno 2 volte alla settimana.
Formaggi e uova possono essere consumati al posto della carne, non in aggiunta a essa.
Il consumo di dolci (meglio se fatti in casa) dovrebbe essere contenuto e riservato alle occasioni speciali.
È preferibile condire e cucinare esclusivamente con olio extravergine di oliva e con poco sale.
Vanno evitati completamente i prodotti industriali con molte calorie e pochi nutrienti (cibo spazzatura) e le bevande zuccherate.

PEPERONI RIPIENI

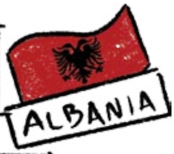

RICETTA DI ELSA

Ingredienti *(per 5 persone)*

5 peperoni
2 melanzane
1 bicchiere di riso
1 cipolla
origano
basilico
olio d'oliva
pepe
sale

Preparazione

1. Lavate tutte le verdure. Tagliate le melanzane a metà, eliminate la buccia e tritate la polpa. Tritate la cipolla sottile e preparate i peperoni togliendo la calotta, i semi e le parti bianche all'interno.

2. In una padella fate soffriggere la cipolla con un cucchiaio d'olio, aggiungete la polpa di melanzana, mescolate e fate cuocere a fuoco vivace qualche minuto. Abbassate la fiamma e continuate la cottura. Quando le melanzane hanno un bel colore aggiungete il riso e mezzo bicchiere d'acqua. Mescolate e fate cuocere con il coperchio a fiamma bassa. Quando il riso è cotto aggiungete origano, basilico, sale e pepe. Mescolate, assaggiate e lasciate raffreddare.

3. Uno alla volta, riempite i peperoni con l'impasto lasciando libero un centimetro dal bordo in modo che non fuoriesca durante la cottura. Chiudete i peperoni con la loro calotta e disponeteli in una teglia con un cucchiaio d'olio sul fondo. Infornate a 180° per 30 minuti.

I peperoni ripieni si possono mangiare con un'insalata fresca o una fettina di formaggio. In Albania ci sono diversi tipi di formaggi, freschi e stagionati. Per questo piatto è consigliato un formaggio di media stagionatura. In molti paesi ci sono delle varianti per il ripieno dei peperoni al forno. La ricetta di Marija e Dejan, ad esempio, suggerisce di riempire i peperoni con pollo, riso, patate, carote e cipolla. Pollo e riso vanno lessati in abbondante acqua, mentre le verdure vanno prima grattugiate e poi fatte stufare, aggiungendo alla fine un trito di erba cipollina. Con gli stessi ingredienti, in Serbia, si preparano gli involtini di verza (sarma), disponendo il ripieno sulle foglie appena lessate, poi chiuse come fagottini, oppure gli involtini con le foglie di cavolo cappuccio.

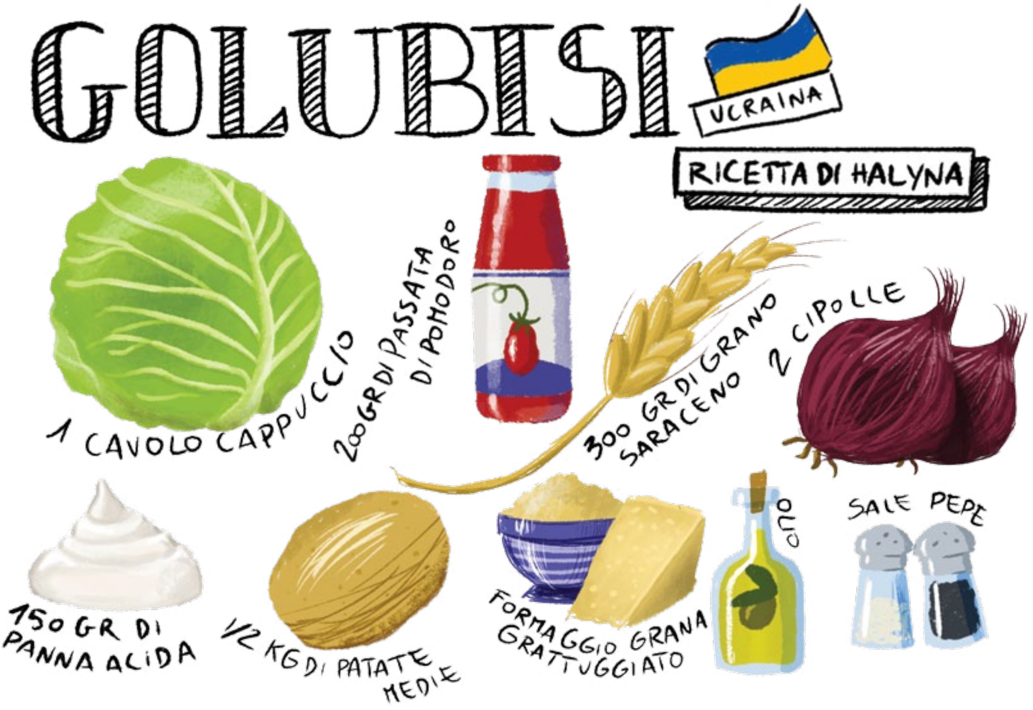

Ingredienti *(per 6 persone)*

1 cavolo cappuccio verde (circa 1 kg)
200 gr di passata di pomodoro
300 gr di grano saraceno
½ kg di patate medie
150 ml di panna acida
formaggio grana grattugiato
2 cipolle medie
olio d'oliva
pepe, sale

Preparazione

1. Lavate il cavolo e, aiutandovi con un coltellino, eliminate la parte centrale del gambo (in questo modo il cavolo si sfoglia durante la cottura). Fatelo sbollentare per qualche minuto in una pentola con abbondante acqua e un pizzico di sale. Scolate e scegliete le foglie migliori.

2. Pulite, pelate e grattugiate le patate. Pulite le cipolle, tagliatele sottili e fatele soffriggere. In una ciotola unite le patate grattugiate, la cipolla, il grano saraceno, formaggio grana grattugiato, pepe, sale, olio d'oliva. Mescolate e amalgamate bene gli ingredienti con un mestolo di legno.

3. Prendete le foglie di cavolo e, poche per volta, stendetele in modo che possano contenere qualche cucchiaio di ripieno, togliendo la parte dura

della foglia. Poi chiudete l'involtino: arrotolate la foglia di cavolo e chiudete bene alle estremità, rincalzando verso l'interno.

4. Disponete gli involtini in una pentola: mettete un filo d'olio, gli involtini uno accanto all'altro e poi a strati. Coprite appena con la passata di pomodoro, la panna acida e l'acqua.

5. Fate cuocere a fiamma bassa, con il coperchio, per circa 2 ore.

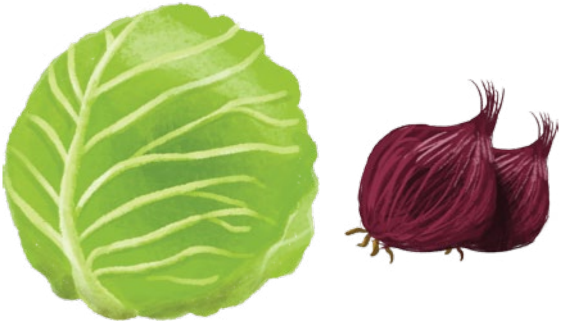

Golubtsi è un piatto tipico della cucina ucraina, presente anche in altri paesi dell'est Europa. Si possono usare le foglie di cavolo cappuccio o verza. La ricetta tradizionale è fatta con carne macinata e riso ma questa variante è altrettanto buona e più leggera. Se vi piace potete aggiungere anche dei funghi insieme alla passata di pomodoro.

TAMALES con verdure e pollo

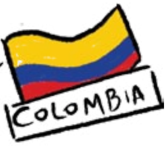

RICETTA DI MARIELA

Ingredienti *(per 5/7 persone)*

3 patate medie
100 gr di piselli
2 carote
1 cipolla o **1** porro
1 peperone
10 foglie di platano
300 gr di riso
(tipo thai o basmati)
500 gr di pollo
olio d'oliva
2 spicchi d'aglio
pepe, sale

Preparazione

1. Lavate il riso un paio di volte sotto l'acqua corrente e scolatelo. Lavate bene le foglie di platano, asciugatele con un panno e appoggiatele su un asse di legno o il tavolo in modo da avere spazio.

2. Tagliate il pollo a pezzetti e fatelo insaporire mezz'ora con due cucchiai di olio d'oliva, aglio e sale. Prendete una padella e fate cuocere il pollo per 10-15 minuti con il coperchio. Nel frattempo pulite tutte le verdure, lavatele e tagliatele sottili. I piselli possono essere freschi o surgelati.

3. In una padella fate soffriggere cipolla e peperone con un cucchiaio d'olio. Aggiustate di sale, pepe e fate cuocere qualche minuto.

4. Prendete una foglia di platano e, nel mezzo, mettete gli ingredienti a strati: un po' di riso, patate e carote (crudi), un po' di soffritto di cipolla e peperone, un cucchiaio di pollo, i piselli. Gli strati vanno fatti in base al tempo di cottura degli ingredienti. Chiudete bene la foglia di platano come un pacchettino e legate con lo spago per alimenti per non far uscire il ripieno durante la cottura.

5. In una pentola capiente fate bollire abbondante acqua. Quando avrete legato tutti i tamales, abbassate il fuoco al minimo, disponeteli sul fondo della pentola e chiudete con il coperchio. Fate cuocere per 40-50 minuti a fuoco medio. Quando le foglie di platano cambiano colore e diventano verde scuro i tamales sono pronti. Scolateli ancora caldi e fateli raffreddare qualche minuto prima di portarli a tavola.

In Colombia ci sono molte ricette per i tamales: potete farli con il mais al posto del riso, usando la farina per polenta precotta (bagnata con l'acqua fredda, così durante la cottura si gonfia); senza verdura, ripieni di riso e pesce (tempo di cottura 35-40 minuti), accompagnati con una salsa fatta con cipolla, peperone, aglio, latte di cocco. Potete trovare le foglie di platano nei negozi etnici. Vanno lavate bene anche se non si mangiano poiché durante la cottura sono a contatto con gli ingredienti. Se avanzano, potete conservare i tamales in frigo qualche giorno e scaldarli immergendoli in acqua bollente qualche minuto.

Cambiare abitudini, a piccoli passi con Meddie e Cleo in una App

Siamo sempre connessi, tutti abbiamo un cellulare. A volte questo crea dei problemi ma spesso la tecnologia ci aiuta anche a risolvere e velocizzare molte cose.
Per "Smuovi la salute" la Fondazione Bruno Kessler (FBK) ha realizzato una App da usare insieme, genitori e bambini, per imparare in famiglia alcune buone abitudini: mangiare più frutta e verdura, meno carne, muoversi dal divano, uscire a camminare.
Quanti passi hai fatto oggi? Hai giocato all'aria aperta? Scaricando l'App, i genitori aiutano i bambini a compilare un diario alimentare, per cui basta selezionare quello che abbiamo mangiato durante la giornata: tra alimenti e ricette di tante culture culinarie si fa in fretta. In più, si potrà tenere traccia di quanta attività fisica e movimento facciamo in settimana.
In questo modo, con dei messaggi divertenti, Meddie ci aiuterà a scoprire quante calorie ci sono in quello che mangiamo, se il cibo è nutriente, le porzioni troppo abbondanti o scarse.
Dialogando con Meddie, poco per volta, saremo più motivati a fare attenzione al nostro stile di vita e modificare alcune abitudini a piccoli passi.
Poi c'è Cleo e sarà davvero un'avventura trovare la Pietra Piramidale! Ogni indovinello risolto fa guadagnare dei punti che aiutano Cleo a proseguire il suo viaggio. Ma attenzione! Quando accettiamo una nuova sfida e raggiungiamo un obiettivo i punti raddoppiano.
Pronti al cambiamento? Mangiare la frutta 3 volte al giorno diventerà una bella abitudine, oltre che salutare.
Per scaricare l'App vai su Google Play (Android) o sull'App Store (iOS):

TORTILLAS di patate

RICETTA DI EVA

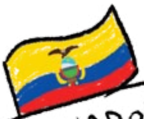

ECUADOR

Ingredienti *(per 5 tortillas)*

5/6 patate medie
1 porro
150 gr di formaggio
(mozzarella o formaggio dolce)
olio di semi di girasole
aglio in polvere
pepe, sale

Preparazione

1. Pelate le patate, tagliatele a metà e mettetele in una pentola. Coprite d'acqua, aggiungete poco sale e fatele bollire. Quando sono pronte, schiacciate le patate ancora calde come per fare il purè.

2. Nel frattempo, in una padella fate soffriggere il porro tagliato sottile con un cucchiaio d'olio d'oliva, aglio in polvere, pepe e sale. Fate cuocere piano mescolando con un mestolo di legno finché il porro diventa morbido.

3. A parte preparate il formaggio tagliato a pezzetti piccoli.

4. In una ciotola amalgamate le patate e il porro per farne un impasto morbido. Mescolate e aggiustate di sale e pepe. Prendete l'impasto con le mani, poco per volta, e fate delle palline. In ciascuna pallina inserite qualche pezzetto di formaggio, richiudete e schiacciate l'impasto per farne dei dischi di 2-3 cm di spessore.

5. Cuocete le tortillas in una padella antiaderente, con due cucchiai d'olio, girandole su entrambi i lati.

Le tortillas sono tipiche della cucina spagnola e latinoamericana e ci sono molti modi per farle: con o senza uova, carote, spinaci. Fritte o al forno. In questa ricetta non ci sono le uova e le tortillas non sono fritte nell'olio ma dorate, fatte cuocere con poco olio in una padella ben calda. È un piatto saporito che si può accompagnare con insalata fresca o verdure al vapore. Per fare le tortillas, in Ecuador si usa anche l'olio di achiote (ricavato dai semi di annatto) che conferisce profumo e colore agli alimenti.

VARENIKI con patate

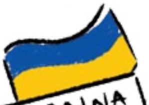

UCRAINA

RICETTA DI HALYNA

Ingredienti *(per 6 persone)*

500 gr di farina
1 uovo
5/6 patate (circa 500 gr)
200 ml di ricotta acida
(o ricotta dolce e succo di limone)
1 cipolla media
pepe, sale

Preparazione

1. Per l'impasto dei ravioli, mettete la farina sul piano del tavolo o un asse di legno. Al centro della farina ricavatevi uno spazio in cui mettere l'uovo, un pizzico di sale e, un po' alla volta, l'acqua. Aiutandovi con una forchetta cominciate ad amalgamare gli ingredienti, incorporando la farina e impastate. All'occorrenza, aggiungete poca acqua alla volta. Dovrete ottenere un impasto morbido.

2. Per il ripieno dei ravioli, lavate, sbucciate e fate bollire le patate in abbondante acqua, con un po' di sale, e scolate. Schiacciate le patate e lasciatele raffreddare in una ciotola a cui andrete ad aggiungere la ricotta acida, pepe e sale. Mescolate bene amalgamando gli ingredienti.

3. Stendete la pasta dei ravioli con il mattarello fino a renderla sottile (½ cm di spessore). Aiutandovi con un contenitore cilindrico, tagliate dei dischi di circa 5 cm di diametro (potete usare una tazza o un contenitore vuoto). Al centro dei dischi mettete un po' di ripieno e chiudete a metà, facendo combaciare i bordi. Schiacciate bene i bordi con le dita, da sinistra a destra, per non far uscire il ripieno durante la cottura.

4. Prendete una pentola capiente e fate bollire dell'acqua, a cui avrete aggiunto un po' di sale grosso: abbassate la fiamma e immergete i ravioli. Fate attenzione che non si attacchino sul fondo, mescolando piano con un mestolo. Da quando i ravioli vengono a galla, fate cuocere ancora 1 minuto e scolate. Potete condire i ravioli con olio d'oliva o un soffritto di cipolla e servire.

Vareniki è una ricetta tradizionale ucraina che si fa ogni domenica. Il ripieno può variare: grano saraceno e crauti; ricotta acida, pepe, sale. Per l'impasto si usa soltanto 1 uovo per 500 gr di farina. Con le dosi indicate in questa ricetta si possono fare 40 ravioli.

Con le verdure

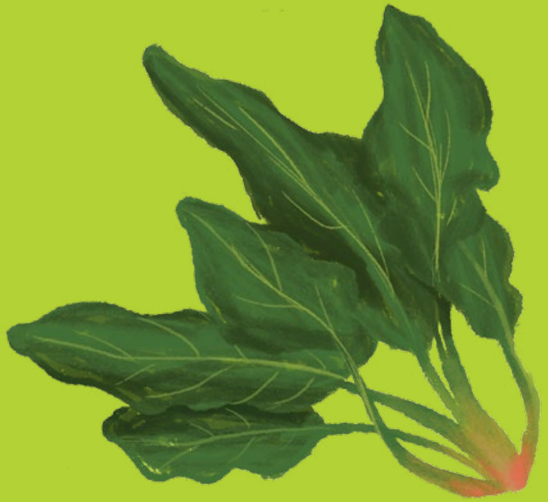

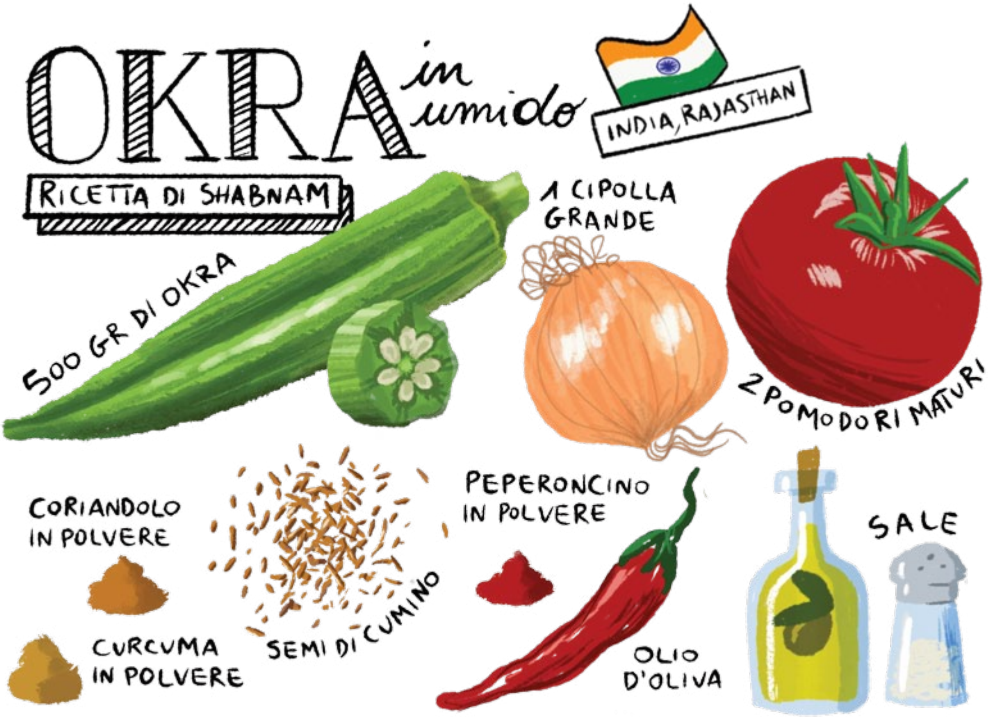

Ingredienti *(per 4 persone)*

500 gr okra
2 pomodori maturi
1 cipolla grande
semi di cumino
curcuma in polvere
coriandolo in polvere
peperoncino in polvere
olio d'oliva
sale

Preparazione

1. Lavate bene l'okra, spuntate le cime e tagliate a rondelle. Lavate i pomodori e tagliateli a pezzetti. Pelate la cipolla e tagliatela sottile.

2. In una padella, fate rosolare la cipolla con un cucchiaio d'olio d'oliva e semi di cumino. Aggiungete l'okra e, dopo qualche minuto, il pomodoro, un pizzico di sale e le spezie: ½ cucchiaino di curcuma, cumino, coriandolo, peperoncino a piacere. Mescolate bene e fate cuocere per 5-10 minuti.

L'okra (o ocra) è un ortaggio conosciuto anche come lady finger o gombo e viene usato nelle cucine di molti paesi. Fatto in umido, insieme ad altre verdure, è un piatto leggero e saporito. Si può mangiare con riso basmati o chapati: un pane sottile, tondo, simile alla piadina, fatto con acqua e farina integrale di frumento. La farina per chapati si trova in tutti i negozi etnici.

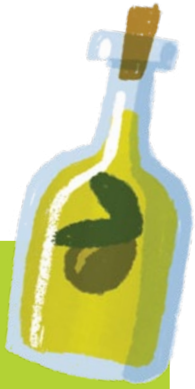

L'olio extravergine di oliva e le sue innumerevoli proprietà

Nella preparazione dei piatti è preferibile usare olio extravergine d'oliva per le sue proprietà.
L'olio d'oliva ha un elevato contenuto di acido oleico (un acido grasso mono-insaturo che lo compone al 75%), vitamine (A, B1, B2, C, D, E, K) e ferro. Contiene inoltre sostanze antiossidanti (come i polifenoli) che sono in grado di contrastare l'invecchiamento dei tessuti. È un'ottima fonte calorica (i grassi infatti costituiscono il 25-30% delle calorie giornaliere di una dieta bilanciata) ed è da preferire a grassi di origine animale (quali burro, lardo, pancetta) o industriale (margarina), ma anche ad alcuni olii vegetali (olio di arachidi, mais, girasole) che, a seguito di complessi processi di raffinazione, sono privi di quei composti antiossidanti e vitamine che caratterizzano l'olio di oliva.
Anche quando si tratta di rosolare o soffriggere gli alimenti l'olio extravergine d'oliva è preferibile. Per preservare i suoi elementi nutritivi (che sono danneggiati a partire dai 60°C) è consigliabile usarlo a crudo.

ALCUNI BAMBINI mangiano verdure

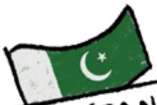

PAKISTAN

RICETTA DI ALISHA E ZAINAB

Ingredienti *(per 4 persone)*

1 busta di verdure miste surgelate
1 cipolla media
1 yogurt bianco
coriandolo in polvere
peperoncino in polvere
cumino in polvere
olio d'oliva
pepe, sale

Preparazione

1. In una padella fate soffriggere la cipolla tagliata sottile con un cucchiaio d'olio. Abbassate il fuoco e unite le verdure mescolando con un mestolo di legno. Aggiungete qualche tazzina d'acqua, sale e fate cuocere a fuoco medio finché le verdure sono cotte.

2. A parte, in una ciotola, preparate una salsa di yogurt bianco con le spezie: coriandolo, cumino, peperoncino, pepe e sale a piacere. Mescolate bene sbattendo con una forchetta.

3. Quando le verdure sono pronte mettetele in un piatto e portate in tavola insieme alla salsa di yogurt.

In questa ricetta abbiamo usato verdure surgelate: l'importante è controllare sempre l'etichetta e scegliere un prodotto di qualità. Le verdure migliori sono fresche ma quelle surgelate sono una valida alternativa specie se i ritmi di lavoro e gli impegni riducono il tempo a disposizione per la preparazione. Per questa ricetta potete scegliere un misto di carote, spinaci, piselli, broccoli da accompagnare alla salsa di yogurt e pane chapati o riso basmati.

ATTIÉKÉ al vapore

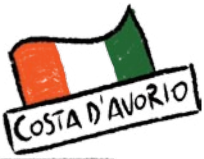

RICETTA DI AISHA

Ingredienti *(per 4/6 persone)*

4 porzioni di attiéké
2 cipolle medie
4 pomodori
peperoncino fresco
olio d'oliva
sale

Preparazione

1. Per cuocere l'attiéké potete usare una pentola a vapore (simile alla couscoussiera) oppure una pentola a cui sovrapporre un colino a rete o uno scolapasta in acciaio che dovrà rimanere sospeso in modo che la semola non sia a contatto con l'acqua e cuocia a vapore.

2. Versate l'acqua nella pentola e fatela bollire. Distribuite l'attiéké nella pentola con i buchi (o nel colino a rete), sgranate la semola con una forchetta e fate cuocere a vapore per 10-15 minuti.

3. A parte, in una padella antiaderente, fate rosolare la cipolla tagliata sottile con due cucchiai d'olio. Aggiungete peperoncino fresco (a piacere) e i pomodori a pezzetti. Mescolate, fate cuocere qualche minuto e aggiustate di sale.

L'attiéké è un alimento tipico della cucina della Costa d'Avorio. È fatto con la polpa fermentata di manioca che viene sbucciata, essiccata, cotta e grattugiata. Acquistato nei negozi etnici, si usa come il riso, il farro, il cous cous per accompagnare verdure stufate, pesce (per l'attiéké di garba si usa il tonno) e pollo (come per il kedjenou: pollo, melanzane, cipolle, pomodori e spezie). In questa ricetta l'attiéké è accompagnato con un sugo veloce di cipolle e pomodori. Il suo sapore, leggermente acidulo, lo rende adatto anche a insalate fresche.

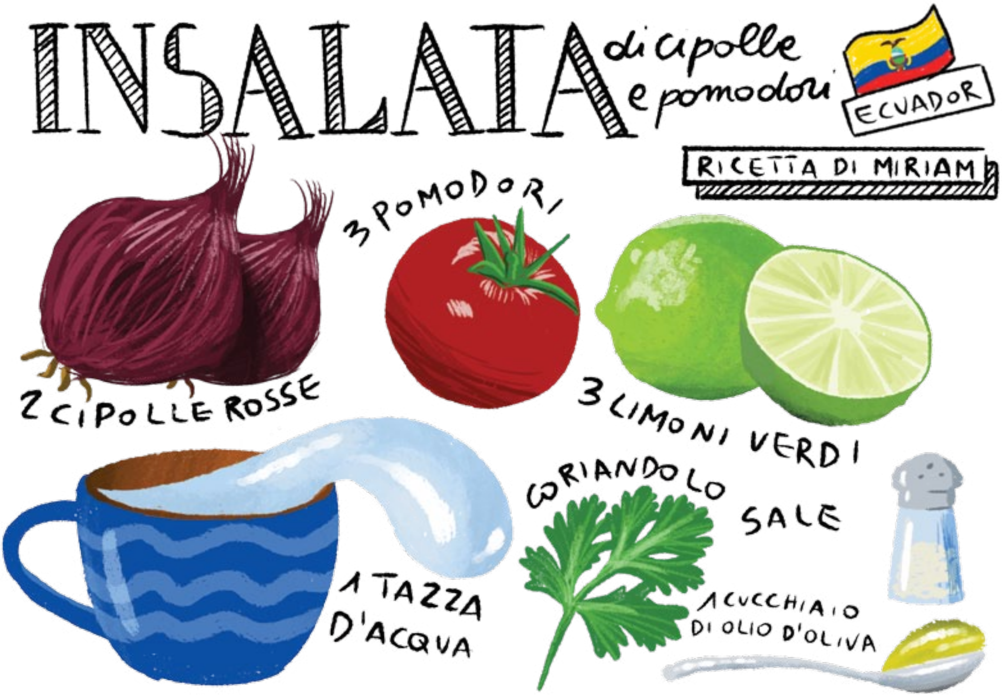

Ingredienti *(per 4 persone)*

2 cipolle rosse
3 limoni verdi
3 pomodori, tipo cuore di bue
1 cucchiaio di olio d'oliva
1 tazza di acqua
coriandolo fresco
sale

Preparazione

1. Pulite le cipolle e tagliatele a fettine sottili. Mettete le cipolle in una ciotola, aggiungete un pizzico di sale in modo da togliere l'acidità, il succo di ½ limone, 1 tazza di acqua tiepida. Fate marinare per 10 minuti, scolate e lavate le cipolle sotto l'acqua fredda. Mettete di nuovo le cipolle nella ciotola con tutto il succo di limone e fate riposare per 10-15 minuti.

2. Lavate i pomodori, tagliateli a fette e conditeli con le cipolle marinate, olio, sale e coriandolo fresco tritato.

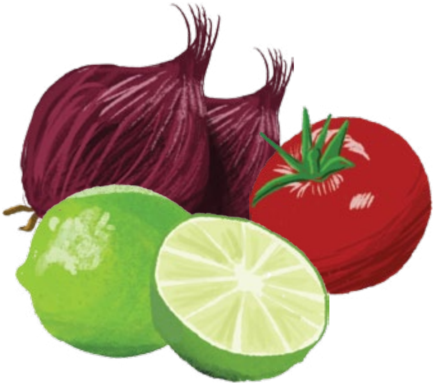

Le cipolle crude marinate nel limone sono il condimento base per molte insalate fresche nella tradizione culinaria dell'Ecuador. Oltre ai pomodori si possono aggiungere fagiolini, carote, patate o altre verdure di stagione.

INSALATA di rapanelli e uova sode

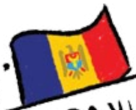

MOLDAVIA

RICETTA DI VERONICA

Ingredienti *(per 4 persone)*

2 mazzolini di rapanelli rossi
2 cipollotti bianchi freschi
finocchietto selvatico fresco
2 uova
olio d'oliva
sale

Preparazione

1. Pulite e lavate le verdure. Tagliate a rondelle sottili i rapanelli e i cipollotti freschi.

2. A parte, in un pentolino, preparate le uova sode: fate bollire l'acqua con una goccia di aceto, immergete le uova, fatele cuocere per 8-10 minuti e sbucciatele.

3. Sbriciolate le uova sode, unitele alle verdure e condite con un cucchiaio d'olio e sale.

È un'insalata fresca che può essere accompagnata con purè di patate. All'insalata si possono aggiungere anche cetriolini, pomodori e peperoni a rondelle oppure, d'estate, cavolo cappuccio tagliato sottile.

GRATIN di PATATE

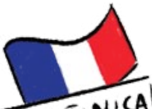

MARTINICA

RICETTA DI JOSIANE

Ingredienti *(per 6 persone)*

4 patate medie
6 banane platano verdi
(oppure **8** zucchine)
1 pugno di grana o emmenthal grattugiato
1 pugno di pangrattato
½ l di latte
25 gr di burro
noce moscata
pepe, sale

Preparazione

1. Lavate, sbucciate e tagliate a fette le patate e le banane platano verdi. Mettetele in una pentola e coprite con acqua fredda. Portate a bollore e fate cuocere aggiungendo poco sale. Scolate e lavorate gli ingredienti ancora caldi con il passaverdure.

2. A parte, scaldate il latte con poco burro, pepe e noce moscata. Unite patate e banane platano verdi al latte caldo, mescolate con una frusta e fate cuocere a fiamma bassa. Aggiungete grana grattugiato e mescolate. Se il purè risulta troppo denso, aggiungete ancora latte, continuando a mescolare.

3. Quando è pronto, versate il purè in una pirofila, con un cucchiaio d'olio sul fondo, e distribuitelo bene su tutta la superficie. Spolverate con pangrattato e infornate a 180° per 20 minuti.

Potete sostituire le banane platano verdi con delle zucchine o zucchine spinose (sechium edule), conosciute come christophine e chayote. È un ortaggio poco conosciuto in Italia che appartiene alla famiglia delle Cucurbitacee, come zucchine, zucche, cetrioli, cocomeri e meloni. Le banane platano verdi equivalgono al platano usato in molte ricette africane. Lo potete trovare nei negozi etnici e in alcuni supermercati.

INSALATA di JARDA

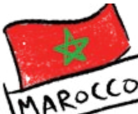

MAROCCO

RICETTA DI FOUZIA

Ingredienti *(per 4 persone)*

2 tazze di riso
4 tazze di acqua
4 carote
4 patate
3 rape rosse
2 manciate di fagiolini
2 manciate di piselli o fave
1 barattolo di mais
1 grappolo di pomodorini
foglie di insalata
olio d'oliva
sale

Preparazione

1. Fate cuocere il riso in un pentolino con 4 tazze d'acqua: per 5 minuti a fiamma alta, poi a fiamma bassa per 10 minuti con il coperchio. Quando il riso è pronto versatelo in una ciotola insieme al mais e mescolate.

2. Pulite e lavate tutte le verdure. Fate cuocere carote, patate, fagiolini e piselli in una pentola capiente, con poca acqua sul fondo, all'interno di un cestello per la cottura a vapore. Potete cuocere le verdure intere, scolarle quando sono ancora croccanti e tagliarle alla fine. Se le rape rosse sono fresche, fatele lessare in un pentolino a parte, per evitare che colorino le altre verdure (in alternativa potete acquistarle lessate).

3. Quando le verdure sono pronte, prendete un piatto tondo, di quelli grandi da portata. Mettete le foglie di insalata sul fondo e cominciate a comporre il piatto: al centro mettete il riso e tutte le verdure intorno, a spicchi, evitando di mescolarle. Lungo il bordo aggiungete i pomodorini.

4. Per condire l'insalata, preparate una ciotolina con tre cucchiai di olio d'oliva, sale, pepe e prezzemolo tritato da versare a filo sul piatto.

È un'insalata fresca che va bene tutto l'anno, scegliendo verdure di stagione in base ai propri gusti: rapanelli, cetrioli, cime di rapa o broccoli, alternando verdure cotte e crude. Per ottenere un buon risultato l'importante è che le verdure siano croccanti, non troppo molli, e mettiate fantasia nella composizione del piatto come se doveste decorare un dolce, alternando colori e geometrie. Si possono aggiungere anche tonno, per dare colore al riso, e uova sode grattugiate per separate tra loro gli spicchi di verdure.

VERDURE SALTATE

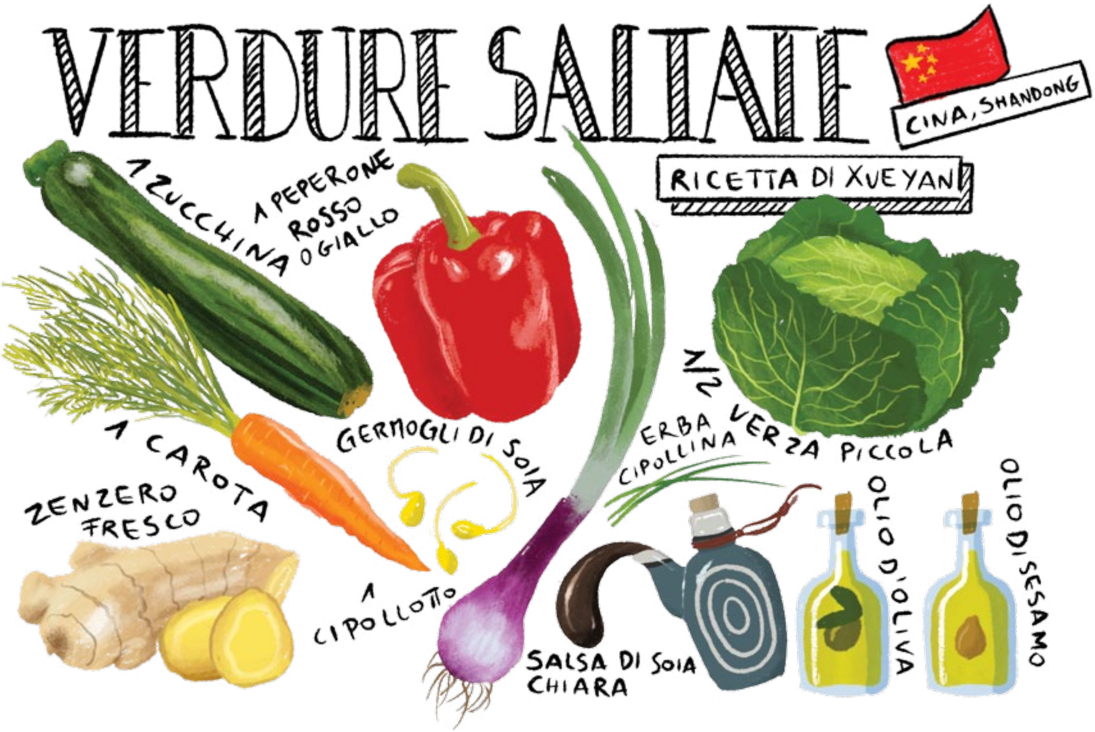

Ingredienti *(per 2 persone)*

1 zucchina
1 carota
1 peperone rosso o giallo
½ verza piccola
1 cipollotto
erba cipollina
germogli di soia
zenzero fresco
salsa di soia chiara
olio d'oliva
olio di sesamo

Preparazione

1. Pulite e lavate tutte le verdure e tagliatele a fettine sottili piuttosto lunghe.

2. Mettete il wok sul fuoco a fiamma alta. Quando è ben caldo, aggiungete un cucchiaio d'olio, qualche fettina di zenzero e le verdure in base ai tempi di cottura: zucchine, carote, peperoni. Fate rosolare velocemente, poi unite la verza, i germogli di soia e il cipollotto. Aggiungete un cucchiaio di salsa di soia, due cucchiai d'acqua e fate saltare le verdure a fiamma alta. A fine cottura potete aggiungere un cucchiaino di olio di sesamo per insaporire il piatto.

È un piatto facilissimo e veloce da preparare. Si possono usare una varietà di verdure fresche, in base alla stagione e ai prodotti che si trovano in negozio o al mercato. Per preparare un piatto equilibrato potete considerare circa 40 gr a persona per ciascun tipo di verdura: carote, zucchine, peperoni, verza e altri ortaggi che preferite.

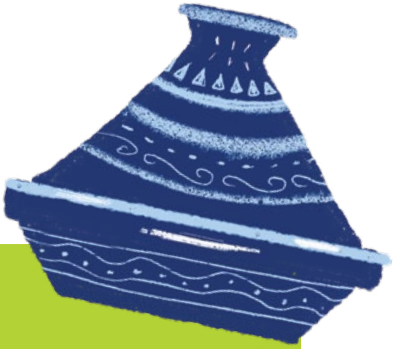

Modalità di cottura e preparazione dei piatti

Alcune ricette possono sembrare elaborate, con tanti ingredienti e diversi tipi di cottura, mentre il ritmo di vita è frenetico e acquistare un piatto pronto sembra la soluzione. Sono cotture al vapore, al forno, nella tajine. Zuppe che cuociono a fuoco lento ma con la pentola a pressione si possono ridurre i tempi di cottura.
La tajine è una pentola di terracotta usata in tutto il Magreb e nei Paesi del Sud del Mediterraneo. È fatta di due elementi: un piatto su cui appoggiare gli ingredienti e un coperchio a forma di cono che fa cuocere il cibo in modo sano, lento, conservando tutte le proprietà. Nella tajine, in base alle quantità, verdure e pesce cuociono da sole. Non si abbandona la pentola sul fuoco ma non serve girare di continuo. Lo stesso vale per la cottura al vapore, se fate attenzione a dosare la quantità d'acqua.
Spesso si tratta di trovare il tempo di lavare, pulire, sbucciare e tagliare le verdure, scegliendo quelle di stagione. Meglio non cuocerle troppo così mantengono tutte le proprietà.

TAJINE di verdure

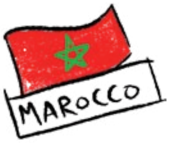

RICETTA DI AMAL

Ingredienti *(per 4 persone)*

½ cavolfiore
1 finocchio
1 cipolla bianca
3 patate medie
4 carote
100 gr piselli
100 gr fave
zenzero
prezzemolo
curcuma
1 spicchio d'aglio
pepe, sale

Preparazione

1. Pulite e lavate tutte le verdure. Tagliate le cipolle, le carote e le patate a rondelle; il finocchio in quarti e poi a fettine; il cavolfiore a mazzolini e i gambi a rondelle (togliendo le parti più fibrose). I piselli possono essere freschi o surgelati; le fave, se sono secche vanno messe in ammollo (per le fave decorticate ammollo di 8 ore; per le fave con la buccia ammollo di 16 ore), se sono fresche e tenere vanno sgranate, togliendo la pellicina bianca esterna.

2. Prendete la tajine e disponete gli ingredienti in cerchi concentrici: uno strato di cipolle su tutto il piatto; uno strato di patate, lasciando uno spazio per piselli e fave sul bordo esterno; uno strato di carote; uno strato di finocchio; il cavolfiore al centro.

3. Prendete una ciotola in cui mettere: un po' di sale, pepe, zenzero in polvere (o grattugiato fresco), aglio e prezzemolo tritati, curcuma in polvere, olio d'oliva e un po' d'acqua. Sbattete bene con la forchetta per emulsionare e versate la salsa sulle verdure.

4. Chiudete la tajine e fate cuocere a fiamma bassa per 1 ora circa. Di tanto in tanto sollevate il coperchio per controllare la cottura delle verdure.

Ci sono tajine di diverse misure, come scegliere quella giusta? In base al numero di persone per cui cucinate, alla quantità di verdure o altri ingredienti che andrete a mettere sul piatto. Usare una tajine grande per piccole quantità non va bene perché il calore si disperde. Il consiglio è di scegliere una tajine della misura adatta, come per le altre pentole che avete in casa.

VERDURE in UMIDO

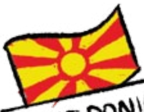

MACEDONIA

RICETTA DI IMERI

Ingredienti *(per 4 persone)*

3 peperoni gialli o rossi
5-6 pomodorini
1 cipolla bianca
paprika dolce
olio d'oliva
sale

Preparazione

1. Lavate i peperoni, togliete la calotta e i semi. Lavate e pulite i pomodori e la cipolla.

2. Tagliate la cipolla sottile, i peperoni a pezzetti piccoli, i pomodorini in quarti.

3. In una padella fate soffriggere la cipolla, aggiungete i peperoni, un pizzico di sale e fate cuocere per 20 minuti finché diventano morbidi, mescolando di tanto in tanto. Aggiungete i pomodorini e paprika a piacere. Fate cuocere ancora qualche minuto e servite.

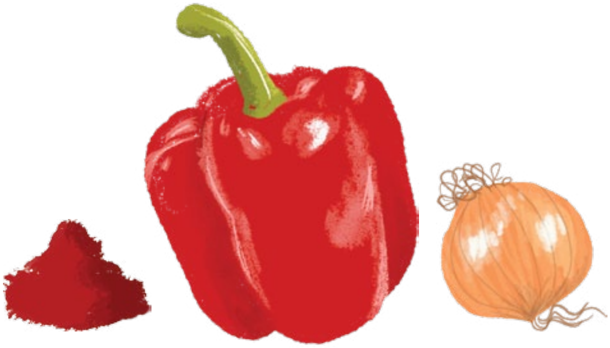

È un piatto semplice che si può mangiare caldo o freddo, insieme al pane o al riso. Con lo stesso procedimento si possono cuocere altre verdure: zucchine, melanzane, piselli, carote e cavolfiore in base alla stagione, insieme a un rametto di timo, origano o maggiorana.

Con
i legumi

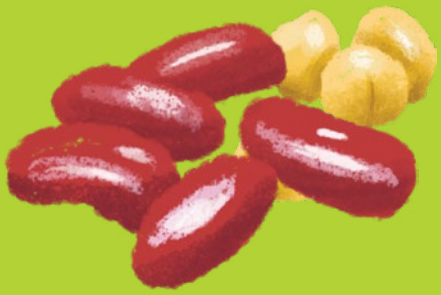

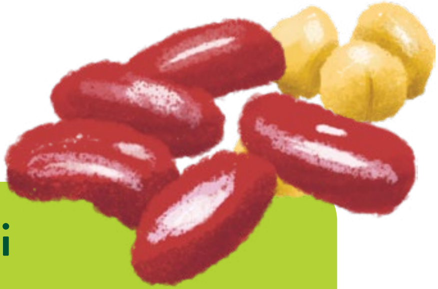

I legumi sono gustosi alleati della nostra salute

Fagioli, piselli, lenticchie, ceci sono tra i legumi più conosciuti in cucina. Ne esistono in realtà moltissime specie - tra cui soia, arachidi, lupini, cicerchie - e altrettante varietà.
Se pensiamo ai fagioli, tra borlotti, cannellini, fagioli dall'occhio e alcune varietà locali la scelta è davvero ampia. Il loro impiego in cucina va dalla preparazione di zuppe, minestre, primi piatti, salse e insalate.
I legumi sono alimenti poveri di grassi, contengono fibre, quantità apprezzabili di vitamine del gruppo B (B1, B2, niacina, acido folico) e vitamina C (se sono freschi), minerali (potassio, calcio, ferro) e sono un'importante fonte di proteine tanto da meritarsi il titolo di "carne dei poveri".
I legumi però mancano di alcuni elementi essenziali che compongono le proteine (in particolare gli amminoacidi solforati, come metionina e cisteina) presenti tuttavia in abbondanza nei cereali. Per avere quindi un apporto bilanciato di proteine, anche in alternativa alla carne, si consiglia di assumere i legumi in combinazione con i cereali (meglio se integrali). Ecco perché molti abbinamenti delle cucine tradizionali, come riso e fagioli, riso e ceci, cous cous con lenticchie, hummus e pane integrale, sono combinazioni perfette dal punto di vista nutrizionale, oltre che piatti gustosi.
Nelle preparazioni, se sono secchi, occorre metterli in ammollo prima e i tempi di cottura variano in base al tipo. Se il tempo a disposizione è poco si possono usare legumi già cotti, prediligendo le confezioni di vetro rispetto a quelle di latta.
È preferibile scegliere prodotti senza conservanti, sciacquando a lungo i legumi prima di utilizzarli, in modo da ridurne il sale. Per renderli più digeribili si possono acquistare legumi decorticati o usare il passaverdure per eliminare le bucce. In questo modo saranno apprezzati anche dai più piccoli.

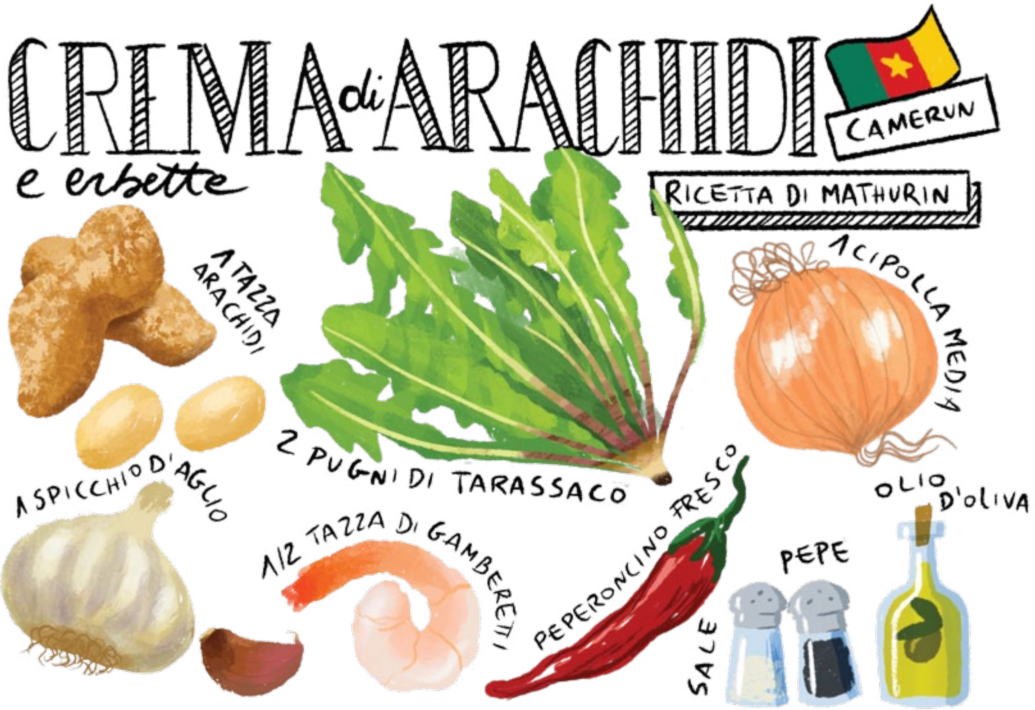

Ingredienti *(per 4 persone)*

1 tazza di arachidi sgusciate (non tostate)
2 pugni di tarassaco (o altre erbette amare)
½ tazza di gamberetti
1 cipolla media
1 spicchio d'aglio
peperoncino fresco
olio d'oliva
pepe, sale

Preparazione

1. Mettete in ammollo le arachidi per ammorbidirle e togliere la pellicina. Quando le arachidi sono pulite, fatele cuocere in un pentolino, coperte d'acqua, per 10 minuti a fuoco basso. Scolate le arachidi e frullatele con il mixer.

2. Lavate le foglie di tarassaco più volte finché l'acqua rimane pulita. In una pentola con dell'acqua sbollentate le foglie di tarassaco. Poi scolatele e fatele raffreddare qualche minuto. Strizzate il tarassaco e, appoggiandovi su un tagliere, tritatelo con un coltello.

3. Lavate i gamberetti sotto l'acqua correte, pulite la cipolla e tagliatela a rondelle sottili.

4. In una pentola fate rosolare cipolla, aglio e peperoncino con un cucchiaio d'olio. Aggiungete il tarassaco, le arachidi frullate e mezzo bicchiere d'acqua. Mescolate e fate cuocere per 15 minuti a fuoco basso. A metà cottura unite i gamberetti, aggiustate di sale e pepe.

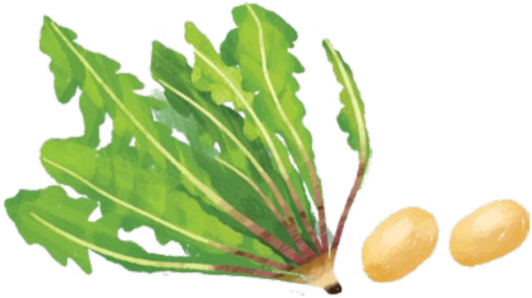

La ricetta originale di questo piatto è fatta con foglie amare (ndole) che crescono spontanee in Africa e sono difficili da trovare. Potete sostituirle con il tarassaco o altre erbette amare - cicorie, radicchio, spinaci - che trovate fresche in base alla stagione. Altri ingredienti previsti nella ricetta tradizionale sono il njangsa (semi ricavati dai frutti di un albero che cresce nella zona tropicale) e troverete varianti di questo piatto con la carne e il pesce.

CREMA di FAGIOLI

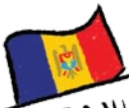

MOLDAVIA

RICETTA DI VERONICA

Ingredienti *(per 4 persone)*

400 gr di fagioli cannellini
1 cipolla bianca
1 spicchio d'aglio
pepe nero macinato
olio d'oliva
sale

Preparazione

1. Mettete in ammollo i fagioli secchi la sera prima di cucinarli. Prima di cuocerli dovrete scolarli e sciacquarli sotto l'acqua corrente.

2. Fate cuocere i fagioli in una pentola capiente, coperti d'acqua, a fuoco medio per 40 minuti circa. Quando sono pronti raccoglieteli con una schiumarola e passateli con il mixer per farne una crema, aggiungendo poca acqua di cottura.

3. A parte fate soffriggere la cipolla, tagliata sottile, con un cucchiaio d'olio e uno spicchio d'aglio.

4. Servite la crema di fagioli con un cucchiaio di cipolla, pepe nero e olio d'oliva.

È una crema di fagioli semplice. Per farla si possono usare sia i cannellini che i fagioli dall'occhio. In Moldavia in genere si usa olio di semi di girasole: nelle zone rurali le famiglie portavano i semi al mulino per ricavarne l'olio. Questa crema leggera si usava in occasione dei funerali e nei periodi di digiuno.

FAGIOLI in UMIDO

NIGERIA

RICETTA DI PATIENCE

Ingredienti *(per 4 persone)*

500 gr di fagioli dall'occhio secchi
1 cipolla bianca
1 pomodoro
olio di semi di girasole
sale

Preparazione

1. Mettete in ammollo i fagioli la sera prima. Scolateli e sciacquateli prima di cucinarli.

2. In una pentola capiente mettete un cucchiaio d'olio, cipolla tritata, pomodoro a pezzetti, poco sale e fate cuocere qualche minuto. Aggiungete i fagioli e coprite d'acqua.

3. Fate cuocere a fiamma bassa per 40 minuti, finché l'acqua si asciuga. Assaggiate, mescolate e aggiustate di sale.

Anziché fagioli secchi si possono usare fagioli pronti, già lessati. Fate però attenzione all'etichetta perché contengono sale e conservanti. Per la cottura tenete da parte un pentolino d'acqua calda: se i fagioli diventano troppo asciutti potete aggiungerne poca per volta. Potete accompagnare i fagioli in umido con riso bianco o platano.

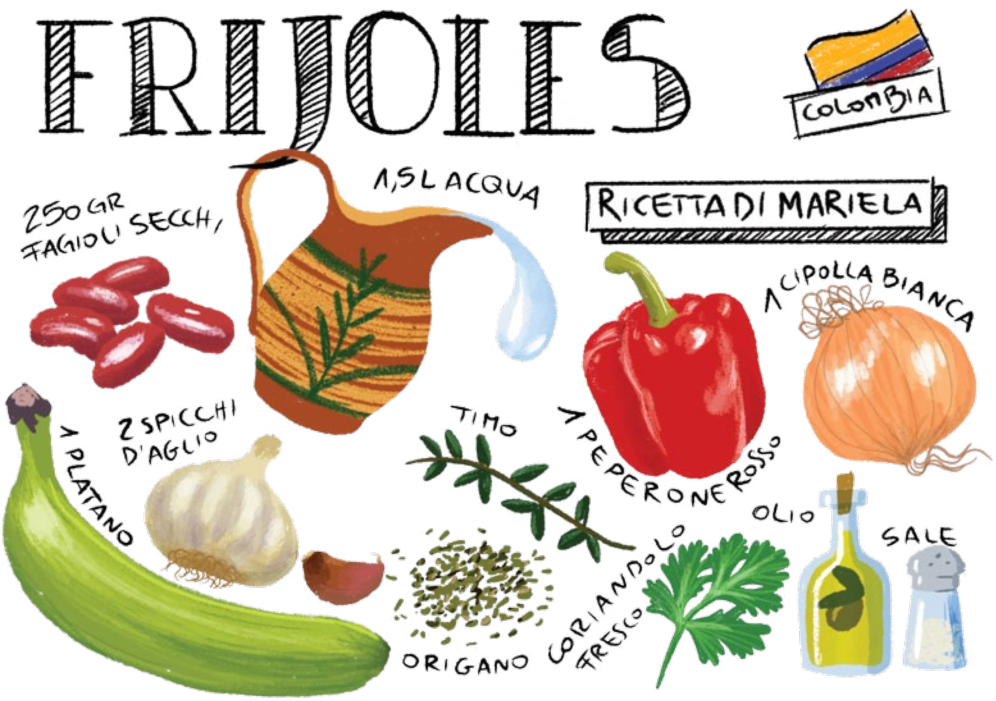

Ingredienti *(per 5 persone)*

250 gr di fagioli borlotti secchi
1,5 l di acqua
1 peperone rosso
1 cipolla bianca
1 platano o **2** patate medie
2 spicchi d'aglio
coriandolo fresco
origano
timo
olio d'oliva
sale

Preparazione

1. Pulite e lavate tutte le verdure. Tagliate il platano (o le patate) e i peperoni a pezzetti piccoli, la cipolla sottile, il coriandolo tritato.

2. Mettete in ammollo i fagioli una notte intera. Prima di cuocerli, scolate i fagioli e sciacquateli sotto l'acqua fredda. Metteteli in una pentola, copriteli d'acqua e fateli cuocere lentamente. Alla fine aggiungete il platano (o le patate), mescolate e proseguite la cottura.

3. Nel frattempo preparate un soffritto con un cucchiaio d'olio d'oliva, cipolla, peperone, aglio. Fate cuocere qualche minuto, aggiungete origano, timo e aggiustate di sale.

4. Unite il soffritto ai fagioli e fate cuocere ancora 10 minuti. Quando i frijoles sono pronti, toglieteli dal fuoco e aggiungete coriandolo fresco.

I frijoles si mangiano durante la settimana, non è un piatto delle feste, e vanno serviti caldi. Possono essere accompagnati da riso thai o basmati. In alternativa ai borlotti potete usare fagioli dell'Ecuador oppure fagioli neri. In America Latina ci sono moltissime varietà di legumi con cui si fanno zuppe o piatti semplici e speziati. Trovate il platano nei negozi etnici oppure potete usare le patate: entrambi ricchi di amido.

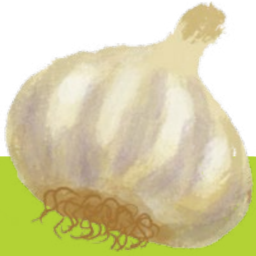

Sprigionare le sostanze salutari contenute nell'aglio

L'aglio contiene una sostanza denominata Allicina, quella che dà all'aglio il suo odore caratteristico. Questa sostanza, che deriva da un precursore inodore (Alliina), ha molte proprietà salutari: è anti-ossidante e quindi protegge le cellule dall'aggressione dei radicali liberi che sono cancerogeni. Inoltre riesce ad arrestare la divisione cellulare e contrasta anche l'angiogenesi, cioè la creazione di nuovi vasi sanguigni che favoriscono la sopravvivenza e la crescita dei tumori.
Si ritiene che l'Allicina riesca anche ad ostacolare la migrazione di cellule tumorali, con possibili effetti protettivi rispetto alla metastatizzazione dei tumori. Questi effetti sono stati testati e accertati non solo negli studi eseguiti in laboratorio ma anche osservando cosa succede a chi consuma aglio nella propria alimentazione.
Le evidenze di un effetto protettivo dell'aglio riguardano diverse forme di cancro: allo stomaco, alla prostata, al colon e, con evidenze meno forti, al polmone, alla mammella, alla laringe, all'esofago e al cavo orale.
Per trasformare l'Alliina in Allicina le cellule di cui è composto l'aglio devono rompersi prima della cottura. Il metodo più efficace è quello di schiacciare l'aglio con una forchetta, premendo forte, e rosolarlo o cuocerlo. La cottura dello spicchio intatto comporta solo la denaturazione dell'Alliina senza che si formi la preziosa Allicina (e tagliarlo dopo la cottura non cambia niente).
Anche sminuzzare l'aglio prima di cuocerlo va bene, ma l'aglio schiacciato funziona ancora meglio. [Fonte: Siegfried Knasmüller (Hrsg), Krebs und Ernährung, Georg Thieme Verlag, Stuttgart, 2014, pagina 330-335]

GNOCCHETTI di fagioli dall'occhio

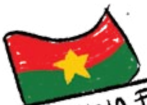

BURKINA FASO

RICETTA DI AMI

Ingredienti *(per 4 persone)*

200 gr di fagioli dall'occhio
1 pugno di foglie
di fagioli dall'occhio

2 cucchiaini di bicarbonato
olio d'oliva
sale

Preparazione

1. Lasciate in ammollo i fagioli tutta la notte. Scolateli e tritateli finissimi, aggiungendo il bicarbonato sciolto in poca acqua. I fagioli devono diventare una crema morbida e liscia per cui occorre usare un tritatutto o un robot da cucina.

2. A parte, in una ciotola, fate ammorbidire le foglie dei fagioli in acqua fredda. Aggiungete le foglie dei fagioli alla crema e tritate bene in modo da amalgamare gli ingredienti. Se preferite potete aggiungere le foglie soltanto a una parte della crema di fagioli in modo da avere gnocchetti di due colori e sapori diversi.

3. Per cuocere l'impasto usate una pentola per la cottura a vapore: una couscoussiera o vaporiera, con una pentola sotto in cui mettere l'acqua e un'altra, sovrapposta, con i buchi. Poiché la crema è morbida preparate dei coni con la carta forno da usare per la cottura: ritagliate 4-5 fogli di carta e arrotolate ciascun foglio per fare dei coni che dovrete chiudere bene in fondo. Distribuite la crema di fagioli all'interno dei coni di carta e adagiateli nella pentola con i buchi. Fate cuocere la crema di fagioli a vapore per 40 minuti.

4. Quando l'impasto è pronto, appoggiate i coni su un piatto, apriteli e aiutandovi con una forchetta dividete l'impasto per fare degli gnocchi. Non preoccupatevi se la forma è irregolare. Distribuiteli nei piatti con un pizzico di sale e un cucchiaio d'olio a freddo.

Per fare questa ricetta oltre ai fagioli servono anche le foglie che possono essere raccolte fresche e congelate. In alternativa potete fare gli gnocchi soltanto con l'impasto di fagioli, senza le foglie. Nella ricetta tradizionale, al

posto del bicarbonato si usa il potassio ricavato dalla cenere degli steli di miglio con una lavorazione particolare. Il bicarbonato ha la funzione di ammorbidire i legumi e renderli più digeribili.

Con il pollo

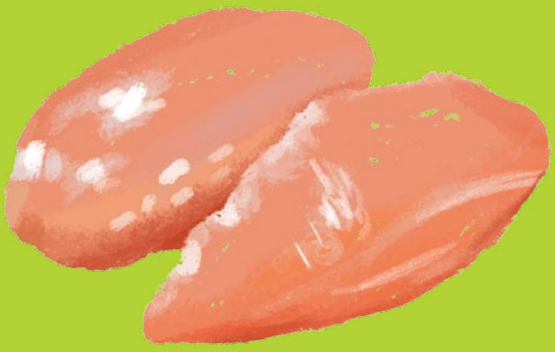

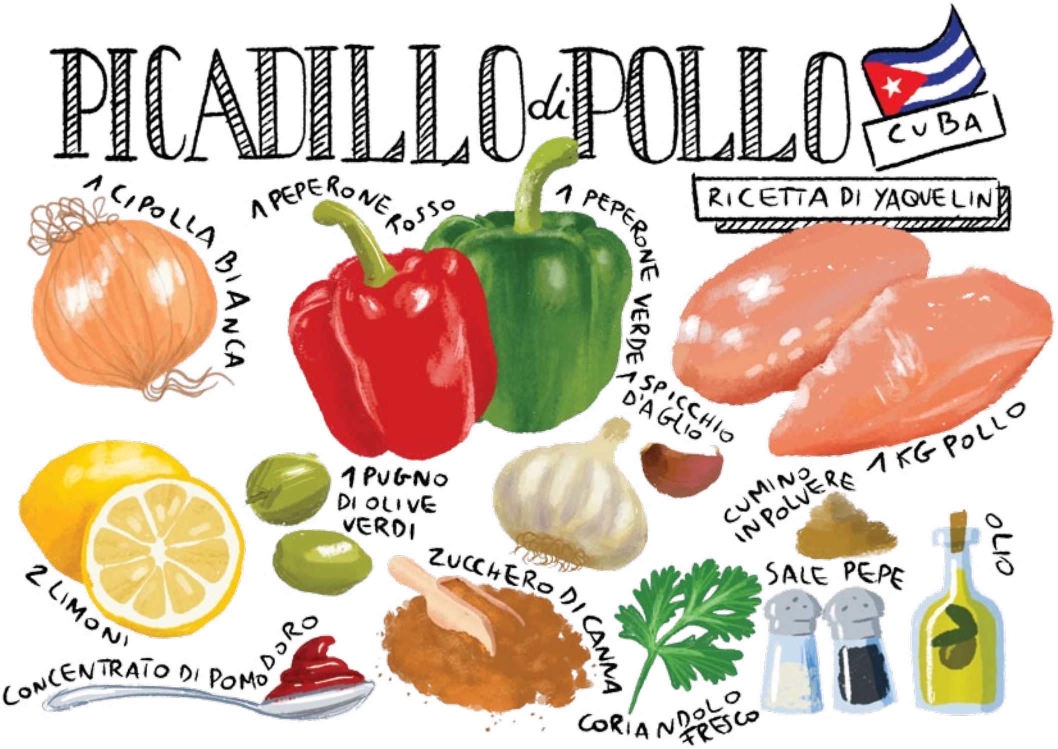

Ingredienti *(per 6 persone)*

1 kg di pollo
1 peperone rosso
1 peperone verde
1 cipolla bianca
concentrato di pomodoro
1 pugno di olive verdi
2 limoni
cumino in polvere
coriandolo fresco
zucchero di canna
olio d'oliva
1 spicchio d'aglio
pepe, sale

Preparazione

1. Tagliate a pezzetti piccoli il pollo e fatelo marinare con il succo di limone per 10 minuti.

2. Lavate tutte le verdure, tagliate la cipolla sottile e i peperoni a quadratini piccoli.

3. In una padella fate rosolare la cipolla con due cucchiai di olio d'oliva e uno spicchio d'aglio. Aggiungete il pollo, i peperoni, la punta di un cucchiaino di cumino in polvere, coriandolo fresco. Regolate di sale e

fate cuocere a fuoco basso per 30 minuti. Verso fine cottura aggiungete un cucchiaino di concentrato di pomodoro, le olive, 1 cucchiaino di zucchero di canna.

Il picadillo cubano in genere viene fatto con carne macinata e viene accompagnato con riso basmati o platano fritto. Se vogliamo portare a tavola un piatto sano e mantenere i sapori della cucina cubana, possiamo fare il picadillo con il pollo, da mangiare con riso basmati o manioca cotta al vapore.

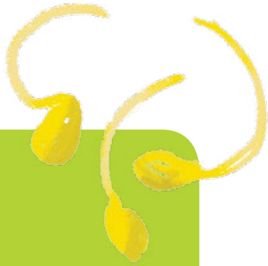

Come usare la soia nella preparazione di alcuni piatti

Come tutti i legumi, anche la soia è ricca di proteine, fibre, minerali e può sostituire la carne in diverse preparazioni.
La soia disidratata si trova in diversi formati: in base alla ricetta potete scegliere granulato o bocconcini di soia. Non ha un sapore deciso, è un alimento piuttosto neutro per cui è consigliabile usare erbe aromatiche (rosmarino, alloro, finocchietto selvatico, timo, salvia, origano) e spezie (noce moscata, paprika dolce e piccante, curry, bacche di ginepro o radice di curcuma in base alla ricetta).
Prima di cucinarla, la soia va reidratata: è sufficiente coprirla con un buon brodo vegetale freddo, che sia ricco di sapore e appena salato, farla bollire per 5 minuti e raffreddare nel brodo.
La soia è un buon sostituto della carne e viene cucinata in modo simile, tenendo conto di alcune cose: una volta reidratata è già cotta, non serve rosolarla e, in base alla ricetta, potete seguire i tempi di cottura degli altri ingredienti.
In generale, la soia può sostituire la carne in tutte le preparazioni ma vanno evitate le cotture troppo asciutte, oppure potete conservare qualche mestolo di brodo vegetale e aggiungerne poco per volta, per ultimare la cottura. Seguendo questa preparazione, i bocconcini di soia reidratati possono sostituire piatti con il pollo o il maiale.

COLOMBO de POULET

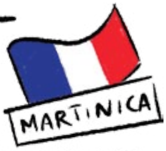

RICETTA DI JOSIANE

Ingredienti *(per 6 persone)*

600 gr di cosce di pollo
1 rapanello lungo bianco
2 manghi verdi (oppure carote)
1 melanzana
1 cipolla
1 lime
peperoncino fresco o in polvere
1 ciuffo di prezzemolo
3 spicchi d'aglio
1 cucchiaio di aceto bianco
3 chiodi di garofano
erba cipollina
cannella
zenzero
2 cucchiaini di curry
1 pezzetto di tamarindo fresco (facoltativo)
olio di semi di arachidi
pepe, sale

Preparazione

1. Lavate, pulite e tagliate tutte le verdure: la cipolla sottile, le altre verdure a fettine non troppo grosse. Tritate il prezzemolo, l'erba cipollina e un pezzetto di tamarindo tenendoli separati.

2. Tagliate il pollo a pezzi grossi e fatelo rosolare in una padella con due cucchiai d'olio. Aggiungete sale, pepe, peperoncino e fatelo cuocere a fiamma bassa.

3. A parte, in una padella antiaderente fate rosolare tutte le verdure con un cucchiaio d'olio. Mescolate e aggiungete poca acqua se necessario.

4. Unite poi il pollo e le verdure nella stessa pentola, aggiungete l'aceto e le spezie, coprite appena d'acqua e continuate la cottura a fuoco medio per 45 minuti con il coperchio.

5. A fine cottura aggiungete il succo di lime. Mescolate, cuocete ancora 10 minuti e servite.

Potete accompagnare questo piatto con purè di patate o riso basmati. Questa ricetta prende il nome dalla varietà di curry (colombo) che si usa nella cucina delle Antille. Nei negozi etnici si trova una miscela di 4 spezie (pepe, cannella, zenzero, timo) perfette per questa ricetta. Il tamarindo è facoltativo (non sempre si trova fresco). Al posto del mango verde potete usare le carote.

OCRA con POLLO

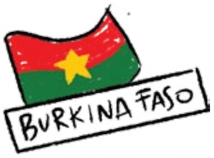

BURKINA FASO

RICETTA DI ALIGUETON E RASSIDE

Ingredienti *(per 4 persone)*

500 gr ocra
2 pomodori freschi
1 petto di pollo
1 cipolla bianca
olio d'oliva
pepe, sale

Preparazione

1. Lavate e pulite le verdure, spuntando le cime dell'ocra. Tagliate la cipolla sottile, l'ocra a rondelle, i pomodori a pezzetti.

2. In una padella fate rosolare la cipolla, aggiungete il pollo tagliato a pezzetti e fate cuocere 10 minuti mescolando con un mestolo di legno. Aggiungete l'ocra e, dopo qualche minuto, i pomodori. Mescolate e aggiungete ½ bicchiere d'acqua. Aggiustate di sale, pepe e fate cuocere a fuoco medio per 10-15 minuti.

Potete accompagnare questo piatto con riso basmati o con la polenta. Qui la ricetta è con il pollo, in genere si fa con la carne di agnello e la cottura è più lunga. L'ocra è una verdura della famiglia delle malvacee, affine agli ibischi. È una pianta originaria dell'Africa tropicale e viene coltivata nei paesi caldi. Si trova facilmente nei negozi etnici e comincia ad essere coltivata anche negli orti in Trentino, a uso familiare, e in altre zone.

Con il pesce

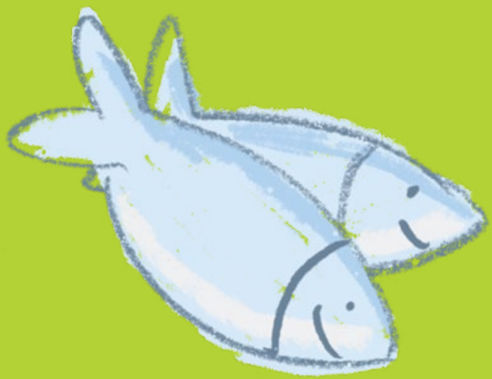

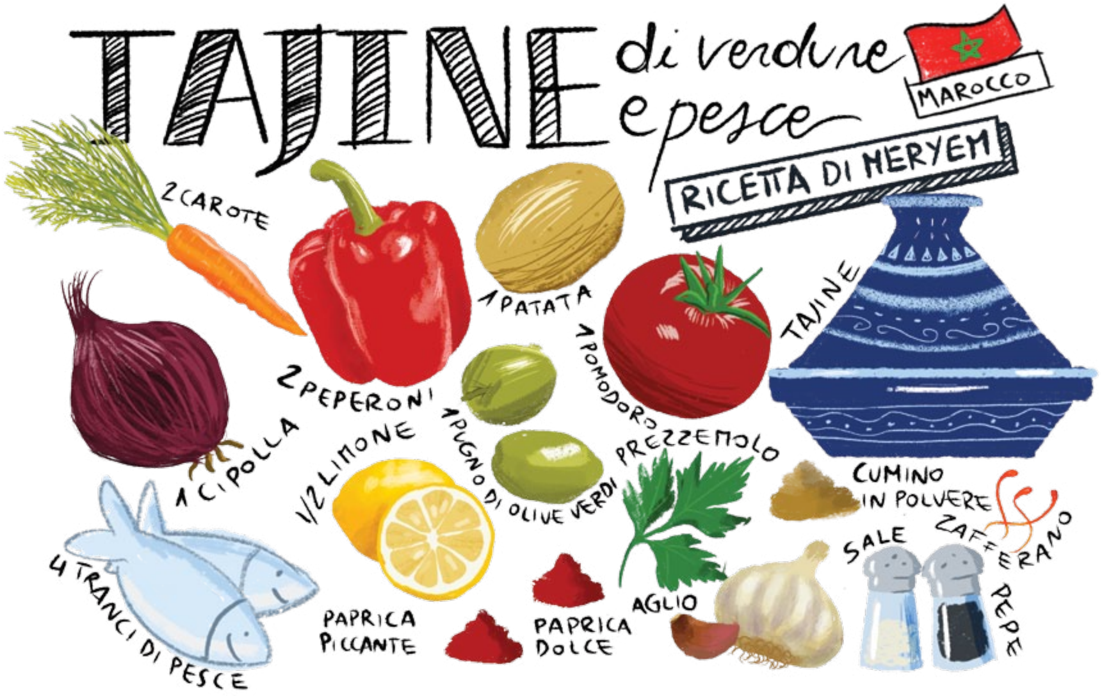

Ingredienti *(per 4 persone)*

2 carote
2 peperoni
1 patata
1 pomodoro
1 cipolla
4 tranci di pesce
(pesce spada, tonno o persico)
1 pugno di olive verdi
½ limone
paprica dolce
cumino in polvere
paprica piccante
zafferano
prezzemolo
1 spicchio d'aglio
pepe, sale

Preparazione

1. Pulite e lavate tutte le verdure. Tagliatele a rondelle e disponetele in cerchi concentrici sul piatto della tajine, a strati: prima le carote, poi le patate e le cipolle.

2. Lavate i tranci di pesce sotto l'acqua corrente, asciugateli con un foglio di carta assorbente e fateli marinare 10 minuti in una ciotola con: olio, sale, pepe, cumino, paprica dolce, paprica piccante, aglio e prezzemolo tritati, zafferano, il succo di ½ limone e un po' d'acqua.

3. Quando il pesce sarà marinato disponete i tranci nella tajine. Aggiungete le olive verdi, pomodoro e peperone a rondelle, qualche fetta di limone, mezzo bicchiere d'acqua e chiudete il coperchio. Fate cuocere a fiamma bassa per 30 minuti. Quando le verdure sono morbide il piatto è pronto.

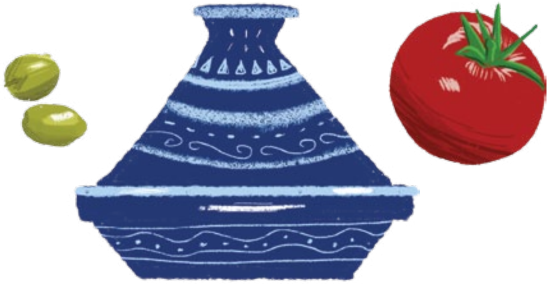

La tajine viene usata in tutto il Magreb e nei Paesi del Sud del Mediterraneo ma non troverete una ricetta uguale all'altra anche rimanendo in Marocco. Le similitudini non mancano - il tipo di cottura, la disposizione degli ingredienti sul piatto, l'uso delle spezie, la marinatura del pesce - poi ci sono le tradizioni di famiglia, la stagionalità, il tempo a disposizione, il gusto nella scelta delle spezie. Quando acquistate il pesce potete sceglierlo intero, se sapete pulirlo, oppure in tranci. Considerate che i tempi di cottura possono variare. Per preservare la tajine è consigliabile usare un frangifiamma e cuocere lentamente, a fiamma bassa.

BRANZINO al vapore

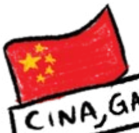

CINA, GANSU

RICETTA DI JIE

Ingredienti *(per 2 persone)*

2 branzini
4 cipollotti
zenzero fresco
salsa di soia
aceto
olio d'oliva
pepe bianco
sale

Preparazione

1. Squamate perfettamente i branzini con un coltello, sciacquateli sotto l'acqua corrente e asciugateli bene con la carta assorbente. Con un coltello affilato fate 3-4 incisioni trasversali sul dorso dei branzini, su entrambi i lati. In un contenitore mescolate sale e pepe: cospargete i branzini con questa miscela, massaggiate delicatamente per 1 minuto e fate riposare.

2. Nel frattempo pulite i cipollotti e separate la parte bianca da quella verde. Tagliate la parte bianca del cipollotto in verticale, in quattro parti, e tritate la parte verde finemente. Pulite lo zenzero e tagliatelo a fettine.

3. Mettete i branzini in un piatto fondo (distesi), copriteli con cipollotto e zenzero a fettine. Appoggiate il piatto nella vaporiera e fate cuocere i branzini a fuoco medio per 10-15 minuti, da quando l'acqua bolle.

4. Nel frattempo preparate la salsa: fate soffriggere alcune fettine di zenzero in 2 cucchiai d'olio e lasciate raffreddare. Versate l'olio in un contenitore, aggiungete la parte verde del cipollotto, qualche goccia di aceto e salsa di soia.

5. Quando il pesce è pronto, eliminate il liquido che si è formato durante la cottura, i cipollotti e lo zenzero in eccesso, e accompagnatelo con la salsa.

È una ricetta semplice e veloce, da fare al vapore. Per la cottura si usa la vaporiera e il pesce viene adagiato su un piatto o un foglio di carta da forno in modo che durante la cottura non perda le proprietà. Lo zenzero ha proprietà antiossidanti, di protezione delle cellule, e antinfiammatorie. Questo piatto si può accompagnare con riso bianco o patate lesse e verdure.

CHEVRETTES "Serge"

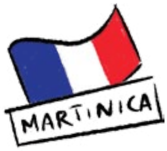

RICETTA DI JOSIANE

Ingredienti *(per 6 persone)*

1 kg di gamberoni o mazzancolle
3 pomodori maturi
1 cipolla bianca
400 ml di latte di cocco
1 ciuffo di prezzemolo
timo fresco o secco (facoltativo)
peperoncino piccante
3 spicchi d'aglio
olio di semi di arachidi
pepe, sale

Preparazione

1. Lavate e pulite tutte le verdure. Tagliate la cipolla sottile, i pomodori a pezzettini e tritate il prezzemolo. Lavate e pulite i gamberoni, eliminando testa, corazza e filamento sul dorso.

2. In una padella, mettete un cucchiaio d'olio e fate soffriggere cipolla e peperoncino per qualche minuto, aggiungendo il pomodoro alla fine. Unite i gamberoni, pepe, sale e continuate la cottura. Aggiungete timo, prezzemolo e aglio tritato. Mescolate qualche minuto e unite il latte di cocco facendo cuocere a fuoco medio per 15 minuti finché il liquido si restringe.

Potete accompagnare questo piatto con riso basmati o parboiled, oppure con manioca bollita. È un piatto della cultura culinaria creola e tradizionalmente si faceva con i gamberoni freschi di fiume.

SALMONE al CURRY

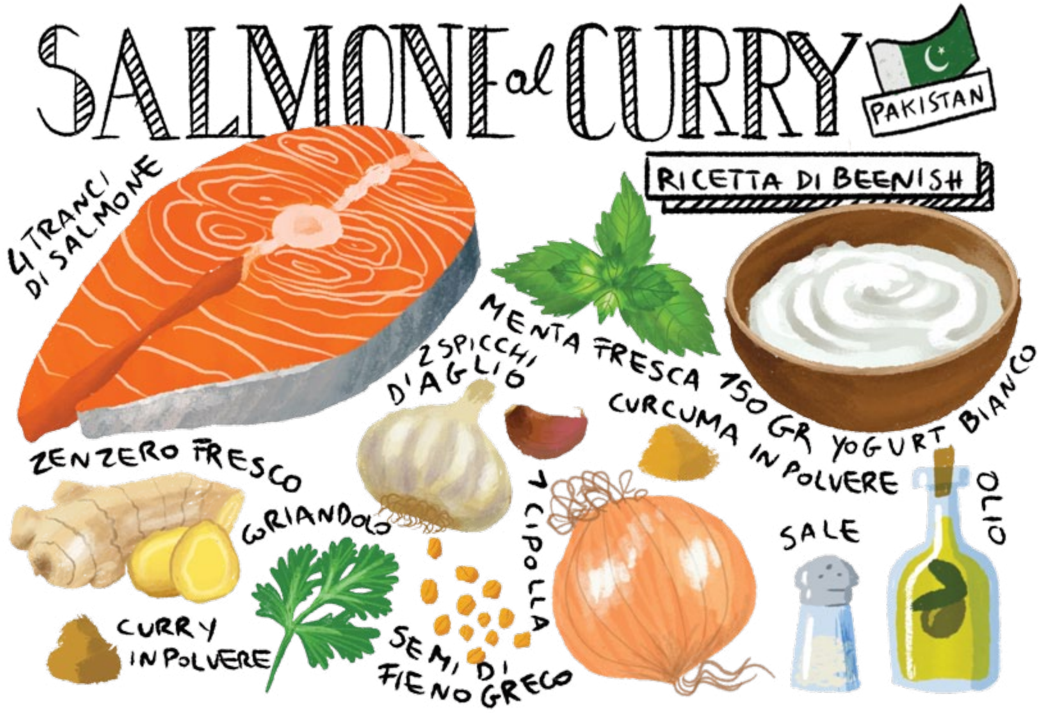

Ingredienti *(per 4 persone)*

4 tranci di salmone (circa 600 gr)
1 cipolla bianca
2 spicchi d'aglio
150 gr di yogurt bianco
zenzero fresco
menta fresca
coriandolo fresco
semi di fieno greco
curcuma in polvere
curry in polvere
olio d'oliva
sale

Preparazione

1. Lavate i tranci di salmone sotto l'acqua corrente. Asciugateli con un foglio di carta assorbente e fateli marinare, in frigorifero, per 20 minuti nello yogurt con le spezie: curry, curcuma e semi di fieno greco.

2. In una pentola capiente fate bollire 3 bicchieri d'acqua in cui avrete aggiunto due cucchiai d'olio d'oliva, gli spicchi d'aglio interi e qualche fettina di zenzero fresco. Quando l'olio viene a galla unite il pesce e fate cuocere per 10 minuti a fuoco medio. A fine cottura, scolate il pesce aiutandovi con un mestolo forato.

3. Servite il pesce con menta fresca e coriandolo tritati.

Con lo stesso procedimento potete cucinare merluzzo e orata. In base alla vostra abilità in cucina potete scegliere il pesce intero, pulirlo e sfilettarlo, oppure prendere filetti di pesce. Potete accompagnare il salmone al curry con riso basmati.

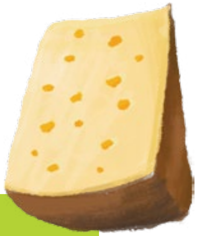

Conservare gli alimenti e ridurre gli sprechi

Quando facciamo la spesa è importante ridurre al minimo i tempi di trasporto per garantire la corretta conservazione degli alimenti (come latte, ricotta, carne, pesce) a temperatura controllata, in frigorifero, come indicato sull'etichetta.
Per gli alimenti freschi si raccomanda di non superare la data di scadenza (da consumare entro) poiché è il limite entro cui viene garantita la salubrità del prodotto, diversamente dagli alimenti a lunga conservazione (da consumare preferibilmente entro).
Per la conservazione in frigorifero è opportuno osservare alcune indicazioni: la temperatura interna va mantenuta intorno ai 4-5 °C, tenendo conto che in genere la parte più fredda è in basso (sopra il cassetto per le verdure) mentre lo sportello è la parte più calda.
Si raccomanda di riporre carne e pesce nelle mensole in basso; uova e formaggi nella parte centrale; verdura e frutta nel cassetto in basso.
Per i cibi cotti, prima di metterli in frigorifero occorre lasciarli raffreddare e conservarli in contenitori puliti e chiusi, anche per evitare eventuali contaminazioni con gli alimenti crudi.
Inoltre, meglio evitare di riempire il frigorifero con scorte abbondanti sia per garantire il circolo dell'aria fredda all'interno che per ridurre gli sprechi.
Secondo la FAO (Food and Agriculture Organization), nel mondo, circa il 30% dei prodotti alimentari viene perso o buttato nella spazzatura.
Gli sprechi avvengono durante la trasformazione industriale (39%), la distribuzione (5%), i consumi fuori casa (14%) e in casa (42%). Se le politiche possono fare molto (in Europa l'obiettivo è di dimezzare i rifiuti alimentari entro il 2025), ciascuno può contribuire alla riduzione degli sprechi a partire dalle proprie abitudini. [FONTE: Decalogo sicurezza nel frigorifero, Ministero della Salute; European Commission preparatory study on food waste, FAO, 2010; Risoluzione del Parlamento Europeo su come evitare lo spreco di alimenti, 2012]

POLPETTINE di sardine

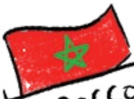

MAROCCO

RICETTA DI REDOUANE

Ingredienti *(per 4 persone)*

500 gr di sardine
40 gr di riso
2 tazze di salsa di pomodoro
1 cipolla bianca
2 spicchi d'aglio
prezzemolo fresco
coriandolo fresco
zenzero macinato
peperoncino dolce
pepe nero macinato
olio d'oliva
sale

Preparazione

1. Sciacquate il riso un paio di volte sotto l'acqua fredda per togliere l'amido. Fate sbollentare il riso in una pentola con l'acqua e scolatelo a metà cottura.

2. Pulite e squamate le sardine. Lavatele sotto l'acqua corrente, appoggiatele su un tagliere inclinato o un piatto per farle sgocciolare. Tritate le sardine nel mixer con prezzemolo, coriandolo, zenzero, pepe, peperoncino, aglio (a piacere) e un cucchiaio d'olio.

3. In una ciotola unite il riso e l'impasto di sardine amalgamando bene con una forchetta. Quando la consistenza è giusta, fate delle polpette con le mani premendo bene in modo che durante la cottura non si disfino. Disponete man mano le polpette su un foglio di carta da forno.

4. A parte, in una padella fate soffriggere la cipolla tagliata sottile con un cucchiaio d'olio e l'aglio. Aggiungete la salsa di pomodoro, ½ bicchiere d'acqua e fate cuocere a fuoco medio per 5 minuti. Abbassate la fiamma, adagiate le polpette nel sugo, coprite con un coperchio e fate cuocere a fiamma bassa per 20 minuti.

5. Servite le polpette calde accompagnate da cous cous, pane o riso.

Per la cottura si può usare una pentola con il fondo spesso oppure la tajine, perfetta per una cottura lenta e naturale. Potete dosare le spezie in base ai gusti. Prezzemolo e coriandolo vanno usati preferibilmente freschi. Al posto della salsa di pomodoro potete usare due pomodori maturi. In alternativa al riso potete usare 3 cucchiai di farina di riso, utile anche per infarinare le mani e fare le polpette.

Zuppe
e vellutate

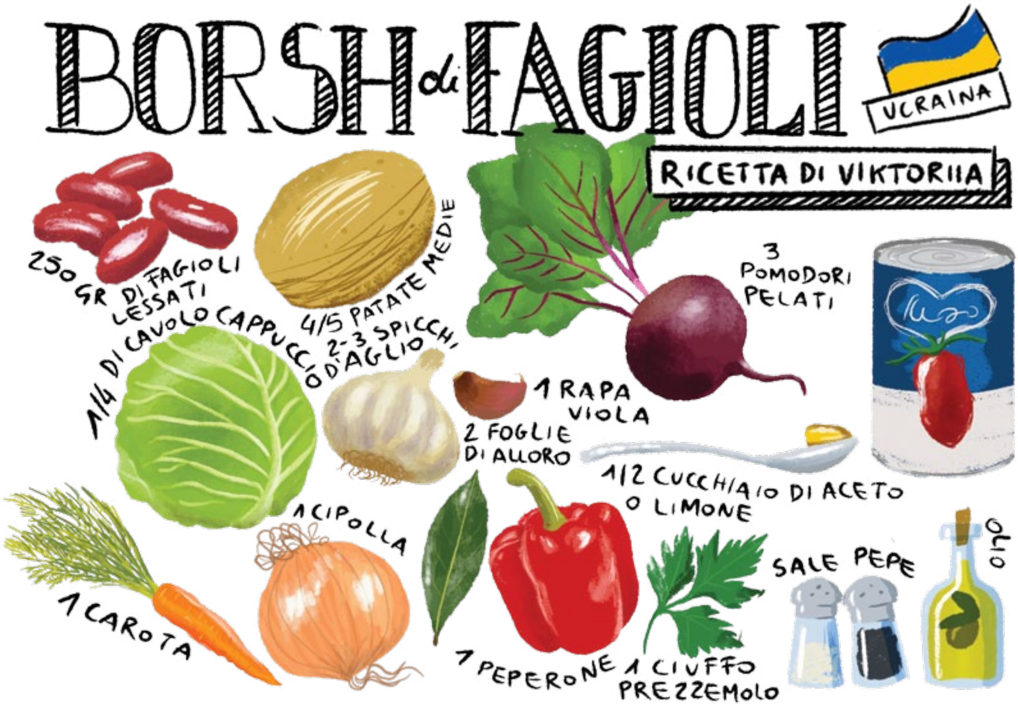

Ingredienti *(per 6 persone)*

250 gr di fagioli lessati
4/5 patate medie
1 cipolla bianca
1 carota
1 peperone
1 rapa viola
¼ di cavolo cappuccio verde
3 pomodori pelati
2-3 spicchi d'aglio
2 foglie di alloro
1 ciuffo di prezzemolo
½ cucchiaio di aceto o limone
olio d'oliva
pepe, sale

Preparazione

1. Pulite e lavate tutte le verdure. Tagliate le patate, le carote, i pomodori e i peperoni a pezzetti piccoli, le rape e il cavolo cappuccio a strisce sottili, la cipolla tritata finemente.

2. In una pentola capiente fate cuocere le patate coperte d'acqua, con un po' di sale e alloro.

3. A parte, in una padella antiaderente, fate soffriggere cipolla, aglio, carote e rape con un cucchiaio d'olio. Mescolate con un mestolo di legno

qualche minuto e aggiungete peperone e pomodori. Aggiustate di sale e fate cuocere a fiamma bassa. Se i pomodori sono dolci potete aggiungere aceto o succo di limone, a piacere.

4. Quando il soffritto è morbido unitelo alle patate. Aggiungete i fagioli lessati (senza acqua di cottura), il cavolo cappuccio e fate cuocere a fiamma media. Se serve potete aggiungere qualche mestolo di acqua bollente. Quando le verdure sono pronte, spegnete il fuoco e fate riposare per 15 minuti. Alla fine aggiungete prezzemolo tritato e pepe.

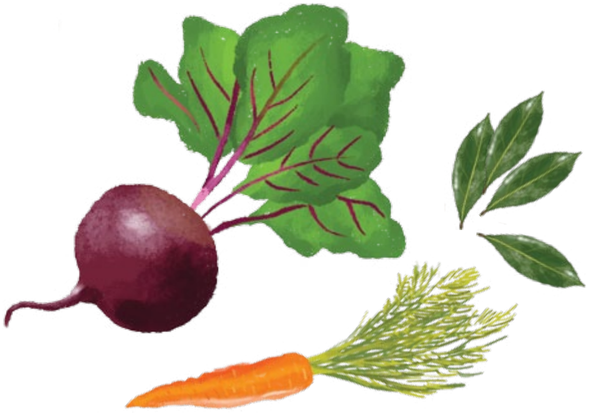

Per questa ricetta potete usare fagioli secchi, mettendoli in ammollo la sera prima e facendoli cuocere a parte, oppure fagioli già lessati controllando l'etichetta in modo da scegliere un prodotto con poco sale e conservanti. Se usate fagioli pronti, scolateli e sciacquateli prima di unirli agli altri ingredienti. I pomodori possono essere freschi oppure potete usare pomodori pelati pronti. A fine cottura, prima di servire il borsh potete aggiungere un po' di panna acida o un cucchiaio di yogurt greco a piacere.

HARIRA

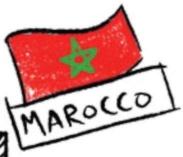

RICETTA DI HANANE

Ingredienti *(per 4/6 persone)*

1,5 kg di pomodori
2 cipolle bianche
1 ciotola di ceci secchi
1 ciotola di lenticchie
1 ciuffo di prezzemolo
1 ciuffo di coriandolo fresco
zenzero fresco
olio di semi
pepe, sale

Preparazione

1. Mettete in ammollo i ceci la sera prima e sciacquateli prima di cucinarli. Per le lenticchie non serve l'ammollo, hanno tempi di cottura più rapidi, basta sciacquarle sotto l'acqua fredda.

2. Pulite e lavate le verdure. Tagliate i pomodori a pezzi grossi, frullateli con il mixer e passateli in un colino in modo da fare una salsa vellutata. Tritate le cipolle con il mixer e preparate il prezzemolo e il coriandolo tagliati sottili.

3. Per la cottura potete usare la pentola a pressione in modo da ridurre i tempi. Mettete un cucchiaio d'olio nella pentola, aggiungete cipolla, ceci, lenticchie, pomodoro, spezie e coprite d'acqua. Fate cuocere 15 minuti. A fine cottura assaggiate, aggiustate di sale e spolverate con coriandolo e prezzemolo.

4. Alla fine, per addensare la zuppa, potete sciogliere un cucchiaio di farina con acqua tiepida, poco sale, un po' di aceto bianco in una ciotola. Sbattere bene con una frusta gli ingredienti per evitare che si formino grumi e aggiungere il liquido alla zuppa, facendola cuocere ancora qualche minuto.

L'harira è una zuppa tradizionale marocchina. Si mangia durante l'anno, in occasione delle feste e durante il Ramadan. Nella ricetta tradizionale si aggiunge un pezzo di carne di montone e verso fine cottura un cucchiaio di nsemen (simile al burro, lo trovate nei negozi etnici).

ROSÓŁ

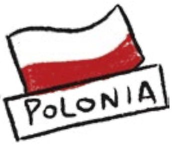

RICETTA DI MICHAŁ

Ingredienti *(per 6 persone)*

½ pollo
2 l di acqua
1 carota
1 cipolla
½ sedano rapa
1 gambo di sedano
½ porro
1 radice di prezzemolo
2 foglie di alloro
pimento in grani
pepe nero in grani

Preparazione

1. Lavate il pollo sotto l'acqua corrente. Lavate, sbucciate e preparate le verdure per il brodo: potete tagliare il porro, le carote e la cipolla a metà, il sedano e la radice di prezzemolo a pezzi più piccoli.

2. Mettete il pollo e tutte le verdure in una pentola capiente, coprite d'acqua, aggiungete alloro, pimento e pepe nero. Fate cuocere a fiamma viva finché il brodo inizia a bollire. Poi abbassate, coprite con il coperchio e proseguite la cottura al minimo, facendo andare il brodo lentamente per un'ora e mezza almeno.

Il brodo è uno dei piatti tipici della cucina casalinga polacca. Si mangia quasi ogni giorno ed è il piatto principale, quello con cui si apre il pranzo. In genere, dato che un buon brodo può cuocere anche 3-5 ore, si prepara in quantità abbondante e si conserva in frigorifero o si congela. Un buon brodo è la base per la preparazione di altri piatti della cultura culinaria polacca, come la vellutata di cipolle, la zuppa di patate e la minestra d'aglio.

ZUPPA di VERDURE

TRANSILVANIA

RICETTA DI EMILIA

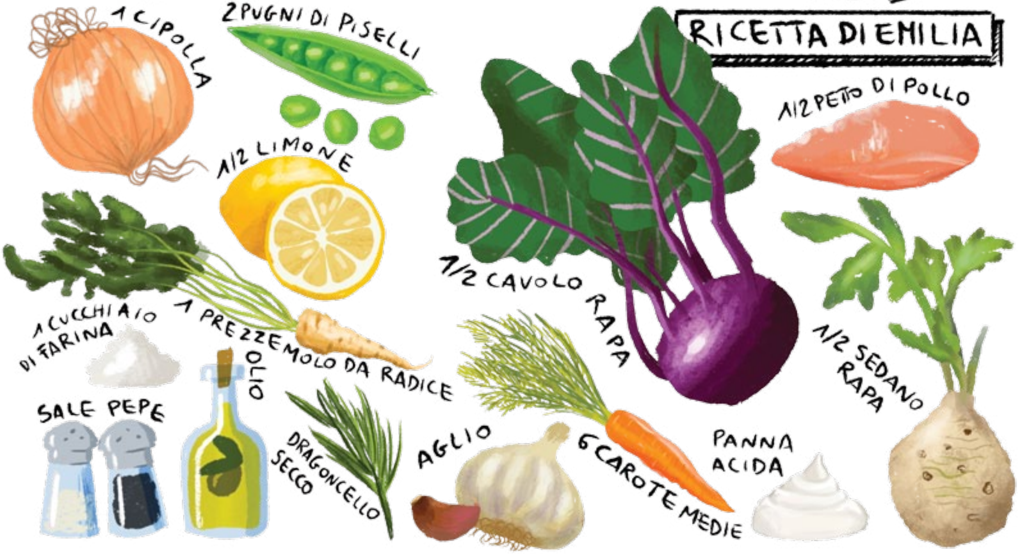

Ingredienti *(per 4 persone)*

1 cipolla
6 carote medie
½ sedano rapa
½ cavolo rapa (kohlrabi)
2 pugni di piselli
1 prezzemolo da radice
½ petto di pollo o tacchino
½ limone
dragoncello secco
1 cucchiaio di farina
olio d’oliva
panna acida
1 spicchio d’aglio
pepe, sale

Preparazione

1. Pulite tutte le verdure e tagliatele a pezzetti piccoli. Tagliate a pezzetti il petto di pollo e mettetelo in una ciotolina, separato dal resto.

2. Prendete una pentola capiente, con un cucchiaio d’olio d’oliva sul fondo e fate rosolare la cipolla. Aggiungete tutte le verdure, meno i piselli poiché hanno bisogno di meno tempo per cuocere. Fate andare a fuoco basso per qualche minuto, aggiungete il pollo e girate con un mestolo di legno. Aggiungete l’aglio tritato o schiacciato.

3. Versate abbondante acqua nella pentola fino a coprire tutti gli ingredienti. Per la quantità d'acqua potete regolarvi in base alla consistenza che volete dare alla zuppa. Aggiungete un cucchiaino di dragoncello secco e una spruzzata di limone. Continuate la cottura a fuoco medio per 35 minuti.

4. Quando la zuppa è quasi pronta, potete aggiungere la panna acida. Mescolate, fate cuocere ancora qualche minuto e servite la zuppa calda.

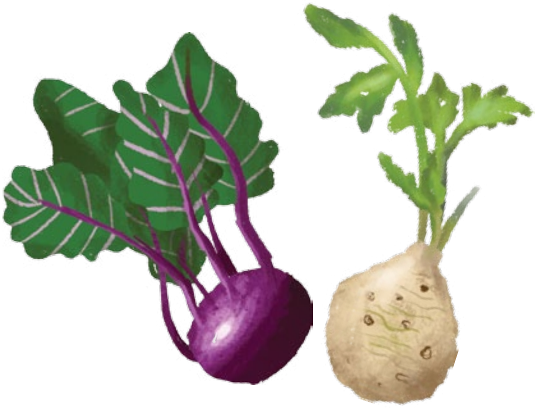

Questa zuppa ungherese è fatta di verdure, in particolare radici: carote, sedano rapa, prezzemolo da radice. Un tempo, negli orti venivano coltivati molti ortaggi e radici resistenti al freddo. Mentre il sedano rapa è noto e coltivato anche nelle nostre zone, il cavolo rapa viene dal nord Europa e si è diffuso di recente, usato prevalentemente crudo nelle insalate. Il prezzemolo da radice fa parte della tradizione culinaria del nord Europa ed è ancora difficile da trovare (in questa ricetta si usa la radice, non le foglie). Il dragoncello è un'erba aromatica, più noto nella cucina toscana e molto usato nel nord Europa sia fresco che essiccato, oltre che conservato sott'aceto.

SCAVLEVA ZUPA

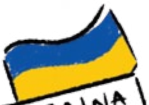

UCRAINA

RICETTA DI HALYNA

Ingredienti *(per 4 persone)*

5 patate
2 carote
1 gambo di sedano
1 cipolla media
2 pugni di acetosa
(Rumex acetosa)
1 spicchio d'aglio
3 uova
aceto
sale

Preparazione

1. Pulite tutte le verdure e tagliatele a pezzetti. Mettete le verdure in una pentola, aggiungete uno spicchio d'aglio e coprite d'acqua. Portate a bollore e proseguite la cottura a fuoco medio per 20 minuti, aggiungendo un po' di sale (regolatevi in base alla quantità d'acqua).

2. Nel frattempo lavate le foglie di acetosa, tagliatele sottili, aggiungetele alla zuppa e fate cuocere ancora 10 minuti.

3. A parte, in un pentolino, preparate le uova sode: fate bollire l'acqua con una goccia di aceto, immergete le uova, fatele cuocere per 8-10 minuti e sbucciatele.

4. Servite la zuppa calda con qualche pezzetto di uovo sodo all'ultimo momento.

L'acetosa cresce spontanea sui bordi dei prati, in Ucraina come in Trentino. Raccoglietela fresca, quando le foglie sono tenere: in primavera fino all'inizio dell'autunno. La ricetta tradizionale prevede di aggiungere panna acida a fine cottura ma, anche senza, è buonissima e più leggera.

VELLUTATA di ortiche

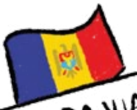

MOLDAVIA

RICETTA DI VERONICA

Ingredienti *(per 4 persone)*

2 pugni di foglie di ortiche fresche
2 patate medie
1 cipolla bianca
1 spicchio d'aglio
olio d'oliva
pepe nero

Preparazione

1. Pulite e lavate le verdure. Tagliate la cipolla sottile e le patate a dadini piccoli. Le foglie di ortiche vanno messe in ammollo qualche minuto, sciacquate e tagliate come fossero spinaci. L'unica accortezza è fare attenzione alle mani perché le foglie pizzicano.

2. In una pentola capiente mettete un cucchiaio d'olio, le patate e coprite d'acqua. Fate cuocere a fuoco medio per 10 minuti. Aggiungete le ortiche, aggiustate di sale e continuate la cottura. Quando le verdure sono cotte passatele con il mixer e frullate bene.

3. A parte, in una padella fate soffriggere la cipolla tagliata sottile con un cucchiaio d'olio e uno spicchio d'aglio.

4. Quando la vellutata di ortiche è pronta, servitela con un cucchiaio di cipolla, pepe nero e olio d'oliva alla fine.

Questa zuppa fresca si può fare in primavera o in autunno quando le foglie di ortica sono tenere. A fine cottura, potete aggiungere qualche cucchiaio di farro (fatto cuocere a parte in abbondante acqua) per rendere più consistente la vellutata. Se preferite, al posto delle patate potete usare fagioli borlotti.

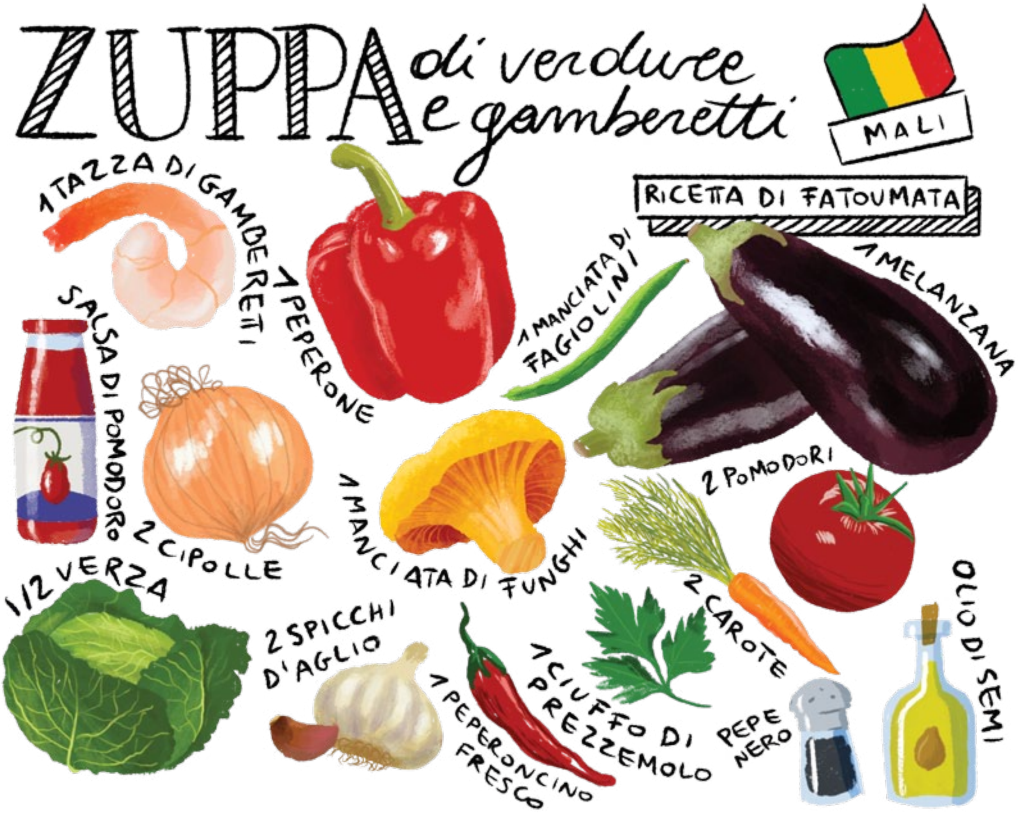

Ingredienti *(per 6 persone)*

1 tazza di gamberetti
2 cipolle
2 pomodori
2 carote
1 peperone
1 melanzana piccola
1 manciata di fagiolini verdi
1 manciata di funghi
½ verza (non troppo grande)
1 bicchiere di salsa di pomodoro
1 peperoncino piccante fresco
1 ciuffo di prezzemolo
2 spicchi d’aglio
pepe nero
olio di semi

Preparazione

1. Pulite i gamberetti, sbucciateli e lavateli sotto l’acqua corrente. Pulite e lavate tutte le verdure, tagliatele a pezzetti e tenetele separate.

2. In una pentola capiente, mettete un cucchiaio d’olio e fate rosolare a fiamma bassa un trito di cipolla, pomodoro, aglio, peperoncino e prezzemolo. Aggiungete la salsa di pomodoro, mescolate e fate cuocere qualche minuto. Togliete la pentola dal fuoco e versate all’interno 1 litro d’acqua circa. Quando bolle, aggiungete tutte le verdure tagliate a pezzetti e fate

cuocere per 40 minuti circa a fiamma media. A fine cottura regolate di sale, pepe e aggiungete i gamberetti. Mescolate e fate cuocere ancora 10 minuti.

Si può accompagnare questa zuppa con riso, pane oppure cous cous che in Mali è a semola fine. I gamberetti andrebbero fatti insaporire nel trito di verdure, all'inizio, per qualche minuto. Poi tolti, lasciati da parte e aggiunti a fine cottura. Per praticità potete aggiungere i gamberetti alla fine e proseguire la cottura ancora 10 minuti. La quantità d'acqua può variare in base ai gusti, in modo da ottenere una zuppa più densa oppure liquida.

I bambini imparano a mangiare bene in famiglia

Molte ricette sono state raccolte nell'ambulatorio pediatrico della dott.ssa Monica Ghezzi, a Gardolo, in una zona di Trento con un'alta percentuale di famiglie immigrate. Ecco qualche suggerimento a partire dalla sua esperienza. La nascita di un bambino è un momento in cui la coppia genitoriale è particolarmente recettiva verso consigli che riguardano lo stile di vita.

Fino al sesto mese i bambini si alimentano esclusivamente con latte materno (o con latte artificiale quando necessario o preferito dalla mamma), poi si affronta il tema dell'alimentazione complementare, con l'introduzione nella dieta del bambino di alimenti solidi diversi dal latte.

Per le famiglie è un buon momento per acquisire informazioni di tipo nutrizionale e correggere eventuali errori alimentari. Ha poco significato seguire una corretta alimentazione nei primi mesi di vita del bambino se poi si abbandona questa attenzione ai principi di sana alimentazione appena è diventato più grande.

Offrire di consuetudine dolci ai bambini, per quanto possa essere vissuto dai genitori come un gesto di affetto, è un comportamento da evitare perché può provocare problemi ai denti e al metabolismo. Un'altra competenza importante per le famiglie è quella di imparare a fare la spesa e scegliere gli alimenti giusti da portare a tavola.

Per una corretta educazione al gusto, oltre ad alimentare correttamente un bambino nei primi anni di vita, occorre far sì che impari ad autoregolarsi e a scegliere gli alimenti giusti per sé. Tutte le tradizioni alimentari forniscono ricette adatte a una sana alimentazione, se ben equilibrate e nella giusta quantità. Non sempre è facile per chi vive lontano dal proprio paese di origine riprodurre le ricette a cui si è legati, ma con qualche suggerimento è possibile mangiare piatti della tradizione, seppure rivisitati con ingredienti locali.

ZUPPA di PATATE

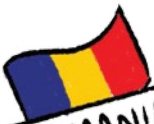

ROMANIA

RICETTA DI DIANA

Ingredienti *(per 4 persone)*

1 cipolla bianca
1 carota
1 peperone rosso
1 gambo di sedano
3 patate medie
1 ciuffo di prezzemolo tritato
1 manciata di timo tritato
¼ di succo di limone (o bors)
sale e pepe

Preparazione

1. Pulite e lavate tutte le verdure. Tagliate a pezzetti piccoli carota, peperone, sedano e cipolla; le patate a pezzetti di media grandezza. Lavate il prezzemolo e tritatelo sottile.

2. In una pentola capiente, mettete tutte le verdure, coprite d'acqua e lasciate cuocere per 20 minuti a fuoco medio. A fine cottura aggiungete il bors o il succo di limone, prezzemolo e timo. Regolate di sale, pepe e proseguite la cottura per 5 minuti ancora.

3. Servite la zuppa di patate calda o tiepida.

Alla zuppa si può aggiungere un pugno di pastina o tagliolini freschi verso fine cottura, facendoli cuocere per 6-8 minuti. Inoltre, se vi piace, potete aggiungere poca panna fresca prima di mettere nei piatti. Il bors è un succo acidulo ricavato dalla fermentazione di varie crusche di cereali e si può trovare nei supermercati rumeni. In alternativa potete usare succo di limone.

ZUPPA di POLLO

ROMANIA

RICETTA DI DIANA

Ingredienti *(per 4 persone)*

1 cipolla bianca media
2 carote
1 gambo di sedano
2-3 pomodorini
1 carota
400/500 gr di pollo
½ bicchiere di salsa di pomodoro
2 cucchiai di aceto bianco
1 ciuffo di prezzemolo tritato
olio d'oliva
sale

Preparazione

1. Lessate il pollo coperto d'acqua, con un pizzico di sale, per 10 minuti. Quando il pollo è pronto toglietelo dalla pentola con una schiumarola e tagliatelo a pezzetti o listarelle. Nel frattempo pulite, lavate e tagliate le verdure a pezzetti piccoli. Mettete il pollo e le verdure in una pentola (lasciando da parte cipolla e pomodorini) e coprite appena d'acqua.

2. A parte, in una padella fate rosolare la cipolla, aggiungete i pomodorini e la salsa di pomodoro. Fate cuocere per qualche minuto in modo che si addensi e versate la salsa nella pentola con le verdure e il pollo. Mescolate, aggiustate di sale e fate cuocere la zuppa per 40 minuti circa.

3. Quando le verdure sono morbide e mancano pochi minuti a fine cottura, abbassate la fiamma e aggiungete l'aceto. Spegnete il fuoco, lasciate intiepidire e aggiungete abbondante prezzemolo tritato.

Potete rendere più consistente la zuppa con un pugno di tagliatelle di riso o pasta spezzata, lasciando cuocere per 6-8 minuti ancora. Nelle zuppe rumene non manca mai la panna fresca o la panna acida. Se vi piace potete aggiungerne poca alla fine insieme a una punta di peperoncino. Anche senza panna, la zuppa è buona e più leggera.

Spuntini e merende

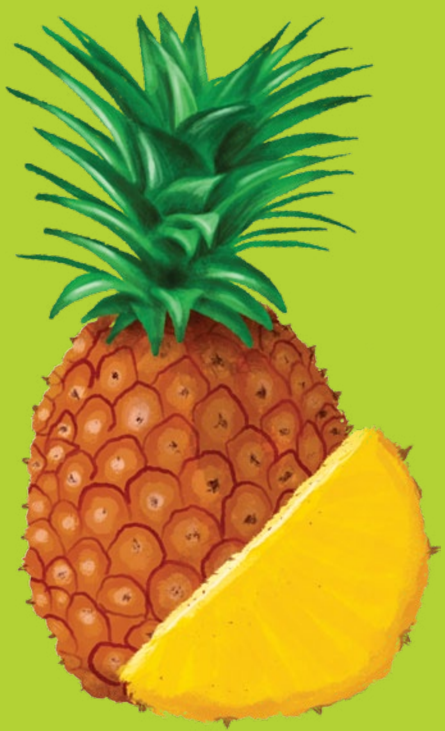

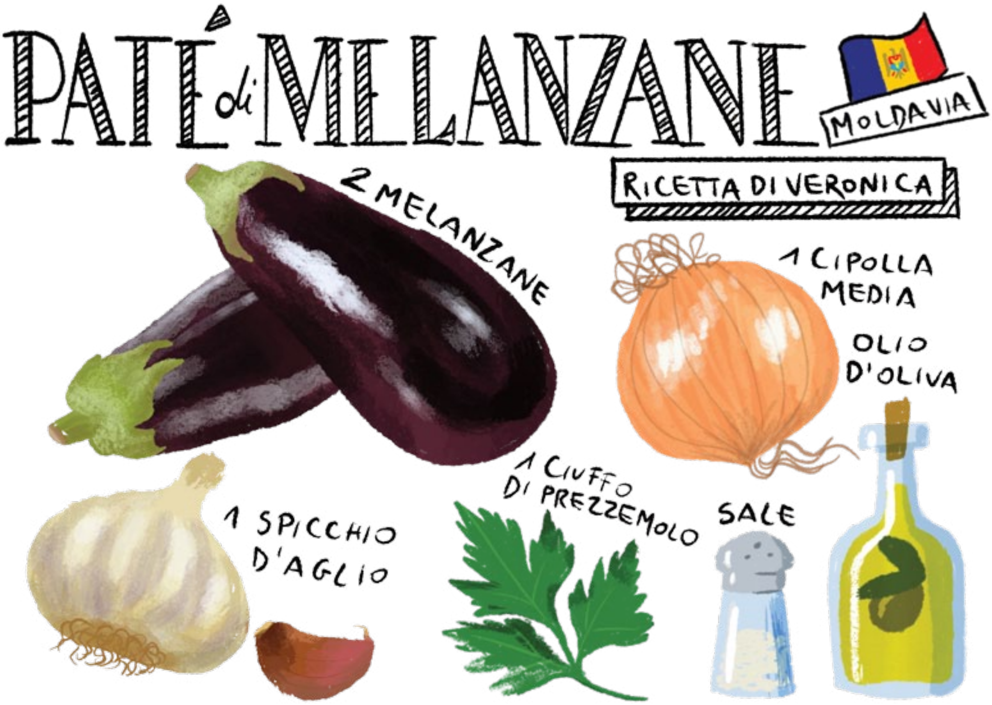

Ingredienti *(per 4 persone)*

2 melanzane
1 cipolla media
1 spicchio d’aglio
1 ciuffo di prezzemolo
olio d’oliva
sale

Preparazione

1. Lavate le melanzane e fate qualche incisione con il coltello lasciandole intere. Mettete le melanzane in una teglia e arrostitele al forno per circa 1 ora a temperatura elevata. La buccia diventerà scura mentre la melanzana cuoce all’interno.

2. Togliete le melanzane dal forno, sbucciatele e raccogliete la polpa con un cucchiaio. Lasciate scolare la polpa qualche minuto, strizzatela e tagliatela a pezzetti.

3. A parte, in una padella fate un soffritto di cipolla tagliata sottile con un cucchiaio d’olio, l’aglio intero schiacciato e poco sale. Aggiungete la polpa di melanzana e fate rosolare mescolando con un mestolo di legno. Cuocete qualche minuto a fuoco medio continuando a girare.

4. Quando è pronto, servite il paté di melanzane in una ciotola con un cucchiaio d'olio e prezzemolo tritato.

Il paté di melanzane si può mangiare con dei crostini di pane. A seconda dei gusti potete rendere questa crema più o meno saporita dosando la quantità di aglio e potete aggiungere peperoncino a piacere. Tradizionalmente è una crema che si abbina bene al vino rosso, facendo attenzione a non superare le quantità considerate a basso rischio: 1 bicchiere al giorno per le donne, 2 bicchieri al giorno per gli uomini.

Mentre il pane lievita c'è il tempo di costruire relazioni

Cosa ci vuole a fare il pane? Al Forno Sociale Migola di Canova di Gardolo, dove abbiamo raccolto alcune ricette contenute in questo libro, funziona così: porti lievito, farina e cominci a impastare. Non serve essere capaci, basta mettersi vicino a qualcuno che ha più esperienza. Chiedere, osservare, farsi aiutare.

Il pane è fatto di pochi ingredienti semplici, alla portata di tutti e contemporaneamente è frutto di maestrie complesse, trucchi segreti e ricette tramandate. Se ne potrebbe parlare per ore: appartiene a moltissime tradizioni culinarie, lo possono impastare mani grandi e mani piccole, esperte o meno.

Al Forno Sociale arrivi e sullo stesso tavolo trovi qualcuno che mescola farina bianca, di semola, integrale. C'è chi aggiunge qualche cucchiaino di curcuma all'impasto, chi preferisce semi di girasole o papavero. Uno degli ingredienti più importanti del pane è il tempo: quando l'impasto è morbido, lavorato abbastanza, lo si fa riposare. E mentre lievita c'è il tempo di scambiarsi una ricetta, conoscere qualcuno, condividere un pensiero e semplicemente stare insieme. Prima di cuocere, il pane deve avere una forma: ciascuno ha la sua tecnica, non ci sarà un pane uguale all'altro. Poi prendi una teglia, la copri con un foglio di carta da forno, appoggi le forme di pane e inforni. Quando il pane è pronto si assaggia: un pezzetto di pane, olio d'oliva e una punta di peperoncino fresco piccante. Il pane condiviso ha tutto un altro sapore.

Il Forno Sociale Migola di Canova di Gardolo è un luogo aperto a tutti: senza vincoli di età, lingua, competenze, provenienza. Nato dal pensiero e dall'energia dell'Associazione Carpe Diem e dell'Associazione di volontariato Germogli, dal 2015 impasta farina, lievito e relazioni.

ALOO PARATHA

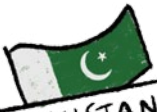

PAKISTAN

RICETTA DI BEENISH

Ingredienti *(per 6 paratha)*

500 gr di farina integrale
2 patate medie
1 cipolla bianca
semi di cumino
coriandolo in polvere
menta in polvere
olio d'oliva
sale

Preparazione

1. Pelate le patate e fatele bollire coperte d'acqua. Quando sono pronte, scolate le patate e schiacciatele bene. Aggiungete sale, coriandolo e menta.

2. In una padella fate soffriggere la cipolla tagliata sottile con due cucchiai d'olio. Quando è ben rosolata, aggiungete le patate e amalgamate finché risulta un composto cremoso.

3. A parte, in una ciotola preparate l'impasto del pane con farina integrale e acqua. Lavorate con le mani, aggiungete semi di cumino, olio, sale e continuate a impastare finché diventa morbido. Fate riposare per 20 minuti l'impasto coperto con uno strofinaccio.

4. Prendete l'impasto e dividetelo in 12 parti. Su un piano di legno o sul tavolo spolverato di farina, stendete una alla volta le palline con il mattarello per farne dei dischi sottili.

5. Prendete 6 dischi e, su ciascuno, mettete un cucchiaio di patate e cipolla al centro. Copriteli uno alla volta con un altro disco e stendete di nuovo l'impasto con il mattarello. In questo modo il composto di patate e cipolla viene incorporato nel pane.

6. Fate cuocere il pane su entrambi i lati in una padella calda. Man mano che i dischi sono pronti appoggiateli in verticale coperti da uno strofinaccio.

In Pakistan ci sono molti tipi di pane. Il più semplice è il chapati, fatto con farina integrale e acqua, cotto sul fuoco in una padella larga con il fondo pesante. Il paratha è fatto come il chapati con l'aggiunta di olio. Per fare l'aloo paratha si aggiungono anche le patate e la cipolla, oltre all'olio. Ci sono poi altre varianti che prevedono l'aggiunta di spinaci e altre verdure cotte. In tavola non manca mai il pane che accompagna moltissimi piatti della cucina indiana e pakistana. Questo pane morbido si accompagna bene con una salsa di yogurt bianco, cumino, coriandolo in polvere e un pizzico di sale.

AREPAS

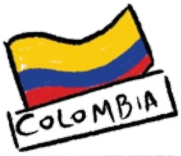

RICETTA DI MARIELA

Ingredienti *(per 5 arepas)*

1 tazza di farina di mais bianco (maiz blanco)
1 tazza e ½ di acqua tiepida
1 pizzico di sale
1 pizzico di zucchero di canna
olio di semi

Preparazione

1. Versate l'acqua in una ciotola, aggiungete un pizzico di sale e la farina di mais. Con un mestolo di legno mischiate bene gli ingredienti e fate riposare l'impasto, coperto con un canovaccio, per 10 minuti. Lavorate di nuovo l'impasto con le mani finché diventa sodo e morbido.

2. Prendete delle porzione uguali di impasto e fate delle palline. Schiacciatele una a una per fare dei dischi larghi circa 10 cm di diametro, spessi 1,5 cm e appoggiateli su una carta da forno.

3. Prendete una padella antiaderente, mettete un cucchiaio d'olio, scaldatela a fiamma bassa e cuocete le arepas, poche per volta, su entrambi i lati. Man mano che sono pronte, appoggiatele in verticale su uno strofinaccio e tenetele al caldo.

4. Potete mangiare le arepas a colazione con la marmellata o come il pane, insieme alle verdure o al formaggio.

Le arepas sono un pane tipico della Colombia e del Venezuela. Non c'è lievito, sono fatte di farina di mais che potete trovare nei negozi etnici o nel reparto delle farine nei negozi ben forniti. Le arepas, un po' come le piadine, si possono mangiare con il formaggio oppure con le verdure (mais, carota, sedano stufati) o con il pollo.

MAST-O-CHIAR

IRAN

RICETTA DI PIROUS

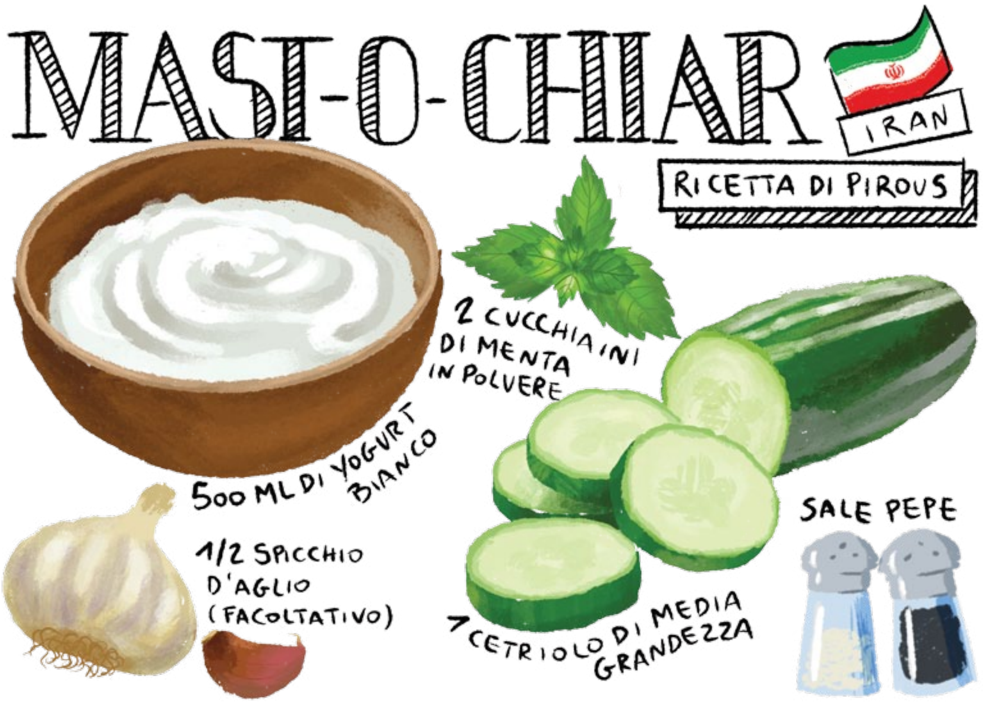

Ingredienti *(per 3 persone)*

500 ml di yogurt bianco
1 cetriolo di media grandezza
2 cucchiaini di menta in polvere
½ spicchio di aglio (facoltativo)
pepe, sale

Preparazione

1. Potete sbucciare il cetriolo, tagliando via anche le due estremità, oppure lavarlo bene e lasciare la buccia. Tagliate il cetriolo a dadini abbastanza piccoli.

2. Mettete gli ingredienti in una ciotola: cetriolo, yogurt, menta, sale e pepe. Potete aggiungere un trito d’aglio se vi piace. Mescolate e amalgamate bene tutto e servite. Se avete tempo di lasciarlo raffreddare un’ora in frigorifero diventa più buono e saporito. In frigo si mantiene almeno 2 giorni, ma di solito non avanza mai niente.

Mast-o-chiar è in lingua farsi, in italiano significa semplicemente "yogurt e cetriolo" e potete usare anche lo yogurt di soia. Di solito piace molto ai bambini. Va bene come merenda nei pomeriggi estivi oppure come aperitivo. Va benissimo anche come condimento a un crostino o con una fetta di pane abbrustolito. E diventa una bruschetta italo-persiana.

Oltre a mangiare sano è importante muoversi

Gli effetti positivi dello sport e del movimento quotidiano sono ormai dimostrati ma nonostante questo la società moderna è sempre più sedentaria, sempre più curva davanti a computer e schermi, sempre più seduta in macchina o al lavoro, sempre più fisicamente ferma. Questa sedentarietà, ormai presente anche nei bambini, è un fattore di rischio per diverse malattie croniche (tra cui ipertensione, malattie cardiovascolari, tumore al seno e colon retto, diabete). Fare movimento non è più quindi un consiglio ma una necessità che dovrebbe entrare a far parte della vita quotidiana di tutti. Molte persone si scoraggiano pensando al movimento e allo sport come a qualcosa di faticoso, per il quale occorre avere molto tempo e dedicare molte energie. Tuttavia non bisogna essere atleti per fare attività fisica, bastano 30 minuti di attività moderata al giorno. Andare a piedi o in bicicletta al lavoro, fare una passeggiata al parco, preferire le scale all'ascensore, giocare con i figli ogni giorno sono attività sufficienti per migliorare il proprio stile di vita.

La partecipazione a corsi di attività motoria e sportiva, di ginnastica dolce o trovare un gruppo con cui allenarsi diventano inoltre strumenti importantissimi di socializzazione, condivisione e integrazione. Far parte di un gruppo e allenarsi insieme vuol dire nuove amicizie, nuovi punti d'appoggio, meno isolamento e più comunità, sia per i bambini che per gli adulti.

UISP sportpertutti è un associazione di promozione sociale, un ente di promozione sportiva che vede lo sport come un diritto per tutti i cittadini, indipendentemente dall'età, dal genere, dalle abilità. Sportpertutti significa nessuno escluso: salute, prevenzione, inclusione, educazione. (Uisp, Comitato del Trentino - www.uisp.it)

MARMELLATA di prugne

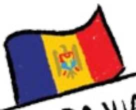

MOLDAVIA

RICETTA DI VERONICA

Ingredienti

1 kg di prugne
300 gr di zucchero

Preparazione

1. Lavate e pulite le prugne togliendo il nocciolo e lasciatele riposare coperte da uno strofinaccio per una notte in modo che facciano un po' di liquido.

2. Mettete le prugne e il loro liquido in una pentola e aggiungete lo zucchero.

3. Cuocete a fuoco medio, girando con un mestolo di legno, per almeno 1 ora o finché la marmellata non raggiunge la consistenza che si desidera.

In Moldavia è piuttosto comune preparare le conserve per l'inverno. Con le stesse dosi si può preparare la marmellata d'uva a cui si aggiunge un ulteriore passaggio, filtrando la marmellata in un colino a maglie strette per togliere i semi. Per la conservazione, terminata la cottura versate la marmellata in contenitori di vetro (lasciando uno o due centimetri dal bordo), a caldo, coprendola con un disco di carta oleata. Chiudete i vasi e capovolgeteli, lasciandoli raffreddare su una superficie piana.

BAGHRIR

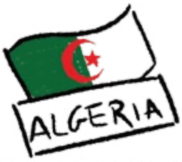

RICETTA DI NADIA

Ingredienti *(per 4 persone)*

1 tazza di semola di grado duro rimacinata
1,5 tazze di acqua tiepida
10 gr di lievito di birra
sale

Preparazione

1. Mettete la semola, il sale, l'acqua e il lievito di birra in un recipiente alto. Frullate tutto con il mixer e fate riposare l'impasto coperto per circa un'ora.

2. In una padella ben calda, appena unta d'olio, versate un mestolo di impasto e fate cuocere qualche minuto a fuoco medio, senza girare. A contatto con il calore vedrete comparire tanti buchini. Man mano che sono pronti, appoggiate i baghrir su un piatto e copriteli con uno strofinaccio.

3. Potete cospargere i baghrir con un velo di miele, piegarli in quattro e servirli insieme a una tazza di tè verde e foglie di menta fresca.

I baghrir sono simili alle crepes, fatti di semola fine, sono tipici dei paesi del Maghreb. Si mangiano a colazione o come spuntino a merenda. Per la preparazione potete usare poco meno di un cubetto di lievito di birra oppure lievito secco con una punta di zucchero.

Dolci
e bevande

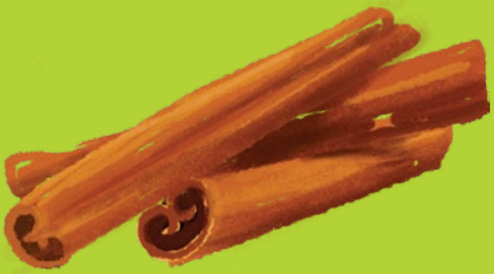

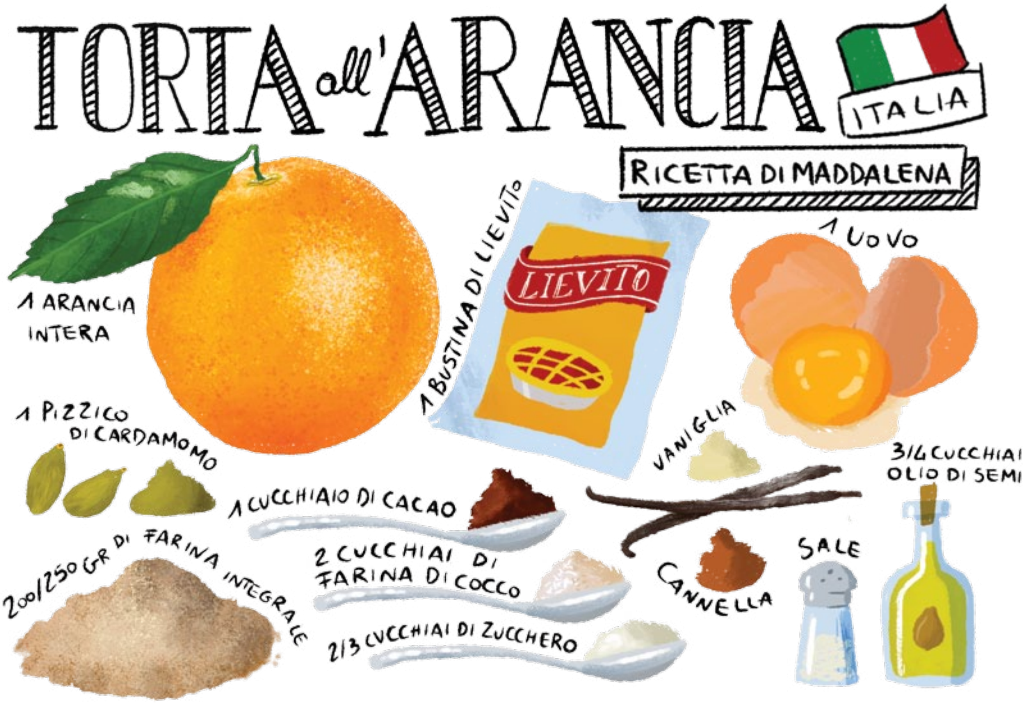

Ingredienti

200/250 gr farina integrale
1 arancia intera
1 uovo
2/3 cucchiai di zucchero
3/4 cucchiai di olio di semi
1 bustina di lievito
2 cucchiai di farina di cocco
1 cucchiaio di cacao
1 pizzico di vaniglia
1 pizzico di cardamomo
1 pizzico di cannella
1 pizzico di sale

Preparazione

1. Per preparare questo dolce si usa un'arancia intera con la buccia. Lavatela bene, tagliatela a pezzetti (intera, senza sbucciarla) e mettetela nel mixer insieme a zucchero, uova, olio di semi, sale. Frullate gli ingredienti qualche minuto per ottenere un composto cremoso.

2. Aggiungete lievito, cacao, farina di cocco e la farina integrale poco per volta. Mescolate con un mestolo di legno o con le fruste, incorporando la quantità di farina necessaria per ottenere un impasto morbido. Alla fine aggiungete un pizzico di cardamomo e cannella.

3. Quando l'impasto ha la giusta consistenza versatelo in uno stampo per dolci, foderato con carta da forno, e infornate a 180° per 30-40 minuti. Per verificare che sia pronta potete fare la prova stecchino: infilate uno stecchino al centro della torta, se quando lo estraete è asciutto la torta è cotta, se è ancora umido lasciatela altri 5 minuti in forno.

Nella preparazione di questa torta l'arancia intera frullata sostituisce la componente liquida del latte. La quantità di farina dipende dall'arancia (dimensione, più o meno succo, spessore della buccia) per cui aggiungete la farina a cucchiai, poca per volta, fino a ottenere un impasto morbido. Il procedimento è molto semplice, il risultato è una torta soffice, leggera e profumata: arancia, cioccolato, cannella e cardamomo sono un'ottima combinazione di sapori.

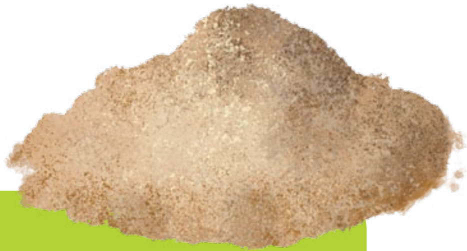

Scegliere farine integrali

È preferibile usare farina integrale o, in base alla ricetta, farina tipo 2 o 1 per le sostanze nutritive e le fibre che contengono.
La farina integrale contiene amido, crusca e germe di grano mentre la farina 00 è quella più raffinata e contiene solo amido e poche proteine. Sostituire farina 00 con altre farine nella preparazione dei piatti non è automatico ma si può fare qualche prova per capire come variare le quantità.
Alcune ricette contenute nel libro sono fatte con semola di grado duro rimacinata, farina di miglio, mais bianco, platano, riso.
Nei reparti ben forniti dei negozi potete trovare altri tipi di farina. Una fra tutte: la farina di ceci che, unita all'acqua, diventa una pastella con cui sostituire le uova in alcune preparazioni come per esempio le frittate o le crêpes.

GNOCCHI di patate e prugne

TRANSILVANIA

RICETTA DI EMILIA

Ingredienti

1 kg di patate da gnocchi
300 gr di farina
1 uovo
1 cucchiaio di burro
200 gr di prugne
180 gr di pangrattato
cannella in polvere
zucchero di canna
5/6 noci
olio di semi
sale

Preparazione

1. Pelate le patate, tagliatele a metà e mettetele in una pentola. Coprite d'acqua, aggiungete un po' di sale grosso e fatele bollire. Quando sono pronte, ancora calde, schiacciate le patate come quando fate il purè e fatele raffreddare. In una ciotola mettete le patate schiacciate, l'uovo, la farina, 1 cucchiaio di burro ammorbidito (non sciolto), 1 pizzico di sale e amalgamate bene gli ingredienti.

2. Nel frattempo, lavate le prugne, apritele a metà e togliete il nocciolo.

3. Su un asse di legno o sul tavolo da cucina stendete l'impasto di patate con il mattarello fino a ottenere uno spessore di ½ cm circa. Con una rotellina (come quella per fare i ravioli), o un coltello, fate dei quadrati di 5x5 cm circa. Al centro di ciascun quadrato mettete 1 prugna (o ½, se è grande). Spolverate con un po' di cannella e un filo di zucchero di canna. Richiudete l'impasto e, lavorandolo con le mani, fatene una pallina, come fosse una polpetta.

4. In una padella antiaderente fate tostare il pangrattato, senza farlo abbrustolire.

5. Prendete una pentola capiente, fate bollire dell'acqua: abbassate la fiamma e immergete gli gnocchi. Fate attenzione che non attacchino sul fondo, mescolando piano con un mestolo. Da quando gli gnocchi vengono a galla, fate cuocere per qualche minuto.

6. Scolate gli gnocchi uno alla volta e passateli nel pangrattato, girandoli delicatamente. Quando sono pronti, serviteli con una spolverata di zucchero e qualche pezzetto di noce.

Gli gnocchi sembrano sempre facili da fare, poi a volte si disfano nell'acqua. Ecco tre consigli: scegliete patate adatte agli gnocchi, facendovi consigliare prima di acquistarle; quando sono cotte, dopo aver schiacciato le patate, fatele raffreddare; durante la cottura, quando l'acqua bolle, abbassate la fiamma prima di immergere gli gnocchi, poi fateli cuocere a fuoco medio. Con la stessa ricetta si possono fare gli gnocchi di albicocche e, al posto delle noci, potete usare semi di papavero. Le patate possono essere lessate o cotte al vapore e l'aggiunta di burro all'impasto è facoltativa.

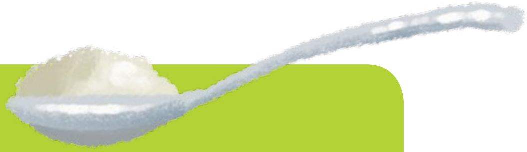

Evitare i cibi spazzatura e le bevande zuccherate

Il consumo elevato di cibi industriali altamente processati (cibi spazzatura) è una delle cause maggiori di obesità, con effetti negativi per la salute, e comporta il rischio di ammalarsi di diabete, malattie cardiovascolari e alcune forme di cancro.
Per una sana alimentazione è importante evitare completamente le bevande zuccherate e i cibi spazzatura (prodotti industriali con molte calorie e pochi nutrienti).
Lo zucchero comune prende il nome di saccarosio: è composto da una molecola di fruttosio e una di glucosio, e viene ricavato sia dalla barbabietola che dalla canna da zucchero.
Nei cibi confezionati, occorre fare attenzione agli ingredienti che terminano in "osio" (fruttosio, destrosio, maltosio): sciroppi, melassa e concentrati di succo di frutta sono solo alcuni esempi di prodotti che contengono zuccheri semplici. Questi zuccheri sono spesso nascosti nei cibi e nelle bevande: per esempio, in 1 lattina di bevanda zuccherata sono presenti fino a 40 gr di zucchero (circa 10 cucchiaini).
Considerato che l'OMS (Organizzazione mondiale della sanità) raccomanda di non superare il 10% delle calorie complessive giornaliere di zuccheri aggiunti (meglio ancora il 5%, pari a 6 cucchiaini di zucchero) il consumo di questi prodotti andrebbe molto ridotto. Mentre, per evitare i cibi spazzatura possiamo ricordare alcuni consigli di Michael Pollan: evita cibi e bevande che hanno lo stesso nome in tutto il mondo, non comprare cibi e bevande pubblicizzati in tv, non comprare cibi industriali con più di 5 ingredienti o che riportano sulla confezione la scritta "fa bene alla salute".
[Michael Pollan, In difesa del cibo, Adelphi, 2009]

BUDINO di RISO

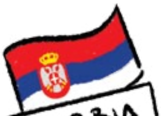

SERBIA

RICETTA DI MARIJA E DEJAN

Ingredienti *(per 4 persone)*

2 tazze di riso
1 l di latte
2 cucchiai di zucchero di canna

Preparazione

1. Sciacquate il riso sotto l'acqua fredda in modo da togliere l'amido.

2. In una pentola antiaderente, mettete il riso e coprite appena d'acqua fredda. Potete calcolare 1 tazza e ½ di acqua per ogni tazza di riso. Fate cuocere a fuoco medio senza mescolare. Quando l'acqua si è consumata, aggiungete latte tiepido, zucchero di canna e proseguite la cottura per 15 minuti a fiamma bassa. Riso e latte, cuocendo, diventano una crema.

3. Quando il budino sarà pronto versatelo nelle ciotoline da dessert.

Il budino di riso è buono sia caldo che freddo. È fatto di pochi ingredienti, si può mangiare sia a colazione che a merenda. Il riso dolce è un dessert presente nella cucina di molti paesi: Pakistan, India, Turchia, Grecia, Spagna, Inghilterra, Italia, Argentina. Ogni area geografica ha la sua ricetta: con o senza cannella, vaniglia, buccia di limone, acqua di fiori d'arancio o rosa, miele, pistacchi, uvetta, mandorle, semi di cardamomo, zafferano, anacardi. Anche sul riso prevale la varietà: c'è il carnaroli, il vialone nano, il basmati. Dipende dai paesi, dalle zone, dalle tradizioni della famiglia. È un piatto che ricorda l'infanzia, viene da lontano. Sembra una ricetta facile ma per fare un riso dolce e cremoso ci vuole cura.

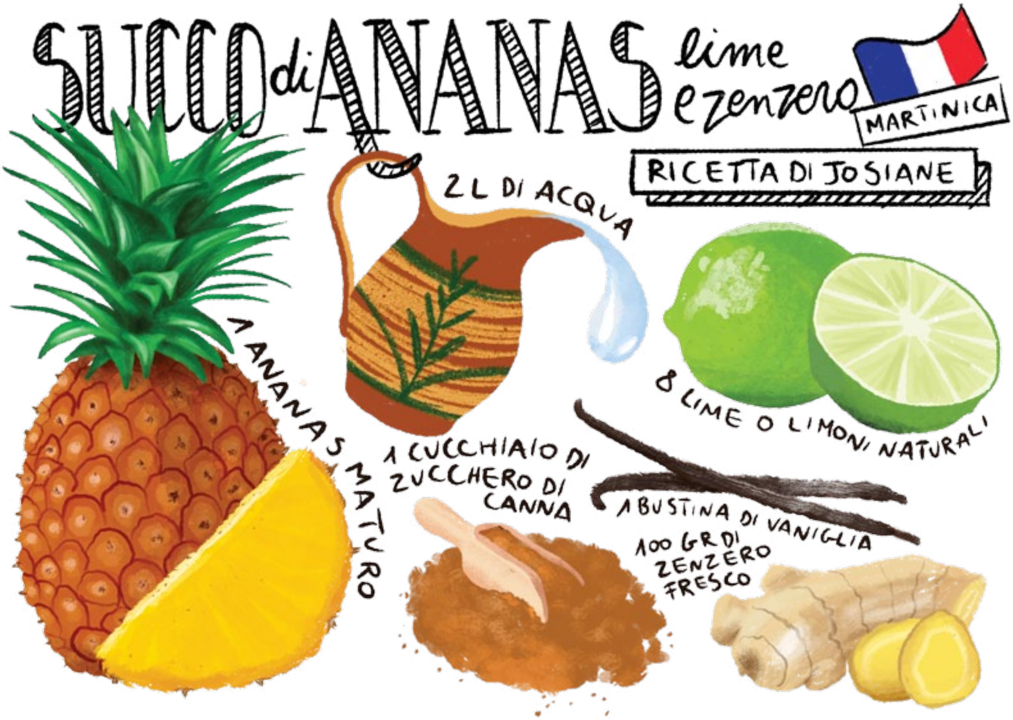

Ingredienti

1 ananas maturo
100 gr di zenzero fresco
8 lime o limoni naturali
2 l d'acqua
1 cucchiaio di zucchero di canna
1 bustina di vaniglia (facoltativo)

Preparazione

1. Sbucciate l'ananas e lo zenzero. Tagliate tutto a pezzetti e frullate insieme.

2. Con un colino filtrate il succo raccogliendolo in una caraffa. Versate l'acqua nel colino e schiacciate la polpa rimasta all'interno con un mestolo di legno. Filtrate di nuovo, aggiungete il succo di lime che avrete filtrato a sua volta, la vaniglia, lo zucchero di canna e mescolate.

Al posto dell'ananas si possono usare 8 arance (circa 1 kg) spremute. Per ottenere un succo limpido, senza pezzettini, la frutta viene frullata, aggiungendo i pezzetti un po' alla volta, e filtrata più volte con un colino a maglie strette. In questo modo si ottiene un cocktail analcolico e dissetante.

FRULLATO di banana

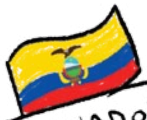

ECUADOR

RICETTA DI MIRIAM

Ingredienti

1 banana matura
2 tazze di latte
3 cubetti di ghiaccio
1 cucchiaino di miele

Preparazione

1. La preparazione è molto semplice: si frulla la banana insieme al latte e un po' di miele, infine si aggiunge qualche cubetto di ghiaccio.

In Ecuador a colazione si mangia spesso frutta fresca, yogurt bianco, pane integrale e non manca mai un frullato di frutta di stagione.

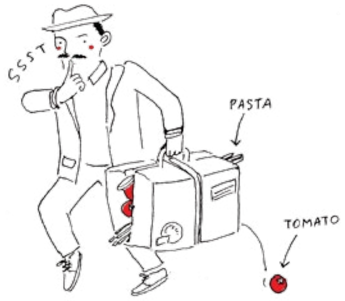

Tomatology, la strana storia del vegetale più famoso del mondo

Questo buffo frutto rotondo oggi è un indiscusso protagonista delle cucine di quasi tutto il mondo. Per non parlare delle prelibatezze della incoronata penisola: pizza e pasta danno il meglio tinte dal pomodoro. Ma la vita del rosso vegetale non è sempre stata così.
Il pomodoro nasce nel nord del Sud America, e le popolazioni Maya e poi Azteche se ne prendono cura fino a farlo crescere grande e succoso. Loro sono i primi a cuocere il pomodoro in salse. Finché intorno al 1500 arrivarono i conquistadores europei, dei quali non possiamo andare tanto fieri. Tra altre razzie e devastazioni, ori e preziosità, si rubarono pure il pomodoro. Ebbene sì. Direte voi, ma che gli importava della verdura!? All'epoca, tra re e ricchi mecenati europei andava di moda sfidarsi a colpi botanici: chi sfoggiava il giardino più rigoglioso e ricco di piante esotiche vinceva il massimo prestigio. Ed il pomodoro, con quelle belle palle rosse attaccate, era proprio una pianta strana, degna del giardino di un re.
Il pomodoro, appena arrivato in Europa, trova impiego come pianta decorativa e se ne resta fuori in cortile. In cucina non può entrare… sia mai! I botanici lo consideravano pericoloso, una verdura del colore sbagliato: troppo simile alla belladonna e alla melanzana, piante velenose. Addirittura il dottor Giovanni Medico Sale nel 1600 dice 'solo un folle può mangiare quei frutti!'. E in effetti qualche folle c'era, e verso la fine del 1700 qualcuno si azzarda ad assaggiarlo. Iniziano a cuocere in pentola le prime timide salse.
Nel 1800 però avvenne un incontro fatidico: a Napoli il pomodoro incontra la pasta! Ed è subito amore. Da quel momento in poi per il pomodoro si spalanca la via! Tutte le cucine gli aprono le porte e tutti i piatti si tingono di rosso.
Dopo quasi trecento anni di attesa il sudamericano frutto è riconosciuto nelle sue preziose virtù. Il pomodoro oggi è una star! (testo e immagini tratti dalla graphic novel: TOMATOLOGY, la strana storia del vegetale più famoso del mondo, di Sara Filippi Plotegher)

Le tue ricette

Ringraziamenti

Le ricette raccolte in questo libro sono di Wassila Abassi, Patience Aiwerioba, Hanane Akarchaf, Redouane Belkouch, Fouzia Bendra, Alisha e Zainab Bhatti, Miriam Chamba, Shabnam Chhimpa, Veronica Ciubotaru, Emilia Csiki, Amal El Fathi, Pirous Fateh-Moghadam, Xueyan Fu, Michał Gołabek, Diana Gurgu, Mina Igli, Nadia Idri, Elsa Kadi, Meryem Koudiche, Viktoriia Kusturova, Yaquelin Labrade, Mack M'Baye, Safoora e Shoukat Mahmood, Eva Maigua, Jie Yang, Josiane Martin, Beenish Masood, Mariela Murillo, Imeri Nerminka, Magatte Niang, Aminata e Salamata Ouedraogo, Marija Petkovic, Mathurin Sikadi, Aligueton e Rasside Sogne, Fatoumata Togo, Aisha Traore, Halyna Vozna, Maddalena Wegher.

Insieme a loro, altri hanno contribuito fornendo contatti, suggerimenti e qualche ricetta che a volte si è fermata a metà. Grazie a Max Arer, Xin Bavaro, Tommaso Bonazza, Francesca Braito, Farooq Butt, Andrea Cagol, Victor Campero, Franco Castellan, Lisa Dal Mas, Beatrice De Blasi, Anna Eccher, Mor Fall, Monica Ghezzi, Erio Korani, Magdalena Luca, Luigi Mario Mafezzoli, Benedetta Massignan, Mirko Montibeller, Elisa Morgano, Riccardo Mosna, Roshanthi Nicolò, Irina Platon, John Praghakar Salins, Virginia Sartori, Andrea Silli, Bhupinder Singh, Lassana Sissoko, Gaia Trecarichi, Amina, Matar.

Grazie alle mediatrici culturali che ci hanno accompagnato in questo percorso: Xueyan Fu, Amel Hemmal, Jie Yang, Oleya Koroleva, Samira Kousar, Souad Lachhab, Vera Liachenko, Sumera Usman.

Grazie anche al Cinformi e alle associazioni che ci hanno dedicato tempo, idee e spazio per il confronto: associazione culturale Alchemica, associazione Amici della Colombia, associazione italo-moldava Arcobaleno, associazione di promozione sociale Carpe Diem, CRI Comitato provinciale di Trento, associazione di donne straniere Intrecci, associazione Mimosa, FAP-Acli per il Trentino, TrentinoSalute4.0, UISP Unione italiana sport per tutti - Comitato del Trentino, associazione italo-ecuadoregna Ponte di sostegno educativo, associazione cristiano culturale degli ucraini in Trentino Rasom, associazione giovani albanesi Rinia, associazione italo-senegalese Suuf Verde Onlus, associazione Tahuantinsuyu, associazione culturale ViviLavis, comunità nigeriana Trentino-Alto Adige, OrchExtra Terrestre.

Le cucine in cui abbiamo imparato, osservando, sono il Forno sociale Migola di Canova di Gardolo, “Le Formichine” cucina solidale a Rovereto, “Midì” mangiare naturale a Trento oltre all’orto del mondo di Lavis.

Utile e generoso il confronto con gli organizzatori del progetto “CusCus Trento” e il Festival di cinema, cibo e videodiversità “Tutti nello Stesso Piatto” promosso da Mandacarù Onlus e Altromercato.

Per il supporto amministrativo si ringraziano Ombretta Sciandra e Daniela Bonaldi dell’Ufficio Ricerca e innovazione, diretto da Diego Conforti, del Dipartimento Salute e politiche sociali della Provincia autonoma di Trento.

Per gli approfondimenti sulle proprietà dei legumi e dell’olio extravergine di oliva, su come ridurre il sale e il consumo di carne si ringrazia Paola Osti; Maddalena Wegher per il contributo su come usare la soia nella preparazione di alcuni piatti; Monica Ghezzi per i suggerimenti di sana alimentazione per i bambini; Francesca Braito per parlarci di pane, lievito e relazioni; Beatrice Agostini per il contributo su movimento e attività fisica; Rosa Maimone per averci fatto conoscere Meddie e Cleo in una App che favorisce l’abitudine a sani stili di vita; Sara Filippi Plotegher per averci raccontato Tomatology, la strana storia del vegetale più famoso del mondo.

Un vostro riscontro su questo libro è molto gradito. Potete esprimere il vostro giudizio rispondendo a un breve questionario di 5 domande:
https://forms.gle/eJqTmwRhgBpCcmWE6
Grazie per la collaborazione!

Stampato da Litografia EFFE e ERRE
per conto della Provincia autonoma di Trento,
maggio 2019

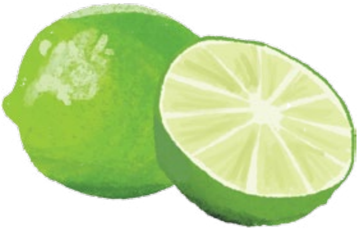

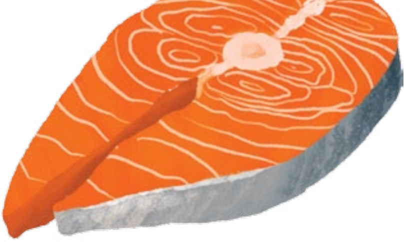

Più di 60 ricette e i sapori della cultura culinaria di 25 paesi. Piatti semplici, ingredienti freschi e cotture leggere. Queste pagine contengono spunti e idee per una sana alimentazione che include ingredienti e preparazioni casalinghe da tutto il mondo. È un invito a preparare cibi sani, scoprire e inventare nuovi piatti, costruire relazioni.

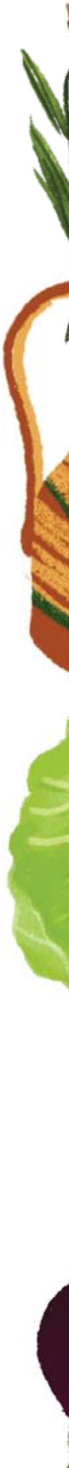

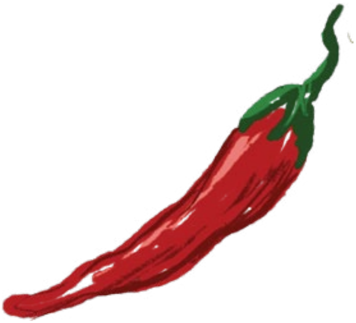

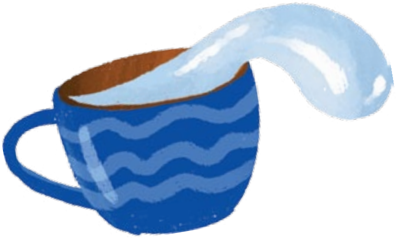

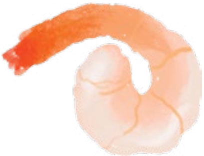

PROVINCIA AUTONOMA DI TRENTO
Dipartimento Salute e Politiche sociali

Azienda Provinciale per i Servizi Sanitari
Provincia Autonoma di Trento

INMP NIHMP

Azienda Ospedaliera Sanitaria Integrata Verona

Südtiroler Sanitätsbetrieb
Azienda Sanitaria dell'Alto Adige
Azienda Sanitera de Sudtirol

FONDAZIONE BRUNO KESSLER

UISP sportpertutti
Comitato del Trentino

Università Ca'Foscari Venezia

Osservatorio per la salute
PROVINCIA AUTONOMA DI TRENTO

Smuovi la salute

ISBN 978-88-7702-472-5

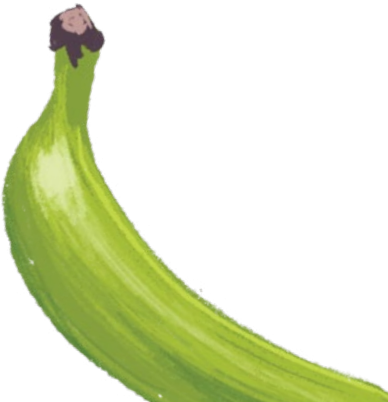

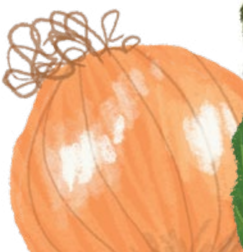

Supplement: Supplementary file 1 [file nutrients-13-03634-s001.zip › Supplementary material Figure S2.pdf]
