# Supplementary material for: Educational Intervention of Healthy Life Promotion for Children with a Migrant Background or at Socioeconomic Disadvantage in the North of Italy: Efficacy of Telematic Tools in Improving Nutritional and Physical Activity Knowledge
Source: Nutrients. 2021 Oct 17;13(10):3634. doi: 10.3390/nu13103634 (PMC8540523; doi:10.3390/nu13103634)
Supplement: Supplementary file 1 [file nutrients-13-03634-s001.zip › Supplementary material Figure S3.pdf]

Smuovi la salute

PROVINCIA AUTONOMA DI TRENTO

Azienda Provinciale per i Servizi Sanitari

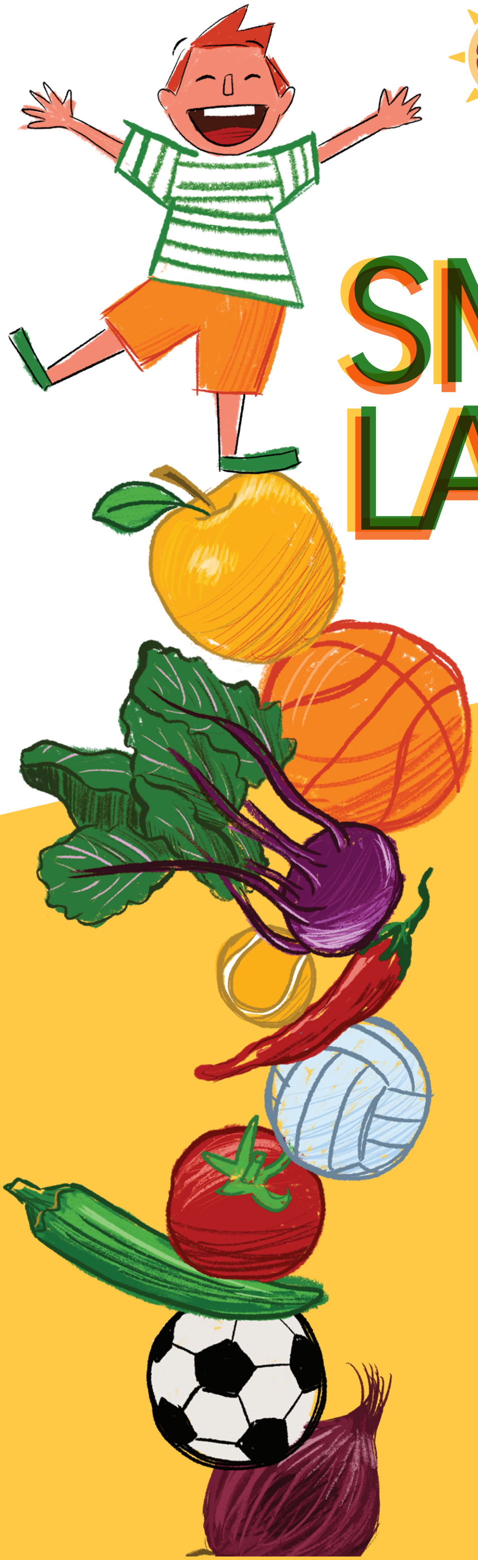

SMUOVI LA SALUTE

A Trento, Bolzano e Verona il progetto “Smuovi la salute” propone **attività per famiglie, bambini e ragazzi fino a 18 anni.** Oltre alle visite in ambulatorio (con pediatra, dietista e psicologo) le attività proposte aiutano ad avere uno stile di vita sano. Muoversi, camminare, giocare, mangiare frutta e verdura, preparare cibi sani, bere acqua al posto delle bevande zuccherate: sono piccole abitudini che fanno stare bene.

informazioni e aggiornamenti
@smuovilasalute

RICETTE SANE DAL MONDO

Da giugno è disponibile il libro **“Cucinare sano alla portata di tutti” con tante ricette per portare in tavola i sapori delle culture culinarie di 25 paesi diversi**. Sono ricette sane, di tutti i giorni, scritte insieme a chi ne conosce i segreti e la preparazione.

trovi il libro su www.trentinosalute.net

per informazioni

Sara Carneri, Pirous Fateh Moghadam
Osservatorio per la salute, PAT
Mail: info@smuovilasalute.it
Cell: 3807107205

GIOCHIAMO NEI PARCHI

Attività ludico-motoria per bambini dai 6 ai 10 anni e dagli 11 ai 13 anni.

martedì, dalle 17.00 alle 18.30
in un parco, in centro città

venerdì, dalle 9.30 alle 11.30
al Parco di Melta, a Gardolo

per informazioni e iscrizioni

Beatrice Agostini
UISP, Comitato del Trentino
Mail: b.agostini.trento@uisp.it
Tel: 0461231128 - Cell: 3474022475

QUIZ E SFIDE IN UNA APP

Scarica l'app e **segui Meddie e a Cleo nella loro avventura** per scoprire principi di alimentazione sana in modo semplice e divertente. Tieni aggiornato il tuo **diario alimentare** per ricevere suggerimenti, avanzare nelle sfide settimanali e conoscere nuove ricette deliziose.

trovi SmuoviLaSalute nel tuo app store

Available on the App Store | GET IT ON Google Play

per informazioni

Mail: smuovilasalute@fbk.eu

MANGIARE SANO: COSA, COME E PERCHÉ

Il cibo è **scoperta, conoscenza, cultura e relazione**. Incontri su sana alimentazione con Monica Ghezzi e Roberto Franceschi.

per informazioni

Roberto Franceschi
SC Pediatria Ospedale S.Chiara di Trento
Mail: roberto.franceschi@apss.tn.it

Smuovi la salute

PROGETTO FINANZIATO DALL'INMP

INMP – Istituto Nazionale per la promozione della salute delle popolazioni Migranti e per il contrasto delle malattie della Povertà
NIHMP – National Institute for Health Migration and Poverty

PROVINCIA AUTONOMA DI TRENTO

UISP sportpertutti – Comitato del Trentino

FONDAZIONE BRUNO KESSLER

Azienda Provinciale per i Servizi Sanitari

Südtiroler Sanitätsbetrieb – Azienda Sanitaria dell'Alto Adige – Azienda Sanitera de Sudtirol

Azienda Ospedaliera Universitaria Integrata Verona

Università Ca'Foscari Venezia

Osservatorio per la salute – PROVINCIA AUTONOMA DI TRENTO
